# Exploring the lived experiences of parents caring for infants with gastroschisis in Rwanda: The untold story

**DOI:** 10.1371/journal.pgph.0000439

**Published:** 2022-06-15

**Authors:** Samuel Kidane, Semay Desta Shamebo, Edmond Ntaganda, Robin T. Petroze, Zahirah McNatt, Rex Wong, Melany Rabideau

**Affiliations:** 1 Bill and Joyce Cummings Institute of Global Health, University of Global Health Equity, Butaro, Rwanda; 2 Department of Surgery, Centre Hospitalier Universitaire de Kigali, Kigali, Rwanda; 3 Division of Pediatric Surgery, Department of Surgery, University of Florida, Gainesville, Florida, United States of America; 4 Department of Community Health and Social Medicine, University of Global Health Equity, Butaro, Rwanda; 5 School of Public Health, Yale University, New Haven, Connecticut, United States of America; Harvard Medical School, UNITED STATES

## Abstract

Pediatric surgery is a crucial pillar of health equity but is often not prioritized in the global health agenda, especially in low-and middle-income countries. Gastroschisis (GS) is a type of structural congenital anomaly that can be treated through surgical interventions. In Rwanda, neonatal surgical care is only available in one hospital. The experience of parents of children born with gastroschisis has not been previously studied in Rwanda. The objective of this study was to explore the lived experiences of parents of children diagnosed with GS in Rwanda. A qualitative study using a semi-structured interview guide was conducted. Parents who had children with gastroschisis and were discharged alive from the hospital in Rwanda were interviewed by trained data collectors, from May to July 2021. Data were transcribed, translated, and then coded using a structured code-book. Thematic analysis was conducted with the use of Dedoose software. Sixteen parents participated in the study. Five themes emerged from the data. They were: “GS diagnosis had a significant emotional impact on the parents”, “Parents were content with the life-saving medical care provided for their children despite some dissatisfaction due to the delayed initiation of care and shortage of medications”, “GS care was accompanied by financial challenges”, “support systems were important coping mechanisms” and “the impact of GS care extended into the post-discharge period”. Having a newborn with GS was an emotional journey. The lack of pre-knowledge about the condition created a shock to the parents. Parents found support from their faith and other parents with similar experiences. The experiences with the care received were mostly positive. The overall financial burden incurred from the medical treatment and indirect costs was high and extended beyond the hospital stay. Strengthening prenatal and hospital services, providing peer, spiritual and financial support could enhance the parents’ experience.

## Introduction

Pediatric surgery is a crucial pillar of health equity; yet an estimated 1.7 billion children, particularly neonates, lack safe, timely, and affordable access to surgical care [1]. Across the globe, the complex nature of congenital anomalies contributed to a significant percentage of neonatal mortality [2]. Out of the mortality that is attributed to congenital anomalies, gastroschisis (GS) is one of the leading causes [3].

GS is a congenital defect in the abdominal wall among newborns; the intestines of newborns with GS protrude externally through the defect [4]. The global incidence of GS was estimated to be 1 in 2000 births [3]. The risk factors of GS have yet to be identified, although some studies had suggested young maternal age and low socioeconomic status were linked to the condition. Newborns with GS should receive immediate surgical management within a few days after birth to survive and achieve better health outcomes [5]. With a survival rate of 95% and less than 5% in high-income countries and low- and middle-income countries (LMIC) respectively, there is a significant disparity in immediate outcomes of infants with GS globally [3].

Studies have shown that newborns with GS who survive after surgical treatment would continue to face subsequent difficulties. Many experienced dietary issues in their infancy and half of them required additional surgeries [6]. However, the impacts of the condition are not only limited to the patients but also their families, especially their parents or caregivers [7]. A qualitative study conducted by Drotar et al in 1975 [8], suggested that parents, especially mothers, were exposed to some emotional responses; the guilt and sense of hesitancy at the initial stage would result in subsequent detachment from their babies. However, the study was conducted over four decades ago and no subsequent studies were found to quantify such findings. Besides, the parents’ experience and responses toward babies born with GS, beyond the emotional aspect, was not studied, particularly no publication in this area was found in Rwanda.

The children’s survival and overall quality of care received were affected by many factors. Understanding the challenges faced by the parents or caregivers is crucial to inform the design of appropriate interventions to address their needs, and the overall quality of care provision.

In Sub-Saharan Africa (SSA), specifically Rwanda, there is a gap in the peer-reviewed literature concerning the parental experiences and the challenges they faced during the care for their infants with GS. Such a knowledge gap directly impacts the quality of GS care both in the hospital and in the community settings. This study aimed to provide insights into the challenges faced by the parents throughout their children’s GS care. This study attempted to help fill the evidence gap as it pertains to parental experiences of gastroschisis and associated medical care. The study results could inform service providers, program implementers, and policymakers to design appropriate interventions that can improve the quality-of-service delivery as well as the overall experience of patients and their families. The study results could potentially serve as a proxy and the practical implications could be applied to other pediatric surgical cases.

This study was informed by the “Theory of Planned Behavior” (TPB), which is a decision-making model, widely employed to examine the psychosocial influences on behavior. TPB proposes that individuals will have the intention to perform a behavior when they view it positively, believe that others think they should perform it, and perceive it to be within their control [9].

**Fig 1:**
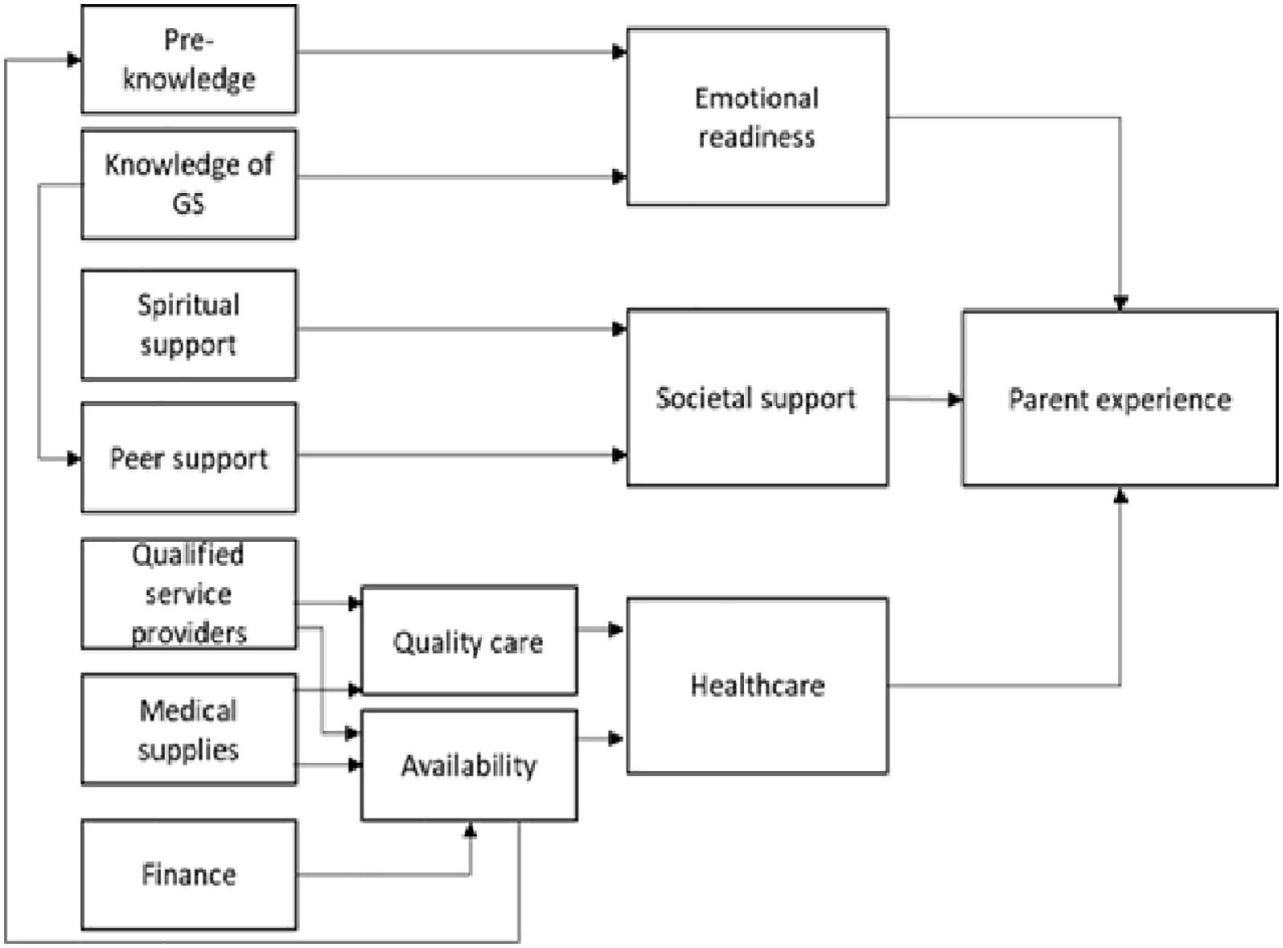
Logic model of the study.

Based on the previous studies, the logic model to guide this study hypothesized that the parents’ experiences were influenced by their emotional readiness, their needs and support received, as well as the medical healthcare their newborns received. With various factors associated with each of these influencers (Fig 1).

## Materials and method

### Setting

The study was conducted in Rwanda. The population of Rwanda was estimated to be almost 13 million in 2021 and the life expectancy at birth was projected to be 67.8 years [10]. In 2019, infant mortality was 33 per 1,000 live births and neonatal mortality was 19 per 1,000 live births [11]. The healthcare system of Rwanda is decentralized to provincial and district levels and a vast majority of the population were covered by community health insurance [12]. Pediatric surgery is one of the priorities of the surgical workforce development in Rwanda [13]. Centre Hospitalier Universitaire de Kigali (CHUK), located in the capital city, is the only public hospital that offers comprehensive pediatric and neonatal surgical care in the country. The hospital has over 500 beds and provides a vast range of clinical services [14].

### Study design and sample

A qualitative study using a phenomenological approach was conducted. The study explored the lived experience of parents of infants with GS, using in-depth interviews (IDI) to collect data. All parents and caregivers of neonates who received GS care and were discharged alive from CHUK between 2020 and 2021, were invited as the sample until theoretical saturation was attained [15]. The post-discharge time ranged from 1 to 17 months, and one baby had passed away since being discharged from the hospital.

### Data collection tools and procedures

A semi-structured in-depth interview guide was developed to facilitate the data collection based on the objectives of the study. The interview questions centered around the experience of the participants in caring for their children with GS and the contexts that affected their experience. Six open-ended questions with follow-up probes for each question were included in the interview guide. The guide was developed in English, then translated to Kinyarwanda (the local language), and back-translated by two persons who are fluent in both languages.

The hospital medical registration database was accessed by the treating physicians to identify GS cases that were discharged alive after undergoing surgical care at the study site. The contact phone numbers of the eligible participants were also collected. The research team contacted the eligible parents via phone to preliminarily explain the study and sought their agreement to participate. The data collectors emphasized to the potential participants that the study team was not affiliated with the hospital, to minimize their fear of being coerced to participate, and their refusal to participate would not affect their children’s future treatment at the hospital in any way. Appointments were set for those who were willing to participate, at a time and location of their choice. They could also choose between in-person or phone interviews according to their preference. At the agreed appointment date and time, detailed information about the study including objectives, interview process, the significance of the study, the use of information, and their rights to participate or withdraw from participation were provided to all participants, before informed consent was collected. Written informed consents were collected for in-person interviews, and verbal consents were collected for the phone interviews. All interviews were administered in Kinyarwanda by two data collectors. Parents were interviewed either individually or in pairs, depending on their preference and availability. Permission to audio record the interview was also sought. Each interview lasted approximately 45 minutes.

The study was approved by the University of Global Health Equity (Ref: UGHE-IRB/2021/038) and Centre Hospitalier Universitaire de Kigali (Ref:EC/CHUK/067/2021) Institutional Review Boards.

### Data management and analysis

Following the completion of data collection, transcription, and translation, verification of transcribed and translated transcripts were undertaken by data collectors fluent in both English and Kinyarwanda (S1 Data). To increase the rigor of the study, some data were triangulated through discussion with two hospital staff to verify the information related to the hospital. The researchers held regular meetings during the process of analysis. An initial codebook was developed after the open reading of the transcripts by the researchers. The codes were revised and refined after thorough discussions and agreement among researchers. Thematic analysis was used in this study. The consolidated criteria for reporting qualitative research (COREQ) framework was used throughout the study to guide the research process [16].

The transcripts were read by all researchers, first individually then jointly to ensure agreement on the final coding. Dedoose software V 9.0.15 was used to organize the data for analysis. Codes were organized into categories, then into themes. Excerpts were extracted from the transcripts to illustrate the themes.

## Results

A total of 11 interviews with 16 participants (5 couples and 6 mothers) were conducted. All participants were the biological parents of the children with GS. All participants had their children with GS discharged alive from CHUK. At the time of the study, one child had passed away a few weeks after being discharged. The children were aged between 6 weeks to 18 months. The mothers were aged between 20 to 41 years. All the participants were covered by community-based health insurance (Table 1).

**Table 1:** Sociodemographic characteristics of the participants.

Characteristics	Categories	N (%)
Interview	Couple (father and mother)	5 (45%)
	Individual (mother)	6 (55%)
Age of mother	< 25 years	6 (55%)
	25 and above	5 (45%)
Age of children	< 12 weeks	4 (36%)
	3–6 months	4 (36%)
	7–11 months	1 (9%)
	1–2 year	2 (19%)
Survival status of children	Dead	1 (9%)
	Alive	10 (91%)
Insurance	Yes	11 (100%)
	No	0 (0%)

The analysis of the transcripts resulted in 21 codes. Five overarching themes emerged:

1. GS diagnosis had a significant emotional impact on the parents.
2. Parents were content with the life-saving medical care provided for their children despite some dissatisfaction due to the delayed initiation of care and shortage of medications.
3. GS care was accompanied by financial challenges.
4. Support systems and religious faith were important coping mechanisms.
5. The impact of GS extended beyond the hospital.

### Theme 1: GS diagnosis had a significant emotional impact on the parents

The parents expressed fear and surprise as their initial reactions to finding out their children were born with GS. None of the parents were aware that their children had this congenital condition before they were born. During their antenatal care (ANC) visits, they were told their children were normal. As a result, they were expecting normal and healthy babies. None of them had any knowledge or information about GS.

*“I wondered because I went through the echography while I was pregnant for six months*, *and they never told me that the baby had a problem*, *they told me that the baby was healthy and alive with no problems*.*” Mother of a one*-*month*-*old male infant*.

Without any pre-knowledge, many parents were shocked and scared by the visual presentation of the condition. Some of them said they could not even look at their babies. Naturally, many worried if their babies could survive.

*“After I gave birth to her at the health center*, *they told me to look at the other side*, *to prevent me from looking at my child*…*” Mother of a three*-*and*-*half*-*month*-*old male infant*.

*“I could not imagine how a baby born with external intestines would survive*! *I never imagined that he would be able to live*!*” Mother of a one year and five*-*month*-*old male toddler*.

Some parents said they felt remorse or guilt about the condition, mostly because of the misconception or beliefs about the cause of GS. Very often, the community, including themselves, generally believe that congenital defects were caused by witchcraft, poison, working too much, or prolonged sitting in a specific position during pregnancy, premature delivery, God’s punishment because of the parents’ sins or misdeed, or due to not wanting the pregnancy. Since GS is a rare and visually terrifying condition, they felt the community applied such belief toward them even more than the other congenital conditions. As a result, many felt the babies’ condition was a result of their faults.

*“After seeing that I had given birth to such a child*… *I thought it was something linked to witchcraft*.*” Mother of a five*-*and*- *half*-*months*-*old male infant*.

*“I felt that I was killing my child*… *[I thought] the reason [as] to why she was born like that was [because] I hated kids*…*” Mother of a one year and four*-*month*-*old female toddler*.

A few respondents also expressed their acceptance of their babies’ conditions as part of God’s will.

*[I thought] even though the child would probably not make it*, *God would give us another one*… *[When] they [the doctors] went to show me the baby*, *[I] looked at him and said it is fine*… *I waited for what God would do so that we may return [home] safe*. *Even if we do not return [with our baby] alive*, *then that will be God*’*s will*. *Mother of a seven*-*month*-*old male infant*.

*“*…*after reaching CHUK and seeing other children*, *and realizing that the disease was existent as I heard it from doctors*, *I accepted it and said that she [my child] belonged to God*. *If she*’*s meant to be mine*, *she*’*ll be mine*, *and if she is not meant to be mine*, *she won*’*t be mine*, *that*’*s what I told God and that*’*s how I accepted it*. *Mother of a two*-*month*-*old female infant*.

*“That [GS] is a normal incidence because the God who creates as He wants is the God who creates as He wills*, *in brief*. *Hmm*, *God creates as He wishes*…*”*. *Mother of a one*-*month*-*old male infant*.

Overall, learning about the GS condition the first time after the babies were born caused surprise, shock, and emotional toll on the parents. Not being fully informed ahead of time, they were not emotionally prepared to face the condition.

### Theme 2: Parents were content with the life-saving medical care provided for their children despite some dissatisfaction due to the delayed initiation of care and shortage of medications

Parents generally were grateful for the care their babies had received, despite some areas that needed improvement. Since CHUK is the only referral hospital that has the capacity for providing GS care, all GS babies delivered at other health facilities had to be immediately referred to the CHUK, usually within hours of birth. However, even though the referral was expedited, traveling from some remote regions needed time and that could cause a delay in the initiation of treatment. Parents were naturally worried and not satisfied with such delay. But their perception changed relatively quickly once they had arrived at CHUK. They expressed gratitude to the doctors and nurses for how quickly they had initiated treatment for their babies, and described them as welcoming, committed, and even miracle workers.

*“It was just hard*; *it was hard because you see we got to CHUK and the doctors who looked at him were scared because the journey took us seven hours on the way from Nyamasheke to CHUK*.*” Father of a one*-*month*-*old male infant*.

*My thought is that doctors work with God to recreate since I used to think that it was an incurable disease*. *Now I accept that nothing is impossible*, *I was amazed by the skills of those doctors*, *I can even testify that CHUK really has good doctors*. *Mother of a two*-*and*-*a*-*half*-*month*-*old male infant*.

Parents were also grateful that they were allowed to be part of their babies’ care. Actively engaged in the care made them feel they were involved in saving their babies’ lives and helped them bond with their babies. The healthcare providers at CHUK taught them how to feed the babies, and how to keep the abdominal area clean.

*“*…*but for it [medical care] to go well*, *you*, *the person with the patient*, *was the one who played the biggest role because you were the one in charge of his [the child*’*s] cleanliness*, *who took care of him*, *looked after him*, *changed him*, *plenty of things indeed*, *and the doctors were ever there for your child*.*” Mother of a seven*-*month*-*old male infant*.

However, not all experiences were positive. Some parents expressed facing a shortage of medications and nursing staff. Very often the medications they needed were not available in the hospital, and they had to purchase them from outside the hospital. Some also said the cleanliness of the hospital environment could be improved. The lack of space and shortage of nursing staff was also mentioned by some parents.

*“*…*there were times when there was a shortage of medicine*, *so you go out and look for it*.*” Mother of a seven*-*month*-*old male infant*.

*“*… *the room [NICU] was so small*… *You find that if the children are eight and the nurses are three*, *they have to take care of all the children*.*" Mother of a four*-*month old male infant*.

*“The first thing that discouraged me with this baby was the day I was sitting*… *and saw ants over her lamp*, *and moving down to her navel*. *I felt scared*… *Mother of a one year and four*-*month*-*old female toddler*.

Overall, the parents felt their babies had received life-saving care at CHUK, despite the environmental and systemic limitations.

### Theme 3: GS care was accompanied by financial challenges

Despite the fact that all the participants had health insurance, the participants emphasized the financial burden. In Rwanda, the Community-based Health Insurance covered 90% of the medical charges. However, the 10% balance that the parents were responsible for could impose a significant financial burden on them and their families, depending on their socioeconomic status.

There were many incidents when the medications were not available at the hospital, they had to purchase them at private pharmacies. Such out-of-pocket expense was not reimbursable by the Community-based Health Insurance.

In addition to the direct medical expenses, parents had to either take time away from work or quit their jobs to take care of their babies. Many were bankrupted due to the expenses for food, transportation, and lodging on top of the loss of wages. Taking out loans and selling their lands and livestock were their means to finance their direct and indirect expenses.

*“You just need a lot [of money]*, *whether medications*, *different types of serums that we had to buy from outside and CBHI was not covering those medications*. *We had to buy these medications outside of CHUK*, *and the full cost was on us*. *So*, *when I was discharged*, *I went home with many debts*. *I had a plot of land and sold it*!*” Mother of a one year and five*-*month* -*old male toddler*.

*We use CBHI and even though it*’*s CBHI*, *one is still charged a lot of money*, *but they told me that I leave the place while having cleared the bills of 100*,*000Rwf*. *The one who is not under CBHI pays for 1*,*000*,*000RWF*.*” Mother of a two*-*and*-*a*-*half*-*month*-*old male infant*.

*“The hardest part for me was that I was not financially stable*, *and I needed to eat breakfast and dinner*. *It was also my first time fulfilling these responsibilities since it was my first time giving birth*. *It was very difficult for me*, *and I did not even have a job*.*” Mother of a three*-*and*-*a*-*half*-*month*-*old male infant*.

Overall, parents expressed the heavy financial burden of having a baby with GS, both direct and indirect medical expenses extended from pre-hospitalization to post-discharge.

### Theme 4: Support systems and religious faith were important coping mechanisms

During this challenging and emotionally taxing time in their lives, many parents managed to find comfort and support through a combination of sources. Faith played a significant role. Most of them said they were sustained and strengthened due to their faith in God. They allotted the survival of their children as divine intervention from God.

*“After reaching CHUK*, *we found their other children like that and we realized that many children were dying right after having their intestines fixed*. *It was really hard to accept it*, *but God has done wonders for us so*… *It was God who fixed it [GS]*.*” Mother of a one*-*month*-*old male infant*.

*“So*, *I started accepting it [GS] and pleaded [with] God to assist*, *where the doctors have done their part*, *God also has to play his part*.*” Mother of a two*-*and*-*half*-*month*-*old male infant*.

They also felt the support from the dedicated health professionals—both through the actual medical care they provided and their encouragement.

*“The doctor told me not to worry*. *They told me that doctors are treating him and even though the baby is born with this problem*, *he will recover*. *He kept telling me how they do it*, *and I felt comforted*.*” Mother of a four*-*month*-*old male infant*.

Participants expressed they had received support from their communities, spouses, work, and other parents going through the same experiences, ranging from encouraging words, getting time off from work, financial support, to receiving sustenance from hospital volunteers.

*“You understand that here at home the economy [went] down*, *but we have had friends and one who visited me could bring a kilogram of sugar and life would go on*. *But you understand that I was helped by others who were close to me and supported me*.*” Mother of a four*-*month*-*old male infant*.

*“They [doctors] told us that the babies born with this case recovered*. *Many people gave testimonies about how so many children born with the same case as ours had healed*, *hmmm*, *that was what comforted us plus praying*.*” Mother of a one*-*month*-*old male infant*.

*“I was around when he was born*. *I saw that we gave birth to a child with a problem*. *But in collaboration*, *since I am married to this woman legally and religiously*, *the relationship of a couple should be sticking together through thick and thin*…*” Father of a two*-*and*-*half*-*month*-*old male infant*.

Parents found the support from other parents with similar experiences helpful, to the extent that they would like to give their support to other parents with GS newborns in the future.

*“What I would say to her is to be patient and strong*, *and ignore those who discourage her*, *be patient and let things into God*’*s and doctor*’*s hands*.*” Mother of a one year and five*-*month*-*old male toddler*.

*“First of all*, *they [expecting mothers] should give birth at the hospital*, *because when you give birth to a child with a problem*, *the doctors check him*, *and when they find the problem complicated*, *they transfer him to a competent hospital*.*” Mother of a four*-*month*-*old male infant*.

Parents found the support they received from different sources was generally positive. Religion, faith, and support from other parents having similar experiences were all important.

### Theme 5: The impact of GS extended beyond the hospital

When the babies were discharged alive from the hospital after surviving the surgical repair was an important relief for the parents. Many were relieved that their children were doing well. They were eating well, gaining weight, and being playful.

*“We were happy*, *the people who went back home with their kids were happy*….*” Mother of a three*-*and*-*half*-*month*-*old male infant*.

However, that did not mark the end of the challenges faced by the parents due to the condition. The biggest concern expressed by many parents was, naturally, about the uncertainty of their babies’ health. One mother who lost her child expressed that she thought her baby was doing relatively well before the baby’s death. The death was very unexpected.

*“He had no problem*, *and I had hoped for his recovery*… *He had not suffered from any disease like the way it happens with other children*… *I woke up and when I was going to clothe him*, *I realized that he had passed away*.*” Mother of a two*-*month*-*old male infant*.

To keep their children healthy, they had to diligently perform numerous tasks—carrying out special feeding practices, maintaining the babies’ cleanliness, attending follow-up appointments either at CHUK or a nearby healthcare facility. All these tasks caused them stress.

*“And before I went to see him [my child]*, *I had to wash my hands and keep the hygiene to protect him from infections*. *I tried to eat and drink because I was allowed to breastfeed him*. *So*, *I maintained cleanliness at home*….*” Mother of a four*-*month*-*old male infant*.

*“*… *The appointment was at CHUK*. *They gave me an appointment and I went there*. *After seeing the doctor*, *he said*, *“I can notice the child*’*s growth*, *he has increased (doubled) in weight*. *I can see that you are doing well*, *I cannot give you any other appointment*, *but in case there is a change*, *you can reach out to your hospital and they can call us*. *But with regards to this sickness*, *I can see that he has no problem*. *Go home and keep breastfeeding him well and eat well*.*” Mother of a one year and four*-*month*-*old female toddler*.

Providing numerous tasks in caring for their babies was not the only challenge the parents faced after leaving the hospital. Integrating into the community with the newborn was not necessarily easy. While some parents received support from the communities, some also expressed they were blamed and shunned by society. The negative societal reaction to GS resulted in the parents’ reservation and denial of the condition.

*“*… *people around the corners were discouraging me by saying “that this baby*, *that thing*, *will never make it*.*” Also*, *there were other people who used to say*, *“even if that child survives*, *he won*’*t be complete*.*”* Mother of a one year and five-month-old male toddler.

*“*… *the neighbors were like*, *“Ahaa*! *Why would a grown*-*up person want to bear another child*? *Let her face the consequences*.*” So*, *when they see me taking the child for treatment*, *they say*, *“let her feel it since she is the root cause*.*” Mother of a two*-*and*-*half*-*month*-*old male infant*.

Naturally, the financial burden was further worsened when parents had to take care of a GS baby at home.

*“I raise him [my child] normally*, *as I had planned to raise him when I was pregnant*. *Even if I faced challenges and ran out of money*… *[Before going to CHUK] I lived beside the street and sold tomatoes and onions*… *So*, *when I got out of the hospital and went back home*, *I could not even find five thousand Rwandan Francs to keep doing my small business*. *I could no longer sell tomatoes and onions*. *I did nothing*…*” Mother of a one year and five*-*month*-*old male toddler*.

Being discharged from the hospital was a joyful moment for the parents. However, the combination of daily tasks of taking care of the babies, as well as reintegrating into the community put a physical, emotional, and financial burden on them.

## Discussion

Parents of children born with GS experienced a wide range of challenges and emotions that spanned across time from when their babies were born to post-hospital care at home and within their community. This study found that the five themes used to describe the lived experiences of the parents were as follows; GS diagnosis had a significant emotional impact on the parents, parents were content with the life-saving medical care provided for their children despite some dissatisfaction with delay and shortage of medications, GS care was accompanied by financial challenges, support systems, and religious faith were important coping mechanisms, and the impact of GS extended beyond the hospital.

The anticipation of the coming of a healthy baby is usually what the expecting parents await [8]. From purchasing baby clothes, sharing the news of their pregnancy to preparing themselves for parenthood, parents generally have high positive expectations. When these expectations are replaced with a different reality, it is understandable for the parents to experience a barrage of emotions [17]. After learning about their babies having GS, our respondents described they were shocked, sad, fearful, anxious, guilty, and hopeless. The situation was exacerbated by the fact that they had no prior knowledge of their children having the condition. These emotions, however, are not unique to GS. Studies have found the lack of pre-knowledge of the existence of congenital disorders and the loss of the ‘healthy child’ image often caused parental negative emotional reactions [8].

GS could be detected before birth through antenatal ultrasound [5]. However, none of our respondents were informed about the condition. On the contrary, those who had attended ANC were reassured that their babies were normal. Without any prior knowledge, they were not only shocked when their babies were born but also not prepared for the needed surgical care. None of them were delivered at CHUK—the only place where pediatric surgical care is available. This had inevitably increased the risk of mortality—as suggested by a study in Uganda. The study found neonates with surgical conditions who were born outside a surgical center had increased mortality [18]. While early antenatal diagnosis alone does not guarantee neonatal survival, it certainly could at least enable the arrangement of delivery at a pediatric surgery center where immediate help could be provided to avoid clinical deterioration due to transfer [19].

None of the diagnostic ultrasonography during our respondents’ ANC visit detected the condition, bringing into question the quality of the ultrasound services. The results of our study highlighted the disparity in health care services between HIC and LICs. A recent study showed that in high-income countries, most congenital abdominal conditions, including GS, could be accurately diagnosed antenatally, while only a few in LMICs [19]. Whether such a gap was rooted in the lack of accurate diagnostic equipment or the shortage of trained service providers, further study is needed in order to strengthen the ANC services in LMICs.

CHUK currently is the only pediatric surgical center in the country. Respondents mentioned frustration and anxiety in the delay of initiation of treatment due to long transportation time. Studies have shown such referral delay was common in the African region [20], causing major challenges in the delivery of neonatal surgical services and potential poorer outcomes [21, 22]. Respondents also mentioned there was a shortage of skilled professionals at the NICU. Such critical shortages and lack of integration of nurses in pediatric surgery programs are common in Africa [23, 24]. There is a need to train more nurses in surgical care, specifically in pediatric surgical care [25]. In addition, the lack of needed medications and lab tests was common in our study as well as in many sub-Saharan African countries [26]. Expanding pediatric surgical services to more than one hospital, with sound infrastructural and supply chain support can improve referral efficiency, reduce travel time, and the financial burden on the families.

Similar to other studies, our respondents were appreciative and had generally positive experiences with the healthcare services their children had received [27]. The health care providers initiated the treatment immediately and contributed to their children’s survival. However, they still had to cope with the situation.

Religious faith or spirituality was commonly used by parents as a coping mechanism. Studies have shown when facing the uncertainty of a child’s survival, religion or spirituality is often a positive enhancer in difficult times [28]. Our respondents found their faith provided them comfort and had kept their mental health in a state of balance. Other support from their spouses, their community, health care professionals, other mothers at the NICU, volunteers, and their workplace, were also crucial. The support can be both emotional and practical, such as receiving money, food, or other consumables. Studies have shown parents or caregivers who received strong support had better acceptance and understanding of their baby’s condition [29].

One key support mentioned by our respondents was the help they had received from other parents who were going through the same challenges. Previous studies have shown that support from these kinds of “expert parents” was very beneficial [30, 31]. Many of our respondents mentioned that they would be willing to give advice and encouragement to other mothers about their own GS journey.

Hospitals, apart from providing strong surgical services, should not overlook the importance of these support systems and should intentionally incorporate religious faith, spirituality, and “expert parents” support in alleviating the emotional burden parents face.

This study indicated that finance was a major challenge throughout their GS journey. Multiple studies have identified financial challenges as one of the prominent factors limiting health service utilization, particularly in LMICs [32]. All our participants were members of the Community Based Health Insurance (CBHI) in Rwanda. The CBHI in Rwanda aims to overcome financial barriers to medical care access [33]. However, our respondents mentioned they had out-of-pocket medical expenses that were not covered by the insurance, such as medications and laboratory tests; as well as non-medical expenses including purchasing food, cleaning supplies associated with the care for their children. Many studies in LMICs have found parents were facing similar financial challenges due to their children having medical conditions [34, 35]. Similar to many studies in LMIC [35], our respondents had taken loans or even sold their properties in order to pay for the health services. Financial risk protection is a core part of universal health coverage, and the need for adequate financial protection in the surgical arena has been widely stipulated by global health authorities [36].

Some of our respondents mentioned that they were shunned and blamed by their neighbors, or even their family members for the condition. In many African countries, the existence of congenital conditions was also met with a mix of personal, social, cultural, and supernatural beliefs [37], there is a need to provide sensitization to society in order to bring understanding and reduce stigma related to congenital conditions.

Being discharged marked the transition of care from the hospital setting to home-based [29]. Being able to do so also marked the sense of normalcy and enhanced the desired parent-child bonding [8]. However, this transition was also fraught with uncertainty, as parents now had to face some known and many unknown challenges—wound care, hospital follow-ups, interaction with other children or siblings; without the presence of healthcare providers [8, 29, 38]. Providing parents with clear instructions and realistic expectations upon discharge could help reduce their anxiety and uncertainties.

### Strengths and limitations

This study was the first in Rwanda to explore the parents’ experiences with babies born with GS. However, the results should be viewed in light of some limitations. This study only collected data from parents whose children were discharged alive from the hospital, their responses could potentially be positively biased. The experiences of those who did not survive the hospital stay were not captured. Although in the recruitment process, our data collector explicitly explained to them that the study was not conducted by the hospital, we could not guarantee the participants did not assume such association and thus affected their responses. The care experience of pre-referral to CHUK was not included in this study. Lastly, we could not eliminate the potential recall bias.

## Conclusion

Understanding the experiences of parents as they cope with providing care for their children with GS would help guide future interventions to advance the quality of health services and improve the survivability of GS in Rwanda. The stress and challenges experienced by the parents began at the moment the babies were born with the condition without any prior information or preparation. And the burden extended beyond the duration of the hospital stay. The parents were generally grateful for the care they had received from the healthcare providers. Their religious and spiritual faith, support from other parents with similar experiences, and the recovery of their children had provided positivity to the situation. Some, nevertheless, had experienced a lack of support from the family and community, mostly due to misconceptions about GS, or congenital conditions in general. Their financial burden was substantial and that continued to affect them even after being discharged from the hospital.

Better antenatal screening can help early identification of such congenital conditions, and in turn, guide their birth preparedness. Community awareness to dispel the misconceptions about GS, or congenital conditions in general, is needed. Hospitals should consider providing faith-based or peer-parent support to help alleviate the parents’ emotional and psychological burden. Future studies on the experience of parents whose children did not survive the condition could provide more insights on how to improve the quality of GS services.

## Supporting information

S1 Text (DOCX)

S1 Data Data files. (ZIP)
