# Supplementary material for: Exploring the lived experiences of parents caring for infants with gastroschisis in Rwanda: The untold story
Source: PLOS Glob Public Health. 2022 Jun 15;2(6):e0000439. doi: 10.1371/journal.pgph.0000439 (PMC10021215; doi:10.1371/journal.pgph.0000439)
Supplement: S1 Text — (DOCX) [file pgph.0000439.s001.docx]

## S1 TEXT: IN-DEPTH INTERVIEW GUIDE (English)

Thank you for the opportunity to speak with you. The goal of this interview is to understand your experience concerning the hospital care your child received for GS and your experience caring for him/her now. We intend to use the information to help CHUK hospital improve services, but we will never share your name or any identifying information with anyone.

Do you have any questions before we begin? Thank you. Let us get started.

- - - 1. I would like to begin with your experience at CHUK. In ____ of 2020, you and your child were in the hospital for GS care. Is that right? Can you please describe what happened during your time in the hospital?

1. How did you feel when you found out your child had GS?
2. What did your doctors tell you about the condition at that time?
3. What were the challenges you faced during your hospital stay?
4. Following your hospital stay, you and your child went home. What was the plan regarding your child’s care after discharge and how did you follow through (follow up, when, where)?
5. What did you think about the health services provided to your child?
6. Was there any emergency that made you seek healthcare? Please explain.
7. To expand some more on that, can you please tell me what was it like being at home with ___?
8. Can you share the main difficulties (financial, work status), psychological, physical) you faced when caring for your child? (compared to your other children or your preconceived expectation of raising a child)
9. Can you share with me how this has affected your social life (family relationships, marriage, community)?
10. *Thank you so much for sharing your experience with me. How is your child doing now?
11. Health condition
12. Feeding habits (breast Vs formula)
13. Growth

*If the child has passed away: I am sorry to hear that, would you be willing to talk about your experience with me?

1. Can you please tell me what happened during that time?
2. When?
3. Did you take your child to the hospital?
4. Can you please tell me something you wish you had been told about your child’s condition and the care needed initially?
   1. What will you tell a mother if they had a baby like yours?
5. Is there anything else you would like to add?

## ANNEX 6: IN-DEPTH INTERVIEW GUIDE (Kinyarwanda)

Wakoze kwemera ko tuvugana namwe. Intego y’iribazwa nukugirango to menya uburyo umwana wawe yitwaweho kwa muganga ndetse nuburyo wowe wamwitayeho. Turashaka gukoresha ayamakuru kugirango dufashe ibitaro bya CHUK kugira impinduka muri serivise batanga, ariko ntago tuzatangaza amazina yawe or umwirondoro wawe kumuntu uwo ariwe wese.

Hariki ikibazo waba ufite mbere y’uko dutangira?

1. Ndagirango ntagirire kubihe wagiriye muri CHUK. tariki ya ya 2020, wowe n’umwana wawe mwari mwagiye . Byaba aribyo? wambwire mugambo arambuye ibyabaye cyino gihe mwari mubitaro?
   1. Wabyabakiriye ute igihe wasangaga umwana wawe arwaye GS
   2. Niki abaganga bakubwiye kubyerekeye iyo ndwara muri ako kanya?
2. Nyuma yuko wowe n’umwana wawe musezerewe kwa muganga. Niyihe gahunda baguhaye y’uburyo uzajya wita kumwana wawe kandi nigute wakikurikiye (hehe, ryari)?
   1. Niki Utekereza kubufasha bw’ubuzima cg ubuvuzi bwahawe umwana wawe?
   2. Haba hari ubutabazi bwihuse bwatumye ushaka ko ubuzima bw’umwana bwitabwaho? Dusobanurire byimbitse.
3. Dukomereje kuricyo twari tukubajije, wambwira uburyo byari bimeze kuba murugo hamwe ?
   1. Ushobora kudusangiza ibintu byagukomereye( amikoro, akazi ukora, guhungabana umuiterereze, cg kumubiri) wahuye nabyo mugihe witaga kumwana wawe?(ugereranyije nabandi bana wawe cg uko wari witeze kuzamurera mugihe warugitwite)
   2. Ushobora kudusangiza uburyo byahinduye imibereho yawe mbonezamubona?
4. *Wakoze cyane kusangiza ubu buzima bawe. Ubu umwana amaze ate?
   1. Ubuzima bwe
   2. Imirire ye (kwonka ndetse kwirisha
   3. imikurire ye

* Nimba umwana wawe yarapfuye, wihangane, waba wifuza kusangiza cg wemera kusangiza ubuhamya nanjye?

1. Ushobora kusangiza uko byagenze muri icyo gihe?
2. Ryari?
3. Watwaye umwana kwamuganga?

5. Ushobora kumbwira ikintu wifuzanga kuba waramenye kubyerekeye ubuzima bw’umwana wawe cg ukwitabwaho kwari gukenewe?

1. Niki wabwira umubyeyi aramutse afite umwana ufite ikibazo nk’icyuwawe?

6. Hari ikindi wifuza ko kwongeraho?
