# Supplementary material for: Exploring the lived experiences of parents caring for infants with gastroschisis in Rwanda: The untold story
Source: PLOS Glob Public Health. 2022 Jun 15;2(6):e0000439. doi: 10.1371/journal.pgph.0000439 (PMC10021215; doi:10.1371/journal.pgph.0000439)
Supplement: S1 Data — (ZIP) [file pgph.0000439.s002.zip › S1_Data/S1_Text.docx]

**BB 1 ENGLISH TRANSCRIPT.**

**MODE: Thank you for accepting to have this conversation with us, the objective of this interview is to find out how your child was taken care of at the hospital, and how you also took care of him. We would like to use this information to help the CHUK Hospital in making changes in their service delivery, but we won’t reveal your names or your identity to anyone, right?**

W1: Yes

**MODE: Do you have any question before we start?**

W1: No problem

**MODE: Speak louder because if you don’t, this recorder won’t catch your voice properly and it will be hard for me to hear what you said. Feel free and don’t be scared, right? These are just usual questions about your life when you were at the hospital, after leaving at the hospital, and nothing is hard, right? If you want, you may also respond.**

M1: Or I may be the one to respond.

**MODE: What?**

M1: Let me be the one to respond.

**MODE: Were you with the child at the hospital?**

M1: I was the one taking care of him.

W1: He was the one taking care of him, I had a problem here, and he was the one in charge.

**MODE: Ooh, you were the one with the child during his sickness?**

W1: Yes, he was the one taking care of him.

**MODE: Okay, you will both respond to me, right?**

W1: Yeah

**MODE: When I ask, the one who feels that he/she knows may respond, right?**

W1: Yeah

**MODE: When was your child admitted to the hospital?**

M1: He went to Kigali after we left Ruhengeri on 24^th^ November, in that night we reached at RWASEC.

**MODE: November, last year?**

M1: Yes, that’s when we reached at CHUK (Kigali)

**MODE: Raise your voice, and speak louder like the way I am doing**

M1: We departed and arrived there on 25^th.^

**MODE: Hmm**

M1: We arrived and he was immediately hospitalized, and they started treating him.

**MODE: What was the span between his birth and arrival at the Hospital?**

M1: He was born here in Ruhengeri.

**MODE: On which date?**

M1: 24^th^, it was around 11 or 12 p.m., and you understand that the following day we left for Kigali. He was born in Ruhengeri, and immediately transferred to Kigali.

**MODE: Hmm**

M1: Yeah

**MODE: For how long did you stay at CHUK?**

M1: We left there, was it on Christmas?

W1: We left there 2 days before Christmas.

**MODE: You left the place on 23^rd^ December.**

M1: We left there on 23^rd^ December.

**MODE: So, it was almost a month?**

M1: Yes, a month

**MODE: How old is your child?**

W1: 7 months old

**MODE: Okay, I can see. He is a boy or a girl?**

W1: A boy

**MODE: Okay, now I would like to start with the moments you had at CHUK from 25^th^ in 2020, you and your child were there, right?**

W1: True

M1: That’s where we were.

**MODE: Yes, can you tell me in details what happened when you were at the hospital? In that period of one month, right? When you reached there, tell me your life trajectory.**

M1: It wasn't that hard because you see when you get to Kigali, one didn't know the place. We got there and they welcomed us, they hospitalized him. When you get there, you realize that kids born in that condition have a place where they put them first. They take care of them in a certain place. We spent 2 days there, when the 2 days were over, they took us to the pediatrics, and we relocated there. So they started taking care of me, but for it to go well, you, the person with the patient, were the one who played the biggest role because you were the one in charge of his cleanliness, who took care of him, looked after him, changed him, plenty of things indeed, and the doctors were ever there for your child.

**MODE: What do you mean by changing him?**

M1: You see how he is, with no clothes, on a bed, you know the place and you see how it is.

**MODE: Yes**

M1: He wore no clothes, and you know that they cover the whole part of the belly. It is to put pampers on him to ensure that he doesn’t dirty his surroundings.

W1: Or we may remove any dirty things around him.

M1: You see that since he doesn’t breastfeed, he is fed via the serum. When he arrives at the pediatrics, he doesn’t breastfeed immediately, he is just fed by a serum. He starts from the quantity of 4 on breastmilk, and after some minutes, let’s say you give him at 8:00 or 9:00 and leave, there is a probe that they place in his nose for feeding him, and there is a syringe that they give you, so you place it inside and draw the content, and you place it in another proper syringe that they give you, you place it inside and draw the content. You place it there and 2 hours later you give him the breastmilk of 5. As he progresses, if you had given him 5 and he takes it well, and you give it to him at 12:00 every after 2 hours, it increases the following day. The doctor says, “we have been giving him 5, today we are giving him 7.” If he tolerates it, they increase it like that till the child reaches 60 and he starts breastfeeding. You might give him and when it reaches 40, he vomits and you put him back to 5, or they also stop him from taking it. And a few days later, they tell you to start from 2 or 3, but when they tell you to give him 5, you give him a little quantity to avoid consequences. There is a time you give it to him in huge quantities and he vomits, which worsens the situation and he gets critically ill.

**MODE: You give him 5 for the whole day?**

M1: Yes

**MODE: Just one day…**

M1: The day when they came to visit, in the morning they would come at about eight o'clock and say, "If he were to take, this day we are giving him five, give it to him all this day." When they come back the following day since they come every day, they give you another schedule and they say maybe put him at 7. The next day when they come, they ask you if he is tolerating and not vomiting, then they put you at 10.

**MODE: Of breastmilk?**

M1: Yes, of breastmilk. You draw via the syringe. They first fetch it in the baby bottle and you also draw it from the baby bottle, and you place the syringe inside it. It has numbers on it, and you check how it is, and you give to him, and it advances like that. There comes a time and you have hope and say, “my child is recovering.” When it reaches at 60 while you were feeding properly, you give to him and he vomits, you do it once more and he vomits.

**MODE: Hmm**

M1: The doctor tells you to stop. By stopping, we first give him through the serum and see if we can do it again tomorrow like that. It depends on the time when it gets right. When you see that the child has reached 60, he starts breastfeeding. But before they breastfeed him, they first give him the baby bottle to learn how to suck. When he has coped with 60, the doctor tells you to feed him with the baby bottle, and the next day he breastfeeds. Then it ends like that.

**MODE: Hmm**

M1: Yeah

**MODE: The 50 and 60 that you mentioned, do you have a scale for measuring it?**

M1: It is a syringe; don’t you know a syringe?

**MODE: Yes, I know it**

M1: The one for injecting. They take out the syringe, you start with one of 5, it's there. When they reach 10, it is also there, you just throw the first one. So it goes up till 60, which is the biggest one.

**MODE: Thank you. How did you react when you saw that you gave birth to a child with external intestines?**

M1: It wouldn’t be easy explaining it. We are here, he was born here in Ruhengeri. I said, “you can see how it is, this is a catastrophe. Give me my child and I take him home, there is nothing else.” The doctors said, “No, do not worry because he will recover. There are others who were also born in the same condition that recovered,” then they added, “we are transferring you to Kigali for treatment.”

**MODE: Hmm**

M1: Then we got in the car and left, but I went there with no hope. Even leaving there was because of God, because bringing him out that place was luck.

**MODE: And for you, how did you feel when they told you that the child had…**

M1: No, it is understandable since I was the one with her, I was checking on her every minute, when one is doing well, you see it, and when things are not going well, you also see it.

**MODE: How did you feel, as a mother?**

W1: I said that since I left the delivery room safe, even though the child would probably not make it, God would give us another one, but at least I stay. I accepted it, and when I got back to my senses, I asked the doctors who were there, "where am I? " They responded, "it's at CHUK." I told them, “Show me the outcome." They went to show me the baby, I found him there, looked at him and said it is fine since I saw the outcome. I waited for what God would do that we may return safe. Even if we do not return alive, then that will be God's will.

**MODE: You just gave birth and didn’t know the outcome till you reached in Kigali?**

W1: Yeah

**MODE: What did they say was the root cause of that sickness? You were there.**

M1: They also don’t know.

**MODE: Hmm**

M1: They also didn't give us enough notice, they just told us that it was a new disease. You know when you get there and find your friends sick. it's a disease, though they didn't tell us about it. They said that it was a normal disease. There is a time when one heals, and there is a time when it is over, and one leaves knowing it ended.

**MODE: When you reached at CHUK, what did the doctor tell you about his condition?**

M1: You mean the doctor?

**MODE: Yes**

M1: The first thing is to have hope, though it is uncertain, what he did was to tell me, “Pray, he will recover. There are those who recover, and if it doesn’t work, then we will have no other choice. We do our part and God also does His part.” That was it.

**MODE: Can you tell me in details the challenges you faced when you were in Kigali?**

M1: In Kigali?

**MODE: Yes**

M1: In Kigali there is no problem, you also know things of Kigali, it is a problem when you don’t have money. However, money does not solve everything. But the first success was seeing that people living in Kigali were financially on the same level. I left the place and I can tell you about it since I was there for a month, things in Kigali are difficult when you have no money. But for things to work out perfectly with these children, the first thing is cleanliness. When you don’t maintain his cleanliness, you face tragedies.

**MODE: Hmm**

M1: Another thing about the cleanliness of a baby is that every minute you change his pampers, and there are even times when one has no money. And you also have to bathe the child, they bathe them with hot water. Sometimes you don’t have a water heating device, and buying it requires funds, which becomes a challenge.

**MODE: How would you explain your financial situation? Was it difficult? Was it easy?**

M1: One had no support while going to Kigali, one is coming from Ruhengeri and here in Ruhengeri, we work for wages. When you leave your home with enough funds, it goes well and God moves ahead of you. You live in Kigali when you have money. When you go there poor, there are times when God saves you from there. But you can get there anytime and get support and find someone who says, " come to see me and get I'll give you pampers," but such chances are rare.

**MODE: Hmm**

M1: You can miss those chances and it ends like that, and the child passes away because of financial challenges since if you made calculations of those used minute by minute in a day, you can use 10 pampers. You find that he has defecated and you wipe him, you bathe him in the morning, at noon, in the evening, and water is also a challenge. The next day you find that things are very difficult. You have no money, you see that photocopying costs you a lot, carrying out tests every time, and you find it problematic.

**MODE: Which insurance did you have? How did you pay for the bills?**

M1: The bonus we had was that we were in the countryside. So that’s why we brought him home because there were no heavy expenses. The main thing for us was to photocopy documents and get him some cleaning supplies. Another problem that could occur, there were times when there was a shortage of medicine, so you go out and look for it, and you have no money, do you think you can go there? You tell them that you didn't find them and it ends up there pending, and life goes on. Everyone has their own plans in Kigali, and God is involved, and He says, "Let it be so, and He helps you in that way."

**MODE: In the society where you live (in your neighborhood), how did they react to the news?**

M1: As in here? You can even obtain the information and I deny it. Would you come and state that it was like this?

**MODE: Hmm**

M1: I would tell you that those are lies, those things are unusual dear mom.

**MODE: They never knew about it?**

M1: No, even if you knew and asked me about it, I would deny it.

**MODE: Why would you deny it?**

M1: No, I would deny, stating that one was born with external intestines, do you think that it is news to share?

**MODE: It is not news to share, but you can’t also hide it**

M1: No, that is a secret of the doctors. You may tell it to someone and they refuse to accept it, unless you show them the papers proving his treatment. You may tell it to someone and they refuse to accept it.

**MODE: Did you discuss it with your friends?**

W1: Those who helped us when we were in Kigali, and we could tell them the problems that we faced.

**MODE: How would they receive it?**

M1: They know the problem we faced, they saw him and were shocked. It was a catastrophe, could you imagine giving birth to a child whose intestines move from here to there?

**MODE: Touching downwards?**

W1: Yeah

M1: In Kigali, they tie them when he is lying on the bed and it stretches about 2 meters on the extreme end, the doctor comforts you that it will recover, and you see it lower down in the bag every day. So here's where the challenge lies, when it's hanging like that, there is no problem. There is no problem at all, the baby is playful, the plans go well. The time it gets messy is when it's fixed back in the belly. When they cover it up for more than 3 months, the baby is covered, and he swells, you get?

**MODE: Yes**

M1: I can show you photos of how he was and you can compare them to how he is. You can multiply the size of this hand by 4, the head is so big, and you think that it is an adult, the thighs swell to this size, and the face! It's a pity. The child swells and gets poor sight, the eye is overgrown, and the whole body is swollen. That was what they were going through.

**MODE: So, they recover after how long? When does the swelling stop or when do they get back to normal?**

M1: The body has white blood cells, and the recovery from swelling is by breastmilk, it is the one that reshapes the body. When he takes it and vomits it, the time of recovery increases. When they give him breastmilk and he tolerates it, he starts regaining shape, and it’s the one that helps. That’s how it goes.

**MODE: During that time, at least you could see him, did you also see him when you gave birth to him?**

W1: I was looking at him as I was getting better.

**MODE: How was it? How were you feeling?**

M1: It is understandable that when you reach in Kigali there are two things involved, it is either life or death. We got there when there were many people, when I got there on the first round, putting aside those discharged and those I found there. The people I found there; I left the place when they were still there apart from 2 children. Those who went there are numerous.

**MODE: Hmm**

M1: Indeed

**MODE: Hmm**

M1: You can’t get there and say that you will return safely/recover. It is God who gets involved.

**MODE: Okay, you stayed with the sick child, a time came and you were discharged. After discharged from the hospital with your child, which plan did they give you of how you will take care of your child?**

M1: Cleanliness, cleanliness is the first thing.

**MODE: What did they tell you? Tell me in details everything that they told you.**

M1: After getting discharged, we left when the hole had closed, there was still a cover. They told us that even the beds are few, and so on and so forth. You see that once the baby recovers, they discharge you because his stay can result in sickness, and he suffers from other diseases. He said, "Go since you live near the hospital, this cover after a week or five days, like twice a week, go to the doctor and change it.

**MODE: Which hospital?**

M1: The nearest hospital

**MODE: Okay, whichever hospital**

M1: Yeah, they go and change to put a new cover. But there are times when you bring him home like that because the rural life and the life in Kigali are different. They tell you to go and you take the child to the countryside, and when you get there, life changes because in Kigali he is clean, safe in a quiet, warm, comfortable place. When you get to the village, he falls sick again, but he does not suffer from other diseases. He becomes ill due to the wound and you find that he has a fever and shortness of breath, and you return to the hospital. Since we got here and went back to the hospital in Ruhengeri, they were about to transfer us back to Kigali. But they treated it from there and it healed, but there is an obligation to keep it clean, and go to change the covers, and do it for 3 or 4 days. When it disappears, it ends and the child returns to normal life like other children.

**MODE: Apart from telling you about wound covering, was there anything else that they told you?**

M1: Other things?

**MODE: Yeah, of taking care of him. Other ways of taking care of him.**

M1: Since they are born with little weight because of that issue, when you have the means, you go to buy things that aid in weight gain and give them to him. How else could it go? And he starts life like anyone else and gets tough.

**MODE: HHHH, what do you think about the medical assistance/treatment that your child received when he was at CHUK? What can you say about it?**

M1: Us?

**MODE: Yeah**

M1: What they did was great, they tried their best. They took care of us and there were no problems in Kigali. The only challenge is to go there when you have no money, there would be no other problem. And for the services, they provide them and there is no problem.

**MODE: Give me an example of a service that they gave you and you felt satisfied?**

M1: In Kigali?

**MODE: Yeah**

M1: When you take a child in that condition, and they treat him, and he recovers, but here is the challenge, there were people living there, what I am telling you, I was the one with the baby till he recovered.

**MODE: Hmm**

M1: There were some students who came in as nurses and not doctors, we can call them interns in the field of medicine. There are times when a doctor says something and they don’t hear, and they do the contrary, since they are the ones that give the medicine.

**MODE: Hmm**

M1: They are the ones that give them medicine, and the doctor comes to check on the baby and tell them the medicine and dose amount to give, and then he leaves. I would find myself quarrelling with these guys. There were times when they gave him the wrong medication. The doctor would say, how did you give him this and that, you mixed it up badly, and you found out that it had a negative effect on the baby. It may even result in his passing away because they didn’t understand the instructions.

**MODE: Hmm**

M1: Or you find them piercing the child a lot because they are searching for the veins. Since they haven’t known it yet, you find them piercing the whole head, the legs, the arms, and disfigure the child’s beauty.

**MODE: Hmm, okay.**

M1: And many passed away because of that. If the doctor has told him/her to mix this medicine with that one, mix 5 doses like this, and you exceed what you were told or put in little quantity, it causes you deep sadness.

**MODE: Apart from that, what good things did you find at…**

M1: The good things are many indeed.

**MODE: Yes, tell me those many things.**

M1: In Kigali, apart from stating that this child is not served food, but in Kigali when you have a patient, they bring porridge in the morning and you eat it.

**MODE: Who?**

M1: I don’t know, I saw people dressed in white coats that brought porridge and bread, they also brought food at noon and you ate, and also in the evening.

**MODE: So, you never bought food?**

M1: No, unless you are buying for the mother, and she doesn’t manage, however, oh my God, you woke up and were given porridge, bread, and the one who wanted tea was also served, and the one who also managed that which is processed by a machine.

**MODE: Milk**

M1: Milk and everything else. They could give and you drink, and at lunch you eat and get porridge as well, and in the evening, it was the same, while for the drinks, it was on you, but there was no hunger there. The food that you can buy is for the mother, that is where you try and buy for her, but the livelihood there was not a challenge. There was food for people who took care of the patients, I only saw this thing in that hospital. Otherwise, getting food and drinks is the most essential thing. The place is like home, but the challenge is one of finances, but they also see how they can assist you and it goes well.

**MODE: Was there an emergent issue that made you seek immediate medical intervention for your child? Maybe you took him to this place, and he falls sick requiring you to take him to the hospital immediately?**

M1: Medical intervention?

**MODE: Immediate medical intervention, I am not confirming but asking. After bringing him here, did he ever suffer to an extent that it required taking him to the hospital immediately?**

M1: Yes, he fell sick.

**MODE: How did it go?**

M1: He fell sick and had shortness of breath.

W1: He had 15 minutes left to live.

M1: He had fever, we went to the nearest hospital, they refused to give us an ambulance and obliged us to go by foot. If we hadn’t found the ambulance of the district hospital, that was during COVID. It was around 10:00, they saw us walking as we carried the child, and they are the ones that rescued us. If it hadn’t been that help, he would have passed away.

**MODE: Hmm**

M1: Since it is only a wound, there are times when other diseases from germs arise and we take him there.

**MODE: How did it go? How did it start?**

M1: It started when he wasn’t breathing well and he had fever, we were living far from the main road. We reached at the hospital, they tested him and stated that we have to take him to the provincial hospital. And they said, there is no ambulance, go there by foot. Just imagine moving a journey of 5-6 km.

**MODE: Where were you? Was it here at Nyamagumba?**

M1: No, we lived in that northern corner over there, in Kinama sector. We went to Bukamaka at 10:00. He had breathing issues and they said that they can’t manage him. They said, “bring the money so that we may write a transfer notice,” and we paid. Then they said, go to the provincial hospital. We went, and the journey by foot lasts for 2 hours. We went, and on our way, the ambulance rescued us, when we reached there, the measure was on 40, and when it reaches on 40, you are at the brink of passing away.

**MODE: You mean the temperature?**

M1: No, there is a way they tested basing on how he was breathing. When he was healthy back in Kigali, the measure was 100. There was a device that they brought for testing and put it on the arm, and when he is on 100, I don’t know the situation is. So when it starts dropping, it means that she is heading in a dangerous zone. He had reached 40 and was about to die but they saved us and took us there, and they immediately treated him. They put him on oxygen support and regained his life.

W1: They saw that he had refused to breastfeed.

**MODE: So for how long did you stay in the hospital when you went there on that day?**

M1: We spent there 5 days.

W1: We spent there a week.

M1: We spent there a week, we left Kigali and a day later, we went to the hospital.

**MODE: There was just a gap of one day?**

M1: One day passed and we went to hospital.

**MODE: What kind of support did you receive during that week?**

M1: Here in Ruhengeri?

**MODE: Yes**

M1: There was nothing special about that place; they just did things that you could do at home. It was just putting him on oxygen support since their services are not that good; that's what happened. They also injected medicine as usual, and to go past that stage was God. Just imagine that the baby can look at you and get dry when no doctor is appearing. They put him on oxygen support and injected medicine. There was a doctor who came only on Tuesday, he was a doctor from Kigali who came, and on Friday he arrived. For other things, they do what they want, like those in Kigali who pierced the baby without knowing what they are doing. It is God who gets you out of there and nothing else.

**MODE: What did they say was the cause of shortness of breath?**

M1: They said it was caused by the wound which hadn’t healed yet.

W1: We reached at home and put a cloth around that place where air passed, the urines went across that cloth and entered the navel.

**MODE: Yes**

W1: That was the main cause.

**MODE: Ooh, for real!**

W1: Yes, and when it entered, it occupied the breathing area and caused shortness of breath. Then we immediately went to the hospital.

**MODE: Okay, you spent there a week and returned. After returning that time, did he ever have any other problem?**

M1: We returned there on few occasions and it was just normal, because he had recovered and those were diseases that any child suffers from.

**MODE: Like which ones?**

M1: These usual ones, a child can get fever or catch a flu or cough or refuse to breastfeed. The sign showing his sickness is when he refuses to breastfeed.

**MODE: Proceeding on what we were talking about, tell me from that time onwards, he returns healthy, tell me about the experience of living with…, I heard that you call him Paci?**

W1: Yeah

**MODE: How is the experience of living with Paci on a daily basis?**

M1: After recovering, he is a normal child like any other.

**MODE: Hmm**

W1: We take care of him.

M1: The next thing is to take care of him when you have the means, and you provide him with a balanced diet. So he gets back to a normal life and we increase his quantity of food, nothing else.

**MODE: The balanced diet that you…**

M1: A balanced diet, he got 6 months and started eating. I find a balanced diet for him to eat, and when you get milk, you also give it to him, but they forbade us from giving him cow milk. And you know that the milk from supermarkets is expensive, a packet is like 500Rwf and a box is 6,000Rwf. You can’t have that, so we leave it. When you get quality milk, you give it to him, and if you have means, you buy that cup of NIDO, and give him eggs, and cook vegetables for him and other additional things. You also give him porridge. But they said that the cow milk will also be served to him when he turns a year, now the milk is not compatible for him.

**MODE: Is he your first born?**

W1: Yes, he is the first born.

**MODE: Okay, can you tell me the financial barriers that you faced while taking care of him?**

M1: Of course, one has to take care of him, get him a balanced diet even though life is tough. This is also a city like Kigali. If they say you're going to get him a balanced diet, you get him some milk, NIDO, and those other things, eggs, and those are expensive things. When you manage to get one, you give it to him, and when you lose it, you have to calm down. For the porridge it's normal, you get him what he eats and it ends from there.

**MODE: The work that you do in relation to the sickness of Paci or his situation, how did it change?**

M1: You just have to stop working and take care of the child, solve his problem first of all, and get back to work later.

**MODE: Hmm, so you stopped working?**

M1: Work had to stop since you can’t go in Kigali and take care of one and you know that this one was also sick, she also spent a month in the hospital. She wouldn’t go to see the child when she was sick, so I had to stop work and take care of them.

**MODE: How about you, do you work?**

W1: I used to work.

**MODE: By then (before)?**

W1: Yeah

**MODE: Since then, you never went back to work?**

W1: Yeah, I never went back to work.

**MODE: Till now?**

W1: Yeah

**MODE: What losses did it cause you? Or the challenges you both faced as a result of stopping work to take care of your child?**

W1: Work stopped, but there is no problem since we left safe and happy with no problem.

**MODE: Did it affect your mental health?**

M1: Mental health?

**MODE: Yeah**

M1: Yet we came back with a normal person?

**MODE: Hmm**

M1: No, there was no problem. Life is expensive. There was no problem, they just told us that it was sickness, if there are other things involved I don’t know, it is sickness just like one suffers from malaria, that’s what they told us and it is also treated.

**MODE: So what did you find challenging as you link the way you planned to raise him and the reality that you faced, and how you got to raise him? Because before you gave birth to him, you were expecting to give birth to a healthy child, there are certain expectations you had, and then it changed. What was the most challenging in that change that you were expecting to raise your child and the outcome that you saw?**

M1: You understand that if for example, you were expecting a baby to be born, and it all ends from your local small hospital. We call it a small hospital, and you see things coming down to the provincial hospital and you go to Kigali, it's a challenge, and giving birth here like a project. The plans have changed drastically and if a baby is born healthy and spends 3-4 months, they release you and you go back to work. You are going to take care of the baby the whole month seated, and then you take him home and see there is nothing else you can do, and you find it as a big challenge. So losses are inevitable.

**MODE: What did the sickness of Paci change on your relationship?**

M1: The relationship?

**MODE: Hmm**

M1: People didn’t see/comprehend things the same way, can you reverse the occurrence? When someone tells that it is sickness, you take it to be normal, it is sickness just like one may suffer from any other disease.

**MODE: Didn’t this change the relationship that you had with your wife?**

M1: No, there you would be going far, saying that it’s the people from her family who give birth like that, no. It was just an illness and you can see that now he is a healthy boy. Now he weighs 7kg.

**MODE: Currently?**

M1: Yes

W1: Approaching 8kg

**MODE: He was born with which weight?**

M1: 2.400kg

**MODE: So you worked together, this is not to cause a negative change, maybe his sickness might have made your relationship stronger. Did it change anything?**

M1: No, there is no problem. Living in the city requires funds, and whenever you find means, you have to share. He is given whatever is available, and what is not there, you leave it, and God also supplements.

**MODE: I am talking about how your relationship used to be.**

M1: Our relationship has no problem.

**MODE: The conversations continued?**

M1: You have to converse. You know that even one may give birth and the baby immediately dies, don’t you still converse? And would you have your baby saved and say that it is not a blessing?

**MODE: Hmm, okay. Thank you for what you told me. How is Paci doing right now?**

M1: He has no problem and he’s not been falling sick. The disease comes and disappears, he has no problem.

**MODE: Doesn’t he feel pain at times?**

M1: Pain?

**MODE: Hmm**

M1: When the part has merged, there is no other problem, even though you are the only one who knows his secret, there is nothing else. When it has merged, he is normal like other children.

**MODE: Does he ever vomit? There is something you were about to say.**

W1: I was going to say, the challenge we have is that they had given us an appointment, and the lockdown came. We request that they may give us a transfer so that they place him through the scanner and see if he has recovered fully.

**MODE: When were you supposed to return there?**

M1: In Kigali, we were discharged and told to return after a month because the system was like that, and they place him through the scanner to see if the intestines have been fixed properly. The time reached when they had closed.

**MODE: You mean the COVID lockdown?**

M1: Yeah, they restricted movements when we were about to return, and when we went to Ruhengeri, Emmanuel became challenging. If he had given us a transfer, we would have gone there.

**MODE: Who is Emmanuel?**

M1: He is a doctor in touch with Edmond. Edmond knows him. He is a doctor taking care of children in the pediatrics of here in Ruhengeri, he is the one who writes your transfer to Kigali. He refused to write the transfer and said, “I can’t transfer you to Kigali, this child has no problem.”

**MODE: Did he at least check him?**

M1: Check him like how?

**MODE: His belly**

M1: In the belly…

**MODE: To finally conclude that he has no problem**

M1: They don’t have the scanning device for children in Ruhengeri. When a child has a problem, they transfer him/her. He just looked at the outside body and said,” I can see that he is looking good, he has no problem. Go back to your home.”

**MODE: And you wish to return there?**

M1: If they allowed me right now, I would board and get there, I can pay for the transport. Another thing I would like you to see, let me check, I’ll find it in a while. Look at this handwriting of doctors, maybe it’s that one, even though we were discharged, we were to return there in January, I am not seeing the date. The other time we reached at the hospital was 24^th^ November. No, we were supposed to get there in January, on 10^th^.

**MODE: Here it is, 11^th^ January.**

M1: That’s when we were supposed to return. There had been a lockdown, we went to Ruhengeri and he spent there two weeks, and he said that the child was good looking, and it was not necessary for us to go to Kigali. He said we should go back home and that it was necessary.

**MODE: Your wish is to talk to Edmond?**

M1: No, if they accepted. Emmanuel called Edmond. No, he refused to call him, we talked and he said, “you can’t go to Kigali when the child is not sick, go home, the ambulances need to carry many people, go home.” I returned there when there was another doctor, and that was when I saw Edmond’s number, we talked and when he is about to write a transfer, he said, “I can’t send you there when there is no doctor to treat him so that he returns quickly, you can delay there.” I got his number and he said, “talk to him, in case he gets a problem, get back there.” I called him and we talked. That’s when I got his number. Can you just wait and take a person for checking through a scanner when he gets sick?

**MODE: No**

M1: And those who treated and discharged him said, "Come back so we may see if it was fixed well," there are times when it is fixed badly when he's still a child. It is not clearly seen, but once he grows up, they'll have to operate him again." That's what they told us; they wrote it here. They said, "bring him back and we pass him through the scanner and see if his intestines were fixed properly so that it may end from there. But if they accepted right now, I would immediately go there.

**MODE: Didn’t you ask Edmond to let you get back there?**

M1: Whatever you may say when you are still here, if Emmanuel doesn’t approve it, then it is impossible.

**MODE: Didn’t you ask Edmond to talk to Emmanuel?**

M1: They talked since they are ever in touch on a daily basis, whatever you may do, Emmanuel cannot call him. You see that Edmond is also a busy person with a lot of work, he can’t say, “send me that child so that I may check him through the scanner.” He sees the child breastfeeding and says no. If they just wrote it and asked me to transport myself, I would go there. Another challenge would be to transfer you when you are not going to meet a doctor, when the appointment is already over, it is indeed problematic.

**MODE: Okay, remind me tomorrow evening, right?**

M1: Hmm

**MODE: When I get there, I’ll ask for you, right?**

M1: Yeah, if they put him through the scanner, it would be great. And we see the outcome, instead of letting him grow to face other challenges, they would check him and see if it were fixed properly, and then it ends.

**MODE: Does he ever suffer from diarrhea?**

M1: Suffer from diarrhea?

**MODE: Yes**

W1: That was in the days when he was…

**MODE: He was?**

W1: Growing?

**MODE: What?**

W1: Growing teeth

**MODE: Ooh**

W1: That’s when he had issues related to defecating, but he could not vomit. Since they have grown, now he has no problem, he eats and drinks, and defecates well.

**MODE: So, it was not related to that disease?**

W1: Yeah

**MODE: Does he cry frequently?**

W1: He doesn’t cry that much.

**MODE: Does he breastfeed only, or he also eats? Or he takes the milk you were recommended? How is his diet?**

W1: It is all those things, oh my God, now he takes it well, and after eating, I bathe him and he sleeps. When I wake up, I breastfeed him, and he lives well with no problem.

**MODE: What does he eat? He only breastfeeds and doesn’t take anything else?**

W1: No, he breastfeeds, drinks the SOSOMA flour, eats eggs and fruits, potatoes, dodo, and milk.

**MODE: How is his growth?**

W1: When I take him for vaccination and they put him on the weighing scale, you find that his weight is increasing.

**MODE: And the height?**

W1: The height is also increasing.

**MODE: Can you tell me since his birth till now, the changes he has had in his growth and in his height?**

W1: In his growth?

**MODE: His growth, I mean the weight and the height, what was his weight? He has increased by how many kilograms so far? What was his height before? What is his current height?**

W1: I don’t recall his height but when I take him for measuring, they find that his height is increasing.

**MODE: The weight?**

M1: He was born with 2kg and 400g, and now he has 7kg. You can hear that he is growing.

**MODE: He is growing indeed. Is he a happy child, playful, with no problem?**

M1: No problem ever since we left Kigali.

**MODE: Isn’t he a lonely child?**

M1: No, he can be part of others, loneliness can only come when he is grown up and he identifies the way he was born, that would be isolation…

**MODE: No, I meant like when he is very quiet or playful and laughing, how is he?**

M1: No, he has no problem.

W1: He is ever laughing and playing.

M1: No problem, he is doing well.

**MODE: That’s great. Can you tell me what you wish to have known about the health of a child or the care that he needed before his birth? For example you may say, “if I had known this, it would have helped me more.”**

M1: And I know it when he is still in the womb?

**MODE: Whatever you would choose whether in the belly or the way he was treated at CHUK. You also see like when you treat a child and he recovers, after that, basing on the knowledge you are acquiring about the disease, you may say, “had I known this and that, had I known this information before, it would have helped me in a particular way?”**

M1: Before his birth?

**MODE: Whether before his birth, whether at the hospital, whether in the transition of taking him to the hospital, what did you learn that you wish to have known on time?**

M1: No, it is understandable, if you brought a mother from the hospital from the echography, they check and see it before his birth when it is like that, you can’t say that he should be born like that, you can suggest that they make changes to end the problem.

**MODE: So what would you have wished to know?**

M1: They would abort and he leaves instead of…, do you feel that these are things one would live with?

**MODE: Hmm**

M1: No, no, no.

**MODE: Now you wish that if you knew it before, you would have preferred an abortion?**

M1: No we can’t say it right now, but if you knew it before it happened, you would choose that.

**MODE: Hmm**

M1: They would do that.

**MODE: Hmm, for you, what is your say on that?**

W1: Me?

**MODE: Hmm**

W1: A doctor who said it was with another one and he asked, “if they check in the echography and realize it’s like that, what do they do?” He responded, “the immediate assistance we provide is to abort instead of…, and he added, “if the baby had an issue and died in the womb, or the mother also passes away, then we immediately remove the child, and the parent stays.”

**MODE: Hmm, when they see it as the baby is still in the womb**

W1: Hmm

**MODE: How were you feeling when you were pregnant? Were you feeling normal?**

W1: I felt that there was no problem in the womb and that the child was doing well.

**MODE: Playful? And no…**

W1: Playful

**MODE: Didn’t you get sick or…?**

W1: I never got sick.

**MODE: What would they tell you when you went to the hospital?**

W1: I went there and they told me that the child was doing well, they told me that I carried a baby girl, the father (this one) was with me.

M1: Their devices are dysfunctional.

W1: I was with this one, they told him, “Your wife is carrying a girl in her womb”, and the child is fine with no problem.

**MODE: Hmm**

W1: When I gave birth, it was a boy who also had a complication. This one became furious, and said “why would you lie to us?” It was hard for him and the grandmother to accept it, so they first quarreled with the doctors and had a problem with them, but they later accepted it when they came to Kigali.

**MODE: Hmm, what saddened you the most?**

M1: Me?

**MODE: Hmm**

M1: I learned a lesson, the next time I won’t take a child to Ruhengeri. I will go to a private hospital and pay a lot of money, and check through the echography how the baby is doing. Can you imagine someone telling you that you will bear a girl, and you buy clothes for a girl and make other preparations, and at the last minute you give birth to a boy, who is also born with a complication? Yet they had told you that it was a girl, in good health, with no problem. You also understand that challenges are inevitable in that situation.

**MODE: You never felt sick, you were feeling normal just like any other pregnant woman?**

W1: Hmm

M1: There was no problem.

**MODE: So you both went through that life, took him for treatment, and the child later recovered, and he is doing well, as I can see. What would you tell a parent whose child has a problem similar to the one that your child was born with? Each one will state the message for that parent. You may start.**

M1: The parent should pray and consider the cleanliness of the child and leave the rest for doctors. That’s it. Cleanliness is the first priority.

**MODE: Hmm, and you, what would you tell a parent with a child having a similar condition to the one your child was born with?**

W1: If she went to the hospital, passed through the echography, and heard that it is the same situation. I would tell her that if they asked her to abort, she should keep the child and give birth, since I also gave birth to that child and he recovered. You can see that the child is doing well, you should also wait for God will do because God provided him/her as a fetus in your womb, instead of removing it, they should let you deliver it.

**MODE: Anything else? In matters of taking care of him since you’ve been with him, 7 months is a lot. By all means, you can give to the parent more than one piece of advice.**

M1: No, it is just cleanliness and nothing else.

W1: Even if they said that they are discharging me, how would you help your child to grow and attain that level? I would tell her how we took care of him and maintained his cleanliness.

**MODE: Okay, thank you. Our discussion is nearing its end, I asked for the information that I needed to know, now is your time for asking me. It’s not a must to ask, it can even be ideas, suggestions, say anything, and feel free.**

M1: Regarding what?

**MODE: Anything, the conversation we had, say what you want.**

M1: Okay. In general, maybe one would have asked this before, it’s just that the conversation had to come first. You came under which circumstances? Who sent you here?

**MODE: Our team, me and 2 Ethiopian doctors who are studying on this disease, right?**

M1: They want a copy?

**MODE: Hmm?**

M1: They need a copy?

**MODE: A copy of what?**

M1: Of finding out how things went.

**MODE: No, they just discuss with you to find out how it went, to know what they can do to make changes in the service delivery, since you are the ones who spent much time with the children, right?**

M1: Yes

**MODE: So they can’t tell themselves what needs to change without asking the beneficiaries, because, as you said that you didn’t receive proper medical care at Ruhengeri, or in Kigali, you faced this challenge, like the challenge of nurses that you talked about.**

M1: When you get there, tell them that problem so that they may work on it.

**MODE: They might find out that since parents disliked that, nurses will no longer touch on the children requiring skills that they don’t have.**

M1: They are just old women who do not see clearly, who pierce the child and you see blood over that place because they cannot see the vein. Think about that please.

**MODE: Hmm**

M1: And as they cause more wounds, it creates more space for germs, and you find that it’s a problem. The people who cannot identify the veins, and who later go to consult a doctor, who just takes one shot and finds it, and you find that it is a challenge.

**MODE: Hmm**

M1: If they pierce this place and a drop of blood is spilled, and they pierce the other end, they go to arms, and shoulders, in the head, and pierce everywhere, doesn’t it cause blood loss?

**MODE: Hmm**

M1: But they can get an experienced doctor who can work on it, pierce once and you find that things are going well. By the way, greet Jackie for me.

**MODE: Jackie, yes**

M1: She might have given birth now, she is a fat lady.

**MODE: Hmm, Jackie helped you?**

W1: She helped us a lot.

**MODE: Okay**

M1: And these nurses, even though it’s not all of them, one would come and you would see that he/she is ignorant, and he/she is not experienced in what he/she is doing. There are others that do their work very well like Robert and others. For example, there was a time in the night when the serum got off the child, and when you go to wake the sleeping medical practitioner, s/he accuses you of removing it, and she insults you and you end up quarreling. That is also another problem.

**MODE: Hmm**

M1: Or when the serum exhausts, you tell him/her and s/he asks you to wait. And you keep waiting till the next day. Or s/he leaves in the morning, if it finished like some minutes past two, in the morning s/he leaves without replacing it. And they replace it at noon.

**MODE: Hmm**

M1: I tell them and they tell me to wait. I don’t know if the response “wait” should ever be in the health sector.

**MODE: Okay, you? What is your suggestion? Or question? Tell me.**

W1: Me?

**MODE: Yeah**

W1: Jackie, the one he was talking about, it reached a time when the child was sick, and the skin peeling off, and the father getting anxious, and Jackie helped us all night long. She called the doctors who were not around. She said, “there is a sick child here, come and take care of him. He is having some changes with his body.”

**MODE: Hmm**

W1: So she called the doctor, and they came to treat him.

**MODE: Okay, anyone else who has something to say before we close?**

W1: Another thing is that they helped us, I never experienced hunger, no problem.

**MODE: Hmm**

M1: Is Kazungu still in charge of the hospital? Greet him for us as well.

**MODE: Hmm**

M1: If the tall man is also still around. He could come to inspect on them and they could work well, and correct their mistakes, and you would find that there is no problem.

**MODE: Hmm**

M1: He is the one in charge of pediatrics in the General Watch, because that’s where those children are kept.

**MODE: Hmm**

M1: For the things concerning him, he should hear them as I speak about him. Greet him for me.

**MODE: Yes**

M1: And Edmond and Jamal and others as well, I wish you blessings of children.

**MODE: They are many.**

M1: They are many for one to mention and finish the list. Just salute them.

**MODE: Thank you for giving me your time, it was nice. And the way you work together to support each other, may God bless you. This marks the end of our discussion.**

M1: Thank you.

**MODE: Yes.**
