# Supplementary material for: Exploring the lived experiences of parents caring for infants with gastroschisis in Rwanda: The untold story
Source: PLOS Glob Public Health. 2022 Jun 15;2(6):e0000439. doi: 10.1371/journal.pgph.0000439 (PMC10021215; doi:10.1371/journal.pgph.0000439)
Supplement: S1 Data — (ZIP) [file pgph.0000439.s002.zip › S1_Data/S10_Text.docx]

**BB 4 ENGLISH TRANSCRIPT.**

**MODE: Now we can start the discussion, and I can start recording our conversation, right?**

W1: Yes

**MODE: Thank you for having this conversation with us. The purpose of this interview is to find out how your child was taken care of at the hospital and how you took care of your child when he was sick, with a complication of external intestines.**

W1: Yes

**MODE: We want to use this information to help CHUK Hospital improve its services, but we will not disclose your name or profile to anyone. Do you have a question before we start?**

W1: I have no question.

**MODE: Okay. I would like to start with your experience at CHUK when you were at the hospital in 2020, right?**

W1: Yes

**MODE: You and your baby were admitted to the hospital as you needed medical attention for the complication of external intestines. Is that true?**

W1: Yes

**MODE:** **So, can you tell me in detail what happened when you were in the hospital?**

W1: Yes, I can tell you.

**MODE: Okay, tell me.**

W1: When I arrived, they welcomed me, and they placed the baby in an incubator. It was on Tuesday that they fixed the intestines inside the body. On Saturday, I started fetching breastmilk for him. On the following Saturday, they informed me about how to feed him with breastmilk. I started on 5.

**MODE: Sorry for interrupting, you said that you started on 5. What does 5 mean?**

W1: 5 was the measure of the quantity of breastmilk that I was fetching for the baby.

**MODE: Yes**

W1: They increased the quantity to 10, the next day they set it at 15, the following day they set it at 20. It increased like that till it reached 40 and 50. They told me to breastfeed him with 20 and give him the other 20 through a probe (tube).

**MODE: To give him 20…?**

W1: Via a probe

**MODE: Yes**

W1: 20 through a probe, and 20 through breastfeeding

**MODE: Yes**

W1: When it was over, I breastfed him for the third week, and when it reached Friday, we were discharged from the hospital and came back home.

**MODE: So, you came on Monday and left on Friday?**

W1: I returned home on Saturday, but I was discharged on Friday.

**MODE: You spent 6 days in the hospital?**

W1: I spent there 2 weeks, and for the third, it was on Saturday, so you understand that the third week was about to end.

**MODE: Going back a little back, the child is born, and as you observe him, you find that the intestines are outside, right? How did you receive that?**

W1: I did not receive it well because it was my first time to see and hear it. I felt that he would not heal or live.

**MODE: Would you share with me in more detail how you immediately felt, and all the things you thought about when you saw these complications?**

W1: After I gave birth to her at the health center, they told me to look at the other side, to prevent me from looking at my child and experiencing problems or falling off the bed. They immediately called for an ambulance, and it took us.

**MODE: And it took you where?**

W1: At Nyagatare Hospital. When I reached there, they asked me questions and I responded, but they also told me that the baby was wet during pregnancy, because the belly had so many fluids in it.

**MODE: Hmm**

W1: When I reached at Nyagatare, they called for an ambulance, and it took me. As we were going on our way in the ambulance, I was thinking that the baby will not survive. I was wondering if they will operate him and fix the intestines in their rightful place. I had so many questions in mind and felt that the baby was no longer with me. I reached there and they took care of him till Sunday, on Tuesday they placed the intestines inside the body, and none was left out. They took care of me since I could see people that had been there for 2 months.

**MODE: Hmm**

W1: So, I spent there another week and went back home. My baby was saved, and you also took much care of him.

**MODE: Yes. So, you told me that the people from the health center took you to Nyagatare Hospital?**

W1: Yes

**MODE: When you arrived at Nyagatare, how did it go for you to end up meeting a doctor in Kigali?**

W1: I arrived there, and they put him in an incubator for 5 hours. He spent 5 hours in there, and they immediately took us in an ambulance. They said that they cannot handle the case and said that there were specialists in charge of that who will handle the case. So, they transferred us to CHUK.

**MODE: Hmm**

W1: They said, “we cannot manage this, and we are also missing some equipment.”

**MODE: Yes**

W1: They inserted probes in him, but he was not on an oxygen supplying machine.

**MODE: Hmm**

W1: They put an oxygen mask on him on Tuesday, which was the day when they operated and fixed the intestines inside the belly.

**MODE: Ooh, so you reached at CHUK on Tuesday?**

W1: I reached there on Sunday, they operated him and covered his body on Tuesday.

**MODE: You reached at CHUK on Sunday?**

W1: Yes

**MODE: They fixed the intestines and covered them on Tuesday?**

W1: Yes

**MODE: When you got to CHUK hospital where they said you would find experts, what did the doctors tell you about the disease at the time?**

W1: I asked them if the baby would recover. They told me that when they bring ten similar cases there, four recover, and six die. I felt scared but I said to myself that since I brought him there, there were other children in the probes. There was a time when some of those close to me began to give me hope because they told me that the doctors would make it.

**MODE: So, when it came to obtaining information, did you ask doctors the root cause of that sickness? Tell me in detail the information they gave you about the disease, maybe you were curious and asked them why he was born like that. What did the doctors tell you?**

W1: I asked them, and they responded that it has no root cause…

**MODE: Hmm, I am not hearing you well**

W1: It has no root cause, and it is not a disability…

**MODE: Hello?**

W1: I can hear you, they said that it has no root cause.

**MODE: Okay, now I can hear you. What did they tell you?**

W1: That they do not know the root cause, but they said that it is like when a child is a born with a bent foot or arm and it becomes necessary for them to fix it.

**MODE: Hmm, that is all they told you?**

W1: In brief, they did not tell me the root cause.

**MODE: When you were admitted in the hospital for two weeks, as you told me…**

W1: Three

**MODE: Ooh, you spent there three weeks?**

W1: I spent there two weeks, but I did not finish the third week because it was only one day that remained to complete it.

**MODE: You spent there 2 weeks and 6 days?**

W1: Yes

**MODE: During those 2 weeks when you were with your baby at the hospital away from home, what challenges did you encounter when you were at the hospital?**

W1: I had a loss since I was there with no one to feed me, I could fetch breastmilk, and it requires you to eat nutritious and sufficient food to get breastmilk and give it to your baby. When you wake up, you take a porridge cup costing 300Rwf. You understand that I have lost a lot of things and in fact, I didn't have a lot.

**MODE: Share with me, you get that I do not know, right? I do not know how it went. You may share with me the day-to-day challenges you faced, the things you did not have as you are telling me, and how you bought porridge of 300Rwf. In general, tell me so that I can get a picture of the challenges you faced.**

W1: The challenges I faced were spending money every day when I do not have a source of income. I had to pay a lot of money when I left the hospital. I had three goats at home, but I had to sell them and pay for the hospital bills. I also sold one more.

**MODE: Hmm?**

W1: I exceeded a payment of 50,000Rwf because I paid 60,000Rwf.

**MODE: You mean all the payments that you made?**

W1: I did not include money for prescribed medications, you understand that I encountered losses. But at least I brought the child back home and he has no problem. I am still paying. The baby is now energetic, full of laughter and has no problem.

**MODE: So apart from financial challenges, are there other life challenges that you faced at the hospital?**

W1: I had no other challenges except that he could be under the oxygen mask, he used it twice, and I was feeling sad by then, but the good thing is that he dropped its use. They used to tell me that it will be difficult for him to survive without the oxygen mask, but I later saw that he managed to survive without it, and we went back home.

**MODE: Why did they tell you that leaving the oxygen mask will not be possible for him?**

W1: They could supply a little amount of oxygen, and later put a mask on him. They put a mask on him thrice. But he finished the use of that mask, and got support from a smaller equipment, and he also left it, then we were discharged.

**MODE: You said that he ended using the mask and shifted to a tiny one?**

W1: Yes, the one of less oxygen supply.

**MODE: Okay**

W1: So, he left the use of the smaller equipment and recovered, after spending a few days without oxygen support, we were discharged and left for home.

**MODE: Excuse me for the question I am about to pose, when we discussed you said that you found there other children who have similar conditions as your baby**

W1: Yes

**MODE: Their parents were also around, right?**

W1: Yes

**MODE: It was when you were sad, paying for a child’s medication, treating a child on oxygen support as you told me. It is hard for me to ask you about that time, while you were living with other mothers, the good things that you may have found in that situation. But no one knows, have you benefited from being at the hospital? The good thing that you gained from others who were with you, or something good you gained from the hospital?**

W1: There was nothing at all.

**MODE: Okay, whether in relations, putting aside material gains and looking at your relations with the doctors, was there anything that you found pleasant?**

W1: You mean the parents?

**MODE: Yes, whether the parents who were staying with you, or the doctors who supported you**

W1: We were happy, the people who went back home with their kids were happy, but those who were not discharged to leave for their homes with their children were not happy.

**MODE: So, your benefit was to go back home with a healthy baby?**

W1: Yes, that was the good thing I saw there. I find that you take care of them, making hourly check-ins. They are ever present, and that is what pleased us.

**MODE: You said something about hours?**

W1: In every hour at least there is a health worker, and minute by minute there are people.

**MODE: What kind of people?**

W1: Doctors are inside the room , 2 doctors and 1 nurse, I thank especially nurse Lydia who took special care of children.

**MODE: Yes, after that time, you told me that you and your child were discharged, right?**

W1: Yes

**MODE: What agenda/instructions did they give you concerning how you should take care of your child, and how did you follow those instructions?**

W1: They told me to go and take care of the child, through frequent breastfeeding, and I still do that. The baby is currently breastfeeding. They told me to return there after a certain time.

**MODE: Which appointment did they give you?**

W1: On 17th May I went there, and they told me to come back on 21st, because of the limited financial capacity I stayed there in the hospital, told my mother that it was not possible to return home and asked her to send me the money and she sent it to me. When the appointment date reached, they treated him and told me that my child had no problem. But what they had told me on 17th was to take a paper to Edmond so that he may check the baby via a scanning machine and see if he had a problem to fix a time for surgery. I did not find Edmond, and they told me to come back on 21st. When 21st reached, I took the child for treatment and found a man who told me to go home and come back. I asked him, "what kind of problem does my baby have?" I told him that the baby experiences pain during defecation, but now he recovered, and his navel keeps...

**MODE: What was happening with his navel?**

W1: There was intestinal movement around it but the intestines are getting to their rightful place, they didn't provide medication for that. I asked them, "Won't you provide medication for this?" They said that there was none. They told me to return after 2 years, on May 2023, I don't remember the date exactly.

**MODE: So, you will take the child back to the hospital in 2023?**

W1: Yes, that is when I will return.

**MODE: Okay, so back to what you were telling me about the instructions they gave you when you took the child back home, they only told you to breastfeed him and nothing else?**

W1: They told me to take care of him and breastfeed him, and as I am waiting for this growth, I should…

**MODE: You should…?**

W1: That I should carry him on my back after a month of recovery, and I also followed that instruction.

**MODE: Excuse me, I did not hear you. You said that since he was a child, you would follow which procedures?**

W1: That I would carry him on my back when he has recovered, that I should breastfeed him several times a day. They told me that I would carry him on my back after a month and I followed that. I covered him with a cloth to prevent him from getting cold. I followed every instruction and now the baby is healthy and has no problem.

**MODE: Yes, thank you. What do you think about the treatment services that your child obtained? What do you think about them?**

W1: I think that they should keep providing good services and treat others so that they may take their children back home as I took mine, and they leave happily, as I left the place feeling happy.

**MODE: Yes, going back to your child specifically, would you say that you were given good services? Would you say that the service was fair? What would you say about it as a mother if you were conversing with someone with a similar situation, would you tell her that you received good health services and recommend her to come for treatment? Or would you say that it was not good? What would you tell her about the services you were getting in those 3 weeks?**

W1: I would tell her that they provide excellent services.

**MODE: What good thing did you like about the services that pleased you the most?**

W1: How they treated my child and he recovered, they committed their time, second after second, minute by minute.

**MODE: How did they take care of him?**

W1: They could check if he has removed the oxygen mask because he was stubborn as a baby, they would control to see if he removed the mask, if the medication were over, they could just replace it with another one, just like that because my child could lack medication because whenever they went to search for it, they could leave some to me because I had taken 3 medicines. It was usually difficult to find that medication, but they could try their best and find it. They could approach him to avoid the swelling of the head or the arm, and they followed up to avoid any swelling on the body.

**MODE: Okay, so was there an urgent issue you faced at home after getting discharged that made you seek immediate medical assistance?**

W1: No, I went to the hospital, and I told them that his intestines were moving around the navel because he had a swollen navel. They told me that we were going to give me medicine, they gave it to me, they had also prescribed another one at CHUK, which exhausted, and they gave me another one. So, I bought the medicine and received the other one from the hospital, they told me how to give it to him in order, and I did it. He endured constant pain, but it later ended. I had brought the baby to show the external intestines to the hospital, and they said that it was not that problematic.

**MODE: Do you remember the medication that they had prescribed for you?**

W1: I do not recall.

M1: It is here, let us check. We have it but since we kept it for long, the words are no longer readable on this paper.

**MODE: Okay, no problem. Apart from the issue of the child experiencing swollen intestines in the navel, didn’t you tell me that it was the case?**

W1: Yes

**MODE: And he could experience pain during defecation. Wasn’t there any other problem that made him to return to the hospital? Or a challenge he faced that became a burden to you?**

W1: We were at home together, and we took him to the hospital. I took him to the health centre, and he was given 3 pills to pass through the anus for pain relief. Since then, I have not taken him back to the hospital.

**MODE: So, you never came back to CHUK?**

W1: Yes, and he has not taken any medication since then. I will take him to receive the third dose of the vaccine today.

**MODE: It is the dose of which vaccine?**

W1: Just normal vaccination

**MODE: Let me call you once again, my airtime is about to exhaust.**

W1: Okay

**MODE: Hello**

W1: Yes, I can hear you.

**MODE: Okay. We were discussing an emergency issue your child may have encountered that made you bring him back to the hospital. You were done telling me about that, right?**

W1: Yes, I did not bring him back to the hospital.

**MODE: Come again?**

W1: I did not bring him back to CHUK because of sickness. He never got that sick.

**MODE: Okay, can you tell me about your experience of living with the baby at home? During his sickness as you were discharged from the hospital, and how you stayed with him?**

W1: I stayed besides him…

**MODE: Sorry, come again?**

W1: He never had any issue, whether with breathing or any other concern, he was calm and never had a problem.

**MODE: How was it to take care of him?**

W1: Another thing they had told me was that I should not give him to people for them to carry him, they told me to keep him in the room, cover him with a cloth to prevent cold. Whenever he left a warm place, I could do that. The set time they gave me for carrying him on my back was well respected, I could do it for like an hour in a day.

**MODE: You said that you were allowed to carry him on your back for one hour per day?**

W1: They did not tell me that, since that was the recovery time, I could carry him for an hour to prevent him from getting tired because of the problem he had. So, I had to help him get used to it.

**MODE: Okay, can you share with me the financial challenges that you encountered?**

W1: The challenges I faced; I could eat in the morning. I could take a cup of 300Rwf in the morning and in the evening.

**MODE: I am not hearing you, try to be audible enough**

W1: What kind of challenges I am supposed to talked about?

**MODE: Financial challenges**

W1: When I was at CHUK, or when I was at home?

**MODE: You may tell me both places, no problem.**

W1: The hardest part for me was that I was not financially stable, and I needed to eat breakfast and dinner. It was also my first time fulfilling these responsibilities since it was my first time giving birth. It was very difficult for me, and I did not even have a job. That is where it started becoming a challenge.

**MODE: You were telling me that in the morning you were buying a cup costing how much?**

W1: When I was at CHUK, I could buy a cup of porridge costing 300Rwf in the morning and in the evening, cassava leaves at 10 a.m. to get breastmilk. You understand that it was hard for me. They brought us food at noon, and we could eat. Since I was with my mother-in-law, you understand that she also needed to eat. You cannot eat and let her starve, so the treatment was a huge loss for me. The little I had achieved also vanished.

**MODE: That was your experience at CHUK. When you reached at home, which financial challenges did you face?**

W1: I reached at home …

**MODE: I am not hearing you**

W1: I reached at home and neighbors started helping me. I could count on them because they were the ones who knew my situation.

**MODE: What were the mental or emotional challenges that you encountered? You see that we have emotions as people, right?**

W1: Yes

**MODE: Your child had a problem; you were not happy. Were there any mental or emotional challenges or scars that you experienced because of the situation?**

W1: There were no challenges other than…

**MODE: Speak louder**

W1: I had no challenge other than paying the debts that I took from people to cover some bills.

**MODE: Like who?**

W1: My husband borrowed funds and sent them to me. I would not stay in the hospital when the baby had recovered. They had discharged us on Friday, and we had to find means to bring the baby back home. My husband had borrowed money from a neighbor who showed bravery and said to him, "let them come home, you will pay me later," and my husband responded, "I will repay you in February when the harvest is ripe, but the farm he digs in is not ours."

**MODE: When you were taking care of your baby as he had that complication, wasn’t there a time when you felt sad? Or heartbroken?**

W1: This is inevitable when the baby seems to be breathing well, and in a while, you find his stomach sinking and the heart sinking. You feel very sad. I said to myself that I would go there in 5 hours because it was sad to see my baby...

**MODE: You said that you felt like going back after 5 months?**

W1: After 5 hours but I could check him every hour to see if maybe his heart was back in its place and if he were breathing well. They could put an oxygen mask on him. I could feel very sad, but I later saw that he recovered.

**MODE:** **Okay, so you see when someone is pregnant, every pregnant mother has a way of expecting her child, there are things you think you will do for her, in fact, there is a way you are planning for the child. Isn't that right? This happens to all pregnant women. Before the time of birth arrived, how were you expecting it to be?**

W1: I thought that I will give birth at the health center and later bring the baby at home as I have seen it with others. I could see them coming back to the health center and I was thinking that it would be the same case, but it was not the case.

**MODE: What changed with regards to your expectations?**

W1: There was a change because I did not meet my goal due to the challenge that I encountered.

**MODE: Which goal did you have?**

W1: Since I had goats, I had a goal of buying a plot of land so that he may grow up finding it there, but I did not realize that goal.

**MODE: So, can you share with us how it changed your life within the community? You understand that everyone is not used to seeing a newborn baby having a problem especially having external intestines. How has it changed your way of living with community members or neighbors?**

W1: I had no issue with the neighbors, however there was…

**MODE: Hmm?**

W1: When you leave, neighbors come asking you how it went, and you feel like telling someone the whole story and disclosing it from someone else.

**MODE: Why do you tell some people the whole situation and leave out others?**

W1: It depends on your level of friendship.

**MODE: On that point, I have a curious question**

W1: Yes

**MODE: Why would you only tell someone the problem of your child because she is your friend, and not tell the other person about your child’s situation if she is not your friend?**

W1: It is because a friend is the one who helped you when you faced those challenging moments.

**MODE: What did the neighbors say about the disease that your child suffered from?**

W1: They all said that it was their first time seeing that case, others could say that it’s their first time hearing that, and old women would ask, “do those things really happen?”

**MODE: So, between you and your husband, was there a change in your relationship compared to how it was before you gave birth? How did it go?**

W1: It did not change anything because we are still together, we accepted it and….

**MODE: Hmm?**

W1: We accepted it and kept living together as it used to be. We did not encounter any relationship challenges.

**MODE: He kept supporting you and you are still together?**

W1: Yes

**MODE: Okay, thank you for sharing your personal information, right?**

W1: Yes

**MODE: How is the child doing?**

W1: The child is doing well; he laughs and has no problem.

**MODE: For the matters concerning his health, he no longer has a swollen navel?**

W1: No, it keeps reducing slowly by slowly.

**MODE: In comparison to how it used to be, it has been how long since it came back in order?**

W1: It has been like a month.

**MODE: It has been how long?**

W1: A month

**MODE: That was when the intestines started leaving the navel?**

W1: Yes, it keeps leaving the place slowly by slowly. It is not that much.

**MODE: Hmm?**

W1: You could touch and feel certain things moving, but those things disappear slowly by slowly, and the swollen navel diminishes.

**MODE: Would you say that he is healthy with no problem?**

W1: He is healthy with no problem.

**MODE: Does he cry frequently?**

W1: He does not cry that much, he is calm.

**MODE: Doesn’t he vomit because of the complication he had at birth or experience any other health related challenge?**

W1: He does not experience any other challenge, I took him to the hospital when he was vomiting, and even when the intestines were going in the navel. There was nothing else and he has recovered.

**MODE: He could vomit because of what?**

W1: He could vomit after exceeding the standard quantity in breastfeeding, and when he vomited, I could wonder if it was the root cause. I kept getting confused and I took him to the hospital, and they gave me a medicine to use. The medicine was not useful, and I returned it with other things…

**MODE: Hmm?**

W1: I returned it with other things, and they exchanged it with other medicine, but it also yielded nothing. But now he has recovered.

**MODE: The medicine they gave you when he was vomiting was not useful, but he no longer vomits?**

W1: Yes, it reached a certain point I realized that it had reduced.

**MODE: How is his feeding mechanism? Does he breastfeed or he drinks soya milk? Or the usual milk?**

W1: He breastfeeds, I have breastmilk and he breastfeeds.

**MODE: Don’t you mix it with something else?**

W1: No

**MODE: How old is he?**

W1: 3 months and a half

**MODE: 3 months and a half**

W1: Yeah

**MODE: How is his growth?**

W1: He grows well and has no problem.

**MODE: His weight is the same as that of a normal child? When you take him to the hospital and they measure his weight or height, do they say that it is normal?**

W1: Yeah, there is no problem. He is growing.

**MODE: Hmm**

W1: When I took him to the hospital for the first time, he had 2.5kgs

**MODE: Hmm?**

W1: He had 3.5 kgs, the second time he had 4.5kgs, I do not know how many kilograms he will have today since they will operate his heart.

**MODE: Ooh, so you are taking him back today?**

W1: Yes

**MODE: Do they tell you his height?**

W1: They never told me that.

**MODE: Okay, so you see that you have come a long way in treating your child at the hospital, and you went through hard times. Can you tell me something you wish to have known about your child's illness or the care he needed? Since you have been through it, what information do you feel you needed before, maybe if you knew it would have made a difference to how you would have treated/taken care of him? What do you wish to have known before?**

W1: I would like to know the root cause of the whole situation to prevent it from happening the next time.

**MODE: Apart from this wish you currently have, right?**

W1: Yeah

**MODE: I am asking you because you went through it all. What information would you wish to have known when you were pregnant and say that if you knew what this would be, I would have done things differently? What kind of information like that would you have wished to know?**

W1: Knowing it during my pregnancy would have been worse because I knew it after giving birth.

**MODE: Hmm?**

W1: Knowing it in my pregnancy would have been worse than knowing it at the child’s birth. I would feel depressed and wonder how I would give birth to a child like that one and wonder how it would go.

**MODE: What message would you address to a parent whose child was born with complication like that of your child? If you met and she said that she gave birth to a child with external intestines, what would you tell her?**

W1: I would tell her, “Do not feel depressed because he will recover, take him to this place, request for a transfer and take him to this place because it will be better for you.”

**MODE: You would tell her to take the child where?**

W1: I would tell her to take the child to the hospital and get a transfer to CHUK since they are the ones who will help you and your child will recover.

**MODE: Okay, do you have anything to add to the discussion that we have had together?**

W1: I would like to ask the root cause of that situation.

**MODE: The root cause of the situation?**

W1: The reason that caused the child to be born like that.

**MODE: At this very hour, in my little knowledge concerning this matter and referring to what they told me, they do not know the root cause, you get?**

W1: Hmm

**MODE: But it usually happens to the first babies as you also told me it was the first baby you bore. But when you go to the hospital, they may see the baby in the womb and prevent it when it is at its early stage. That is the information I have that I may tell you about this, but for the rest, as I told you, they also do not know. They cannot specify that this is the main root cause.**

W1: When I went for the pregnancy test, they could tell me that the baby is alive with no problem, but in me, when I was pregnant, I had a problem. I would touch my belly and feel there were two heads and I wondered if I carried twins. But they tested me and told me it was one fetus, but he didn't really play like the other babies were playing in their mothers' wombs. I asked and they told me that he had never played so much that even the parents who were with us there also told me that the child had never played. Was he playing in a secure manner to avoid getting hurt?

**MODE: You said that when you were pregnant, you were not feeling well, how were you feeling? What specific thing did you feel in you that was not normal?**

W1: You see, when a person is pregnant, the womb is in good shape, but mine was split in two. You see, there are two sections, you hear one side with the head, the other with the head, and the other is at the top and not at the bottom. Others could tell me that he is upside down; I would touch the lower part and feel nothing. I would wonder, "where is the baby's heartbeat?” They could tell me that I have no problem and the child has a normal heartbeat. I could ask, "how come others I saw were having wombs in perfect shape, and mine was at risk of not growing, and he told me to come and get a transfer. He told me to come and get it on Wednesday. On Wednesday, I did not go there, the following Wednesday, I was going to go there, and I saw the dates approaching. So, you understand that I did not have to go there on Wednesday, I went to give birth in June, and I immediately found out the problem.

**MODE: As we approach the end of our discussion, which additional point or recommendation would you like to state?**

W1: My wish is to find out the root cause of this situation.

**MODE: Do you have any questions?**

W1: Questions are ever there.

**MODE: Questions concerning the discussion we had?**

W1: No

**MODE: Okay, if you have no problem, this marks the end of the conversation. Thank you for your participation; you spared your time for us and gave us information. I was glad to find out that your child has recovered and that he is growing up well. May God continue to raise him. And thank your husband on my behalf. I do not know if he is listening to me. Thank you for giving me your time.**

W1: Yes

**MODE: Thank you so much**

W1: Yes, thank you as well.

**MODE: Yes, have a nice day.**

W1: Yes.
