# Supplementary material for: Exploring the lived experiences of parents caring for infants with gastroschisis in Rwanda: The untold story
Source: PLOS Glob Public Health. 2022 Jun 15;2(6):e0000439. doi: 10.1371/journal.pgph.0000439 (PMC10021215; doi:10.1371/journal.pgph.0000439)
Supplement: S1 Data — (ZIP) [file pgph.0000439.s002.zip › S1_Data/S11_Text.docx]

**BB3 Transcript**

**MODE: Thank you for accepting to talk to us. As I told you, the objective of this interview is to find out how your child was taken care of at the hospital and how you also took care of her. We would like to utilize this information in helping CHUK make changes in service delivery, but we won’t reveal your names or your identity to anyone. Do you have any question before we start?**

W1: None

**MODE: When was your child admitted to the hospital?**

W1: On 29^th^ April

**MODE: I’ll need you to speak louder so that the recorder may grasp you well, right? Because if you speak softly, I won’t hear you.**

W1: Hmm

**MODE: I’ll hold it from here. Speak louder so that I may catch your voice.**

W1: Yeah

**MODE: You told me that your child was admitted to the hospital on which day?**

W1: On 29^th^ April

**MODE: This year?**

W1: Yes

**MODE: You took her to CHUK after how long?**

W1: We had reached there on 29^th^ April

**MODE: Where were you before that time?**

W1: I was there all that time and left on 24^th^ May

**MODE: You went to CHUK on 29^th^ April**

W1: I gave birth on 28^th^ April at around 3 p.m. and they transferred me to CHUK. I reached there around 11:00

**MODE: Of the following day?**

W1: Yes, that’s when I reached there.

**MODE: For how long did you stay at CHUK?**

W1: I left the place around this time on 24^th^ May

**MODE: That was almost a month.**

W1: Yes

**MODE: How old is your child?**

W1: She’s turning 2 months on 29^th^

**MODE: Two months. He’s a boy/ a girl?**

W1: A girl

**MODE: A girl. Now, I would like to start from the moments you had in CHUK on 29^th^ April this year. You and your child went to CHUK that day, is it true?**

W1: Yes

**MODE: Can you tell me in details what happened that day when you reached at the hospital? I mean, not when you reached at CHUK, the moment when you gave birth to your child till the time when you were at CHUK. All the things that happened, and the experience you got from them. Tell us in details about the assistance that you got from doctors, and what anyone else did concerning you and your child.**

W1: After giving birth to the child, since they operated me, they showed me the child but told me that my child has a problem. She is born with a problem of external intestines. So they said to me that they are transferring us to CHUK since that’s where they can treat such conditions. Since they had operated me and anesthesia was still active in me, when the anesthesia exhausted, the ambulance and took us to CHUK. When we reached there, they welcomed us and took care of us. They started treating this one and put her in an incubator. They treated her and I found that there was no problem.

**MODE: Those are the only things that happened?**

W1: Yeah

**MODE: How did you react when they told you that you had given birth to a child with external intestines?**

W1: Accepting it was difficult, I first thought that it was witchcraft, and thought that someone bewitched me. But I later accepted it. I also thought that it was because of my work since I worked while sitting, but when I reached at CHUK, I saw other children and felt humanity in me. I understood that it is a normal disease, there were also others that came and I saw that it is an existing situation, and I realized that I wasn’t poisoned/bewitched.

**MODE: Hmm**

W1: That’s when I accepted it when I found there other people as new ones also stepped in.

**MODE: What did the doctors tell you during that time about the sickness of your child?**

W1: I asked them as I was telling you. I asked if the disease already exists, but after seeing others, they told me that there were children who were also treated from the place that recovered and went back home.

**MODE: He didn’t tell you anything else?**

W1: It was just that discussion where he told us how to take care of children, and he told us how to maintain their cleanliness, that we shouldn’t annoy them, that is what they taught us.

**MODE: Why is it not allowed to annoy children born with this condition?**

W1: Because they are born with a problem. When a child is born normal with no problem, and another one is born with a problem, those two are not the same.

**MODE: Did they tell you that it applies to the time when they have grown? Or even now when they are still babies?**

W1: From now onwards till their growth. Unless they have grown, but now they are to be protected against that.

**MODE: What challenges did you face when you were at CHUK?**

W1: The challenges?

**MODE: Yes, tell me all of them, whether financially, in terms of relationships, or sickness. Anything that was challenging to you when you were at CHUK.**

W1: Nothing

**MODE: There was no challenge?**

W1: Yes

**MODE: The funds were not an issue in the treatment of your child?**

W1: It was not an issue

**MODE: You had funds? The treatment of your child was not challenging?**

M1: The government paid for the bills

**MODE: The government?**

M1: Yeah

**MODE: You had CBHI (Community Based Health Insurance)?**

M1: Yes, we had CBHI and she was treated on the insurance of her mother

**MODE: The child?**

M1: Yes, since her mother is in the first category, the baby was treated on the insurance of her mother

**MODE: CBHI covered for every hospital bills? You didn’t even pay a coin of 100?**

M1: Not even a coin

**MODE: You told me that she was in the first category. Those from the first category, CBHI covers everything from Kibagabaga and the child’s treatment is also covered?**

M1: Yeah

**MODE: Wasn’t there anything else that was challenging apart from the funds? You told me that you carried food and were also there. Were you there all alone?**

W1: I was with my mother-in-law.

**MODE: Wasn’t there anything that burdened you at CHUK?**

W1: It was eating since the plates were expensive, this one brought us food

**MODE: Apart from that, everything else was going well?**

W1: Yes, there was no problem.

**MODE: After getting discharge from the hospital with your child, what plan did they give you for taking care of your child, and how did you follow it?**

W1: They told me that I have to maintain her cleanliness and take care of her by breastfeeding her.

**MODE: Those were the only things they told you?**

W1: That’s what they told me to do.

**MODE: Did they tell you that you have to bring back the child?**

W1: I go there on appointments. The first one was on 7^th^ June. I went there and they gave me another one of 19^th^ July. I’ll go back there and see how it is.

**MODE: You got the first one on 19^th^?**

W1: The first one was on 7^th^ June, I went there, and they gave me another appointment on 19^th^ July.

**MODE: When you went there on 6^th^, what did they tell you?**

W1: He told me that the child is recovering, he covered her wound and told me to remove the cover after one week, and later clean her with a clean cloth, and protect the area from water. For the area to merge, he also told me that glue damages the wound, he said that I should remove the glue he placed there and place there a clean, ironed cloth. I did that and the area dried.

**MODE: Wasn’t there anywhere else where people from the hospital told you to take her even though you were not obliged to return to CHUK?**

W1: Since they saw that I live near, they refused to transfer me to a health center because they saw that she was recovering. They told me that there is no problem, I’ll have to bring her. I went there on 6^th^ and got an appointment on 19^th^ July, that’s when I’ll return.

**MODE: Ever since you came here, was there an emergent issue that required you to take her to the hospital? Like a child getting sick and in a critical condition after getting discharged from the hospital?**

W1: No

**MODE: Ever since you were discharged, she has not fallen sick?**

W1: Yeah

**MODE: She has not faced any other problem?**

W1: Apart from the vaccines she received that got her some side effects.

**MODE: When?**

W1: I vaccinated him on Thursday but she was still having pain in the thighs.

**MODE: It was a vaccine for what?**

W1: It was a vaccine for a month and a half.

**MODE: They vaccinated you against what?**

W1: Polio, tetanus, whooping cough, measles, they are many

**MODE: It was just those side effects from the vaccine but she didn’t get any challenges as a result of that disease?**

W1: Yeah

**MODE: Proceeding on what I was asking you, you can tell me how it is to live at home with a baby, in the usual life, how is it?**

W1: In the usual life, there is no problem

**MODE: Taking care of her, is it simple? Is it complicated? She is the second born?**

W1: Yes

**MODE: Comparing her to the first born, what would you say was the difference between raising the child born with a problem and the child who was not born with a problem?**

W1: For this one, I feel worried because she has a problem, it requires too much care.

**MODE: Can you tell me the challenges that you face while taking care of her? Whether in form of work? Finances? Or if it even affects your mental health? You can tell me all of that.**

W1: It doesn’t affect me and there is no problem with the finances

**MODE: No problem, how about the work that you do?**

W1: The work that I do?

**MODE: Yes**

W1: I am not working but I was used to doing subcontracted work

**MODE: Like what kind of work?**

W1: Sewing

**MODE: For you, do you face any challenges because your child is like thus?**

M1: The challenges include spending so much time, having a great commitment, and ensuring that there is someone near the child because she needs someone besides her every minute since she is stubborn. She cries and needs someone close to her. She is not like a child you may leave somewhere and return thinking that there won’t be any problem. She is a child requiring the nearby presence of her mother.

**MODE: Okay, comparing to Sonia whom you bore and this one, what is the difference between raising both of them?**

M1: The difference between raising them?

**MODE: Yeah**

M1: The difference is that the first born was born healthy with no problem but this one was born with a serious sickness that is more than one.

**MODE: Which other diseases was she born with?**

M1: She has another sickness with her head, her fontanelle is here, yet for other children it’s there. You understand that she may cry because of the disease of intestines or the shape of her head. So, I can’t identify what makes her cry, and that’s the challenge. There are times when I think that it’s the head when it’s the intestines. So, that’s the first and foremost challenge.

**MODE: Did you ask the doctor why her fontanelle is in front?**

W1: I saw it when I reached here

M1: We’ll ask him when we go back there

**MODE: You haven’t asked him yet?**

M1: Yeah, we haven’t asked him. We saw that after she had returned. We’ll ask about it on the appointment day.

**MODE: Okay, how did it change your life in the community with others? When they found out that you had given birth to a child with external intestines, what did they say about it? How did they receive it in general?**

M1: It was new to them since apart from them, it was also my first time to see it. They thought that it was something unusual, and they thought that she would not survive, but the doctors who treated her stated that it was something usual, and that it would recover.

**MODE: For you, what would you say on that?**

M1: I would say that doctors taught us and told us that it already existed before, and they told us that it will come to an end. They could comfort us. They told us about children who had departed and those who were there, there were those they had discharged when we were there. They told us that it will recover, and that it’s something normal.

**MODE: How about the neighbors? I guess they said something different. Were they those who had different information about the disease and made unusual comments?**

W1: You mean the neighbors?

**MODE: Yeah**

W1: The neighbors took it as something strange as whoever heard it could say, “oh my God! Does that exist? The birth of a child with external intestines, do you think it’s possible?” Then you tell them that it’s possible and that there are other children who recovered. You can tell her that there are others who are in the hospital. You just accept it.

**MODE: Those from your family, how was it?**

W1: In our family they just believed what we told them. We told them that it is an existing disease and not a new one. We told them that there were children who recovered and came back to see other children. They also accepted it as we accepted it.

**MODE: Okay, between you two, did the birth of a child with this complication affect anything? Or it increased something? If there is any positive outcome that you got from CHUK, you may also share it. Bad things happen indeed, but there is also a time when you sometimes go through bad things, but you find someone who comforts you, or you also benefit other good things. What good things did you get from the sickness of your child? I don’t want to say that it’s pleasant, but sometimes we meet with good things.**

W1: There are times when you don’t find pampers for your child and your relative calls and sends that money. So you don’t feel desperate, they call you and tell you that she will recover. There are times when I found that they had injected the serum when she had swollen, and they could tell me, “do not worry, she will get back in shape, do not worry.” You could feel like worrying but when your relative calls to ask how she is doing, you tell her, do not worry since serum may cause stomach swelling but she will recover, do not worry. And s/he could comfort you like that, send you some money, and you feel calm. Even the doctor could say, “Don’t worry she will recover, and you would feel comforted.”

**MODE: For you, do you have any additional point?**

M1: Me?

**MODE: Yeah**

M1: I can’t miss it. It made me realize that I was born alone. Since I come from a poor family and I am poor as well. The challenge that the child felt, they thought that it was huge. You see a poor person taking care of a sick person at CHUK, and he/she is treating a disease that no one else has ever seen, you feel scared. However, there were those prayed for us, those who encouraged us and said take heart. It made me realize that if I had any other issue, I would have people to support me. I saw that I wasn’t alone, and I don’t have to be selfish because I have friends. That’s what I gained.

**MODE: Amongst you, how did your relationship change after realizing that you had given birth to a child with a complication?**

M1: The relationship?

**MODE: Yeah, the way you used to live together before, how did it change? Was it a positive or negative change? Or it stayed the way it was? How was it between both of you?**

M1: It stayed the way it was, nothing changed

**MODE: How about you?**

W1: No problem, it stayed the way it used to be

**MODE: Thank you for sharing with us the information about the life of your child, I’ll ask this question to you because you are the one who carried her in your womb. You see when you are pregnant, there is a way you imagine how it will be when you give birth to your baby, you will do this, especially that she was the second born. All the mothers have such feelings, right?**

W1: Yes

**MODE: When you were pregnant of your child, comparing your expectations and what you had planned to do for your child with the reality that you faced, how did you receive it? How did it make you feel?**

W1: I got pregnant of the baby when I wanted her (a child). The first born was getting a bit aged and spoilt. I said that I needed a child after her. Even though she was born with a problem, I accepted the situation. When I gave birth to her, I said that since I wanted her, I felt that I had a problem. But after reaching at CHUK and seeing other children, and realizing that the disease was existent as I heard it from doctors, I accepted it and said that she belonged to God. If she’s meant to be mine, she’ll be mine, and if she is not meant to be mine, she won’t be mine, that’s what I told God and that’s how I accepted it. Since I wanted her, I said that if God sees that she’s mine, she’ll be mine.

**MODE: Okay, do you have anything to add onto what she said?**

M1: That’s it, since we gave birth to her when we wanted her, it wasn’t by accident because we had observed the age of the first born. Since we wanted her and she didn’t come by accident, we had to accept her not matter which way she came because we wanted her. There was no complaint of saying, why did I give birth to this one? Why is it happening to me? So, we just took it the way we saw it.

**MODE: Thank you for sharing us about the life of the child, how is the child doing now?**

W1: She’s doing well and she has no problem

**MODE: Hmm, her life in general, does she ever experience pain?**

W1: For pain other than crying, there are times when she cries, but it is not that much. Sometimes she faces difficulties in defecating, but she defecates.

**MODE: Does she ever vomit?**

W1: Sometimes she breastfeeds and vomits a few after breastfeeding, and there are times when she doesn’t vomit.

**MODE: Does she ever suffer from diarrhea? Basing on the way you observe her defecation**

W1: Apart from yesterday when I inserted a pill because I had taken her for vaccination and they had said that she has fever. The pill that is passed through the anus, I injected it inside, but I saw that she got diarrhea and got something like blood.

**MODE: That was the only diarrhea scenario that she encountered?**

W1: Yes, she defecated bloody feces. It wasn’t blood in clots but blood in liquid form, but it was clear that it was blood. It was just blood mixed with feces, but I have seen that now it has recovered.

**MODE: Did you ever take her to the hospital?**

W1: No, I didn’t take her. I thought it was a vaccine side effect, or the pill, and now it’s over.

**MODE: It’s over and now she’s defecating well?**

W1: Yes

**MODE: Does she only breastfeed or there are times when you give her milk?**

W1: She only breastfeeds

**MODE: You haven’t mixed anything else?**

W1: Nothing other than breastfeeding her

**MODE: Breastfeeding her only?**

W1: Yes

**MODE: Comparing how she was when you brought her from the hospital, how does her weight change?**

W1: Her weight increases since she was born with 1.900kg, and when she got out of the incubator, I didn’t measure her weight. When I took her for vaccination for the first time, she had 2.400kg, yesterday when I returned, I found that she had 2.800kg

**MODE: Now she has how many kilograms?**

W1: 2.800kg

**MODE: Do you remember to check for her height?**

W1: I didn’t check for her height, I only look at the weight

**MODE: Referring back to how she was when she was born, do you see any growth?**

W1: Yes, I see growth

**MODE: Is she a baby like others? Is she playful? How is she doing?**

W1: She is playful and she laughs, even though it may be things from the stomach that make her laugh. Since she has not yet got a clear sight, I see that it’s things from the stomach that make her laugh. There is no problem.

**MODE: What do you mean by things from the stomach?**

W1: There are times when she gets stomach complications and they make her laugh.

**MODE: Apart from the vaccines that we talked about or going for the appointment, the child has no other problem and you have not recently taken her back to the hospital?**

W1: Yes, the challenge I had, I called Dr. Edmond to ask for clarifications and he didn’t pick up the phone. I wanted to ask if she recovered to an extent that I may carry her on my back.

**MODE: That was the question that you had**

W1: Yes, because I have not yet carried her on my back

**MODE: She has not yet had any other problem? Like the way that wound looks like?**

W1: She has not yet had another problem

**MODE: Can you tell me something you wish to have known about the health of your child or the care that was needed before all this time? Whether before giving birth or before getting to the hospital, what do you wish to have known? Like when you sit and say, “Had I known this, maybe this would have changed.” Whether when you were at CHUK or when you carried her in your womb, which information do you wish to have known?**

W1: Since I did my job while seated, I thought that it had caused the condition, and I felt that I should have left it to prevent this from happening. That’s what I was thinking after seeing that she is alive. I was comforted when I reached at CHUK and saw other children.

**MODE: For you, what do you wish to have known, which information do you wish to have known before all this?**

M1: Before the birth?

**MODE: Yeah, whether before or after the birth, there are times, depending on how you see her, when you reflect and say, “Had I known this, it would have changed like this.” Maybe in your heart, does it ever happen to you and you wish to have known something before?**

M1: If I had known that the disease exists, it would have been easier for me to accept it.

**MODE: That was the information you wanted to know?**

M1: Yes

**MODE: This question concerns both of you, what would you tell a parent having a child with a similar condition as yours? If she gave birth to a parent with external intestines since it happens to many parents, we are going to start from you.**

W1: What I would tell her is an advice. The first thing is that when you see it, you have to accept it. After accepting it, you get comfort from others since there are times when you get to the hospital feeling sick and when you see someone who is also sick, your sickness may recover because you saw someone in a worse condition than yours. So, the advice I would give to her is to accept that such children exist and that her child will recover. That’s my advice to her. I would tell her that accepting it was challenging to me, but that when she accepts it, her child lives and grows well with no problem.

**MODE: Yeah, another thing? Since you went through a lot, I guess you have more than one piece of advice for her.**

W1: About the long journey?

**MODE: About the child, which other advice would you give to her?**

W1: I would advise her to take care of that child and not forsake her. She should be near her, do cleanliness for her, and that’s it.

**MODE: As the father of the child, which advice would you give to parents who face a problem similar to what you faced?**

M1: The first thing is to see that if a child is born with that condition, s/he shouldn’t think that the new born baby is no longer a child. S/he should understand that s/he has to do a lot for the child beyond what s/he did for a child born without a problem. S/he should understand that the baby is also a normal child and play with him/her like the way s/he plays with a normal child. She should breastfeed her while smiling and feel that she is a child like others who is suffering from a curable disease. It is a curable disease, and for the rest, she has to pray and follow the instructions of the doctor. That’s the advice I would give to the parent.

**MODE: We are nearing the end of our discussion. These were the questions that I had prepared for you, but if you also have a question or suggestion, you may let me know.**

M1: A suggestion. I think that CHUK did their level best since what they did yields results. In case another problem arises…it is to thank them for approaching us to see how our child is doing. I don’t have anything to add on since if you came and found a problem, you would have assisted us. But I think that what they did for us at CHUK and what God did for us is indeed yielding results.

**MODE: Okay, how about you?**

W1: I also don’t have a question since my child is recovering. He had told me to remove the wound cover and I was at first scared. I first refused but later did it. I removed it, took care of the part and it has recovered. I feel that they have helped me when I refer to before when they were hung. It was my first time seeing the part after uncovering her. I could uncover her without looking at the part to prevent myself from getting scared, but I uncovered her and saw that there was no problem, and she recovered. I thank you for taking your time to come and discuss with us. We also need comfort to know that it happens.

**MODE: Okay, thank you. What part do you receive from the neighbors which is not related to finances? Do they understand the problem that you have? Do they help you to understand your situation? Don’t they hurt you more? How would you describe your relationship with people from the community?**

W1: We have a good relationship, there is no problem. They accepted it and felt that there is no problem.

M1: There is no problem, and you also know about Kigali, we have limited time and we don’t spend much of our time with them, but in their little time we have no problem with them

**MODE: Hmm, they visit you, you visit them**. **They comfort you?**

W1: Yeah

**MODE: Thank you**

W1: Hmm
