# Supplementary material for: Exploring the lived experiences of parents caring for infants with gastroschisis in Rwanda: The untold story
Source: PLOS Glob Public Health. 2022 Jun 15;2(6):e0000439. doi: 10.1371/journal.pgph.0000439 (PMC10021215; doi:10.1371/journal.pgph.0000439)
Supplement: S1 Data — (ZIP) [file pgph.0000439.s002.zip › S1_Data/S2_Text.docx]

**BB 2- English Transcript.**

**MODE: Thank you for accepting to talk to us, the objective of this interview is to find out how your child was taken care of at the hospital and how you kept taking care of her. We would like to utilize this information to help CHUK hospital make changes in service delivery, but we won’t reveal your names or identity to anyone, right?**

W1: Yeah

**MODE: Since I have a recorder, it will require you to speak loud like me so that I may hear the conversation that we had. Let me place it here so that the baby doesn’t knock it off.**

W1: Hmm

**MODE: When was your child admitted to the hospital?**

W1: 15^th^ February 2020

**MODE: Yes, what was the span between her birth and taking her to CHUK?**

W1: It was on that day after 3 hours since I gave birth at 7:45 and we reached there at 11:00

**MODE: On that day, 15^th^ February?**

W1: Yes, on that day we slept at CHUK.

**MODE: For how long did you stay at CHUK?**

W1: Almost a month because I left the place on 11^th^ March, it was a just few days remaining to complete a month.

**MODE: Hmm, I can see that she’s a girl, how old is she?**

W1: She’s a year and 4 months old.

**MODE: She knows how to walk by herself? I am seeing that she might fall.**

W1: No, she can walk, it’s just that she is scared, but she can walk. She is learning how to walk.

**MODE: Okay, in this conversation, feel free and tell me all the information you have, how it went, the experience you had, what the father of the baby told you, what doctors told you. This is what will help us in changing the service delivery at CHUK, and help parents whose children have a similar problem to that of Etoile, right?**

W1: Yeah

**MODE: Do you have any question before we start?**

W1: I have no question, maybe you will ask me because I don’t have questions.

**MODE: Okay, I would like to start from the moments you had at CHUK on 15^th^ February, you and your child were at CHUK on that day, is that true?**

W1: Yes, it was me, my husband, and the child.

**MODE: Can you tell me in details what happened when you were at the hospital in that one-month stay? When you left Ruhengeri and reached at CHUK, all the things that happened to you, whether good or bad, or challenging, you may tell me all of that.**

W1: Yes, after giving birth, without lying to you, I felt like it was a punishment from God, because in my nature, I didn’t like children. I didn’t like children and wanted to live alone with no stress because I saw that with having a child, things change. Whether through carrying him, it gives you a lot of responsibilities. So, after giving birth, I felt that it was a punishment from God because before the birth of my child, if there is no problem I may talk about it.

**MODE: Say it all, whatever you want to say**

W1: I never thought of myself as a mother. I could imagine my classmates, passing by them, and I could feel that it was shameful. It was shameful but I wasn’t sure if I am ready to give birth. You know the way parents feel excited and say that they are going to buy a baby carrier for my baby, or when they go to check the status of the child in the echography. That was when I thought that maybe God is taking her away from me because I don’t want her.

**MODE: So, you got pregnant of her when you didn’t want it?**

W1: No, it’s not that I didn’t want it. There is a time when you need something but you’re not sure that it’s what you want.

**MODE: When you feel that you are not yet ready**

W1: Yeah, when you are not ready. So I thought that it was a punishment from God. The first thing that came into my mind was her father. Her father loved children to an extent that if he lost his child, he wouldn’t do anything else. Because when I gave birth, he went to get meals for me. After giving birth, I don’t know what happened next. I saw her and felt like someone in a coma. I got back to my sense when I was in the emergency room. I don’t know if you’ve been to the hospital where I gave birth, but there is a long distance between the maternity room and emergency room.

**MODE: Hmm**

W1: I stood around and later sat on my bed. There were other mothers with me that asked me, “where is your child?” I pointed the place where I had left her while I was unconscious. Her father came and asked me, “why are you sitting there? Don’t you have a problem?” He told me that my husband was by the other side, and it was my first time seeing my husband crying. His eyes were red and you could see that he was confused. During that time, I thought that I would give birth on 27^th^ and I gave birth on 15^th^, it means that the date of getting paid had not yet reached. It became necessary for him to go to work and deal with them on finances. Giving birth is a huge burden since when he went to search for money, he lost his camera that was worth 3,000,000Rwf. It surprised me how he went to search for money to cover hospital bills and lost something of that value.

**MODE: You are photographers?**

W1: Yes, it showed me that he was confused. So we entered in the ambulance. I had refused to touch her, and her father was the one that held her. I wasn’t expecting her to live because that was my conviction. I thought that we would reach at the hospital when God has received her soul. When my child was crying, I asked them to stop so that I may get a drink at Nyirangarama. Imagine your child is sick and at the verge of death, and you tell them to park where?

**MODE: At Nyirangarama**

W1: I felt that we would reach there when it’s over. Imagine someone taking cold drinks after giving birth.

**MODE: Why?**

W1: I thought that I would never breastfeed her. I wondered if I drank hot things and got breastmilk, who would I give it to?

**MODE: Yeah**

W1: We reached at CHUK when they had prepared for our arrival. It was my first time reaching at CHUK. We reached there, and first went to the emergency section. When we got there, Dr. Edmond asked, “isn’t the child breathing?” We said yes and he said that there was no need of putting her on oxygen support since she was breathing. I found there other 2 children who were on the same bed, but what I remember is that available beds were very few. That’s something I would say, I didn’t like the beds of CHUK. People could breastfeed their children by placing them on the same bed. At that time it became necessary to get a lamp for the baby, they got one child and put him in another bed since they had fixed him intestines. By then the baby had absorbent cotton and I hadn’t seen it properly.

**MODE: And after that?**

W1: Frankly speaking, I can’t give more details on that part because I don’t know what happened next.

**MODE: Hmm**

W1: I don’t know how they fixed her intestines, it’s her father who would respond to that since I had left because I hadn’t managed to accept it in my mind. In my head, I was feeling that I am getting a punishment. After that, a doctor came, we asked him if that disease was usual. We heard people from our family saying that I’ve been poisoned. The doctor told us that it is a normal disease like any other and told us that in 2007 children who were born with that condition died because they didn’t have treatment for them. He said that he can’t guarantee anything because it is a disease whereby you may bring a child when s/he is still alive and s/he dies or even dies from that place. When a doctor tells you like that, you think that it’s over for the baby. I breastfed the baby after 2 weeks.

**MODE: How was she fed before that time?**

W1: I don’t know, I could see that they injected serum and it sustained her.

**MODE: Why wouldn’t you breastfeed her?**

W1: The issue was that you can’t give a child breastmilk when she has bags.

**MODE: Ooh, I get.**

W1: After spending 3 days in the emergency section, they took her to pediatrics. We found there other mothers who had children. The first thing that discouraged me with this baby was the day I was sitting, I recall that day and get scared, I was sitting and saw ants over her lamp, and moving down to her navel. I felt scared to an extent that I left the place screaming and a nurse came. May she bear with me for saying this, after she took alcohol to cleanse the wound, I told her to leave it because I was going to do it myself. I don’t know how they did it, my child would cry, and I could feel like falling and go out to get some air. After covering the baby, I was extremely happy because it was her first time wearing a cloth. I was happy because I could pamper on her and leave her like that. The day when I put clothes on her, I was very happy. I put clothes on her when they were shifting us to Neo.

**MODE: Where is Neo?**

W1: In Neonatology. They immediately got a room. They requested in Neo to get us a room because in pediatrics they didn’t want children to meet with people from outside. These children are so fragile that if they met with other people, it would have an impact on them. During that time, I felt that my child would not make it, I did not want to talk about children or anything else. But after that, her father, the reason to why it wasn’t challenging for him, he was at CHUK all that time and I spent all those days with her father. I could see that her father had a sensation that things will go well, as for me, I felt that they should let me go because I felt that it was over. The thing that was most challenging was the time when I was told to breastfeed her, I was feeling so happy, but when they told me to fetch breastmilk, I didn’t get it. They even asked for 3 ml and I didn’t get it. They gave me all the nutrients that bring breastmilk and it failed, and it delayed the progress of my child. After that, when I started fetching breastmilk, she failed to defecate. That time they asked that she goes through the scanner to see if her intestines do not have a problem. Saying that they will operate a child like this one, since they had operated 2 babies who died, in the room where we stayed, there were 3 children who died. That was like sitting somewhere and seeing everyone dying, the first one, the second, and so forth, I felt that the next one would be my child. The most challenging thing for me was her tears. Imagine as a parent, a child cries, she is hungry, and you have no breastmilk to give to her. Another challenging thing was when they told me that she can’t have milk. I thought that in that time I hadn’t obtained breastmilk, I would give her milk and she drinks it. They told me that she can’t get flour drinks (soya milk). Then I felt that I was killing my child twice, since for the first time, the reason to why she was born like that was that I hated kids, and the second thing, I wasn’t getting breastmilk. Then I felt that I was killing my child. Since my husband took courses related to medicine, he went to research on what causes the disease, and found that it is caused by alcohol, smoking, and iron pills, and I remembered that I had never taken those pills.

**MODE: Which pills?**

W1: Iron pills that they give to pregnant mothers, when you don’t take them, it’s also one of the causes of giving birth to a child in that condition. I felt scared and said that if my child dies, I’ll be responsible for it at 100%. Since I felt her departure, I would feel like not approaching her, and it was her father who did it. Because I didn’t want to approach her, love her, and lose her later. I felt that it would be better if she left while I don’t know her. The doctor asked that I breastfeed her, there came a doctor called Angelique, and she said that she would breastfeed her. That was the first good thing I experienced, having someone who breastfed my child. I could see other parents holding their children, they could cry and they breastfeed them to calm and sleep, as for me, there was nothing to do about it. I later felt at ease, but feeling at ease, and feeling that your child won’t leave, I don’t know how I can explain it.

**MODE: I understand. Living in anxiety**

W1: Exactly, every night you wake up to wonder if she is still alive. Another thing that discouraged me, I boarded a motorbike and went to town. There came a young mother, I talked to her and told her that there is no need of distancing you from your child to regret that you never spent time with your child. I told her it’s better that we approach them, since we won’t change their life, whether they make it or die, they will still be our first born. Take advantage of the moment while you are still together and start befriending your child. The girl started feeling that she was a mother, I won’t forget that night because they called me and told me to hurry since we met down there. I went hurriedly thinking that my child has got a problem. When I reached there, I found them laying a cloth over her as the child of that woman had passed away. They told me to call her mother, just imagine that you were telling someone to get closer to her child, and then you go back to tell her that her child has died. I felt that it was my mistake because she never approached her and she had no problem. And if she died, she were to accept it. She was a young girl; I don’t know her age but she was young.

**MODE: Her child also had a sickness like the one of yours?**

W1: Yes, after that, I felt like I should tell the doctors to let me go home because I wasn’t doing anything there. The child was not gaining weight, she was not allowed to drink milk, nothing at all. I remember that my husband became mad at me and said, “if you feel discouraged at this hour, your child will leave you.” I wouldn’t feel discouraged at that hour because the child was already 3 weeks. “Why don’t you think that God can bring her to you?” he said. Then it reached a time when she lost weight and the doctors, she has a scar that occurred when the doctors were injecting a syringe, it resulted into a wound and later a scar. Everyday they could inject her like 5-6 syringes, and it reached a time when they shaved her. They even pierced the head with the syringe and pierced the whole body till they thought of piercing the throat. I just imagined them using a vein from the throat of my child, I don’t know if she heard it that they were going to pierce her, she started breastfeeding. When she breastfed, it made me so happy, but after that, there was a problem that she breastfed and vomited. It reached a time when I was seated in the toilet, and said “God, if you want to give me my child, give her to me and I leave. And if you want to take her, I request that you take her without inflicting her with pain. The way I am saying it, I am not begging you, if you want it, take her if you see that it is necessary.” In that time I said it, I didn’t recall that I said that since it was beyond my acceptance level. They could shave one part today, pierce another part the following day. It reached a time when it was so challenging to me but thank God that he came (the father), he knows that I don’t recall a lot of what happened at the hospital.

**MODE: He will tell me that**

W1: Yes, I don’t know how the wound covering went unless he assists me.

**MODE: Yeah**

W1: After that, the first thing I criticize at CHUK is the beds. Yes, they care for the children but a thing they don’t recall is that I am her mother. While I have not rested, there is nothing that I can give to her. I remember that I spent the night awake on the day that I gave birth to her. A mother after giving birth needs rest since a child will need breastmilk from her mother, and a child can’t be sick and you breastfeed her with breastmilk full of stress. I think that they should find a place for them where they can spend a week because the beds were not sufficient for everyone, but a person who came after giving birth should be given a place for resting since it would not be good… for example I have a child and I am fragile. There are various places where I went and came back. The first challenging thing at CHUK was seeing deaths. Seeing someone dead was hard for me to bear. Another thing is how the nurses take care of the children. For example, it is something I would like to ask you and you will respond to me. I am not allowed to enter where the children are, but when the children cry, you are there, right?

**MODE: Hmm**

W1: There are more than 10 children and one nurse. A parent is not allowed to do what?

**MODE: To enter**

W1: If all the children cried at once, would you know where the parents are seated?

**MODE: No**

W1: You get?

**MODE: Hmm**

W1: That’s something they should know. For example if I’m there and the husband is also there. You can say that one person will enter, and in case that person is tired and in need of rest, he/she can get out and someone else can enter because they are not machines and there is no nurse that can take care of 10 children at the same time. There are times when you get there and find that the nurse has gone to sleep. What happened was that another sickness of my child was caused by that. They don’t want me to enter, and the one responsible is not around. There was a time when the leg swole, I don’t know how it’s called.

M1: Hematoma

W1: Yeah

**MODE: It’s called?**

M1: Hematoma. For those who know it, when you inject a syringe and a body part swells, it is called hematoma. They injected the syringe and the serum did not enter. So, the leg of the child swole, and the swelling resulted into this scar.

**MODE: Okay, you may also sit near, we will converse as well.**

W1: Apart from the scar, there were also other side effects on her leg. Her leg is not steady, even when she walks, you see that it’s not steady.

**MODE: It shivers**

W1: Yes, it shakes and it’s not like the other leg. You understand that it is not a side effect that came from the leg, but it came from CHUK. If you asked me a lot, I wouldn’t respond to all of it because I was feeling crazy by then. Even the doctors who were there can tell you that. My mind was not fresh, you may ask other questions this one. He has also come to give me rest, sometimes. I don’t like talking about it.

**MODE: After giving birth, you told me that you were not looking at what happened. For you, after birth, you realized that the child had external intestines, how did you receive that?**

M1: By all means, there is a trauma. Maybe we don’t all perceive things the same way. Another thing is that one needs counselling to hear that it is a curable disease, and the requirements are these. You don’t immediately accept the situation.

**MODE: It is understandable**

M1: Then I got tears in my eyes, and accepting it was not something immediate.

**MODE: What did the doctors say that was the root cause of that situation? What causes the birth of a child with that condition?**

M1: What they told me was that it is a rare disease that most doctors do not know about. It is a new disease that even confuses doctors. What the doctor told me was that the disease occurs when a parent doesn’t take the iron pills, or the parent is below 25 years old, or she took alcohol during her pregnancy. And he stated that it is a disease that cures once treated. You understand that they explain it to you. When they tell you that it is a curable disease, you feel some hope. Then they transferred us to CHUK we went there and I was the one that carried her. She was crying the whole way and one had no way of bathing her or feeding her something. Luckily she was sucking her fingers, but as a parent, it is tough for you to accept that a child will cry and not take anything. So, they said that it was the serum that would feed her. We reached at CHUK and they received her, and she was taken care of. They put her in a bed and raised the intestines in a bag to reduce their size, it was also new. Another thing was that in case she got an infection; the intestines would rot and the child would die. That is also something that one is not grasping, especially that even though the doctors were saying that it is a curable disease, they stated that there is a probability of her recovery and her death.

**MODE: It’s all possible**

M1: Yes

**MODE: Hmm**

M1: Accepting the situation especially when we had no sufficient means or support from families, it was only us.

**MODE: The family abandoned you?**

M1: The family could not understand it, they did not abandon us, they were there. On one side they were wondering if the child will survive. They gave you no hope because they ask you the root cause, and they also don’t understand. Even though you say that you have accepted it, whether she lives it’s okay, whether she dies, it’s okay. But for them, none has hope that she will live. The one who came and found intestines outside would burst into tears, others could go out and leave. That’s how it is, but you say, “she is mine.” If she lives, it’s okay, and if she doesn’t make it, that’s it.

**MODE: What challenges did you face in your one-month stay at CHUK?**

M1: Life was tough at CHUK. First of all, to get there and she spends the night sitting yet she gave birth on that day and she had no rest. Okay, the life of a mother who didn’t have labor is challenging. The life of a mother who is not with her child yet she has given birth is also difficult. Mixing up all those things is also a challenge. Another thing is one’s age; when you give birth to a child with that problem, it becomes hard for you to accept it. Communication also becomes a challenge.

**MODE: Between who and who?**

M1: If you were living in Musanze, you already have connections with people from there, then you get to Kigali where it will require you to find other people, then families get involved. No matter the situation, when you live together without having married, you don’t get support from both sides. That’s the thing. The families do not understand what is happening and it also becomes a challenge. For the life at CHUK, the most challenging thing was the life of the mother with no trauma, who won’t get counselling. It means that if I weren’t besides her, it would have aggravated into a bigger problem.

**MODE: Hmm**

M1: It would have become a bigger problem. It required comforting her and telling her that the child will recover. She even refused to enter and I was the one taking care of the child.

**MODE: Why would she refuse?**

M1: The reason she refused was not because she hated the child, but it was beyond her imagination. Since I’ve been at the hospital and seen things worse than that, I had an experience.

**MODE: You were in difficult moments to take care of the child when it was your first time giving birth, and they hadn’t supported your way of living together. Was there anything good you got from CHUK that pleased you during that time?**

M1: Good things can’t miss to be there. The first thing was collaboration and networking. We gained friends, we gained people. The doctors at CHUK, most of them became my friends, and even now they call to ask us how we are doing, and we tell them that we are okay, that the baby has grown to become a young girl. I send them pictures and they say praise be to God. We had friends and after getting friends, everything you face gives you an experience. We got an experience; though the families were not listening to us, they visited us. They came to CHUK, looked at the baby. They were saying that maybe she won’t survive but they could at least show up. Another thing was that it joined families. Though the families didn’t hear us, they showed up and most of them left the place feeling happy until it reached a time and now we no longer have issues with our families. It means that despite the challenges we encountered at CHUK, there came some solutions out of them.

**MODE: Apart from the solution of families, were they other things that turned out to be good in those problems that you were going through? Like how they say that tough times come carrying solutions.**

M1: The first thing is that we left the place with a healthy child. At CHUK, they helped us and took care of her, they were close to her and we departed when the child was alive. That is something we thank God for. Another thing as I told you, we gained friends; whether doctors, people living near the hospital, and till now they call us to say, “how are you doing? Are you thankful to God, you people from the North?” Till this time, we thank God for that because if it hadn’t been that we wouldn’t have known them. You understand that it is something that I thank God for.

**MODE: Okay. After you and your child were discharged from the hospital, what plan did they give you for taking care of the child and how did you follow it?**

M1: They had given us a plan of taking the child for wound covering at Ruhengeri hospital, they gave us a doctor in charge of us, till now, he has taken care of us. We left CHUK and he fell sick, we spent a week at the hospital.

**MODE: She had suffered from what?**

M1: It’s still the same issue, it was an issue of blood infection and hemoglobin was getting lower. It became necessary that we go there, we went there for wound covering, they looked at her and said, “this child is not doing well.” They hospitalized us and we spent there a week. He took care of us, and after discharged from the hospital, we kept going there for wound covering as it was planned, we could there after one or two weeks. Another thing, he helped us and said that when things are like this, here is what you have to do.

**MODE: When things are like how?**

M1: There are times when they tell you that when you see that the plaster is dirty, you should remove it. That was because they had covered the whole belly. He said that you can get a plaster, it is not necessary to get to the hospital, and returning there would be after two weeks. A month and a half later, she had recovered since he had shown us how to take care of her, and after a month and half, the child had recovered fully, and they removed the plaster. We started putting her in basin for a bath like it’s done normally.

**MODE: Apart from wound covering, was there anything else that they told you about taking care of her? They only told you to take her for covering? Those were the only instructions you received?**

M1: The instructions were there; it was that we have to take her to the doctor for a check-in. The doctor was the one who said that after covering, we can have these test. Like the issue of hemoglobin and … unless I first check

**MODE: No, you can even explain it in Kinyarwanda**

M1: We could give the required blood tests to see if hemoglobin is in good standing, or to see if she has a blood infection, they could check on all those things. That was the main thing that took us to the hospital. The most important thing was to see if she has no problem with regards to blood, or her intestines. The first thing he asked was, “does the child defecate well?” and you could tell him. “Does the child eat well and breastfeed well?”, he asked and we could explain to him. The explanations we gave helped in his decision to choose the tests that we make.

**MODE: Okay, what do you think about the medical assistance/treatment that your child received?**

M1: What I think?

**MODE: Yeah**

M1: What I think is that they took care of the child, they did all they were required, and I can’t condemn them anything. The doctors utilized their knowledge and took care of her.

**MODE: Okay, was there an emergency situation that made you seek immediate medical assistance from the hospital when you had returned at home? Like the child falling sick and you take her to the hospital, or any urgent situation.**

M1: There was no emergency situation because when I got here, I bought a thermometer. I followed her temperatures day by day. I could say that this is a normal temperature. There was no emergency that made me take her at the hospital apart from going to the hospital for wound covering as I told you, when we reached there and found that the temperatures are rising. It became necessary to hospitalize her, otherwise there was no emergency.

**MODE: She never had any other problem?**

M1: She never had any other problem; she grew up well.

**MODE: Till this hour, she doesn’t face any complications of falling sick as a result of…?**

M1: No, it is just normal diseases that affect children like flu, cough, otherwise she never suffered from any other disease caused by the condition she was born with.

**MODE: You commended the services of CHUK apart from the issue of beds. When you set appointments to meet Dr. Emmanuel, what would you say about the services that he gave you?**

M1: The services from Dr. Emmanuel were excellent. Even when it was necessary to… okay, there were times when we followed the plan given by CHUK, and when we got there, he stated that when you reach there, you first start from the health center, and the health center gives you a transfer, and you bring the transfer here. Then you start getting treatment as a new patient. He helped us during that time and gave us appointments that we used to access the hospital.

**MODE: Okay, proceeding on what we were discussing, Etoile recovered, you brought her at home and started taking care of her just like any other normal child. How was the experience of living with her at home from the moment she recovered till now?**

M1: In the life of a parent, a child’s growth becomes more joyful. You can see her how joyful she is right now.

**MODE: Hmm, can she feed herself?**

M1: No, we still feed her. She grew up making herself mature, you see that in everything she wants to act mature. You don’t tell her anything, and what you tell her, she responds to you, and it is really fun.

**MODE: She is not a burden?**

M1: She is not a burden at all.

**MODE: Can you tell me about the financial challenges that you faced while taking care of Etoile after her recovery?**

M1: It is understandable that funds became a challenge especially that a few days after leaving the hospital, COVID came in. I can’t say that I have a high paying job especially that during those days I was studying but I had to stop it.

**MODE: What do you study?**

M1: I studied Biomedical Laboratory Science

**MODE: Hmm**

M1: So I stopped it. I was in the last year and writing my dissertation, then I stopped it. The job I did in my usual life was photography. During the lockdown, that stopped. There was no photography during that time, and for the weddings, they could say that they will be 30 people. You also understand that the profit we used to get lowered drastically. You understand that finances became a challenge. It ended when some company assets were sold at the market to get a living.

**MODE: You both work together?**

M1: Yes, they could call us the following day and tell us that they want a signpost and we do it, after doing it, we could get some income. In brief, the income was lower compared to the days before. One goes through that, but nowadays it’s okay, things are getting back in shape. I had started working elsewhere, and it is progressing slowly by slowly. In these days, the funds are not a challenge compared to the previous times.

**MODE: I’ll pose this question to you especially since you said that it caused you depression to an extent that you couldn’t manage to look at it. Can you tell me in details about the mental depression that you encountered during the child’s sickness? Or even after that if it continued.**

W1: What I know is that someone who had a depression never knows that, it’s others who know it. It’s this one who may state how he saw because I don’t know. I saw things coming and passing by, I don’t know.

**MODE: How was it?**

W1: I saw myself alive.

M1: As she told you, one can’t have mental depression and immediately know that she has a depression. But if she was someone incapable of cleaning the baby/taking care of her, you understand that the person had depression especially since I saw others we found there, or even those who came after us, I could look at all of them and see that they have left their children, and they don’t want to hear that they have been hospitalized.

**MODE: The mother of the child?**

M1: Yeah. You could see her looking crazy, she wasn’t understanding that she was in a hospital. But you wouldn’t condemn her for that, such things happen and she is not the first one to experience it. But the following day, she felt that she was part of the hospital life, and she accepted it. The intestines of the child were fixed back in the belly, and they closed it. They put bandage and she (mother) confirmed that she’ll go to see the baby. They later initiated the program of clothing the baby, since that time she wore a pamper only, and in a light for warmth. You were supposed to check if the light in the lamp is too much or too little. You were supposed to wipe him and see if he has defecated/if the pamper is dirty. Either she urinated or she defecated, and you follow up with everything. At CHUK, whenever she urinated/defecated in them, you could remove them. Another thing, for the acid that got out of the lungs, you had to remember pulling as the doctor told you to help the intestines in …

**MODE: How did you pull since I know nothing about it? How was it with the acid?**

M1: She had a probe in her stomach. You could inject a syringe, suck the content out of it, and pour it in the appropriate place they showed us to help the lungs reduce in size and get back in the belly since the intestines had swollen.

**MODE: You told me that the child was born when everything was out including the stomach. You were not here, how did it go? Share with me your experience. Of all the experiences I heard, this one is new since others only had external intestines.**

M1: Yes, only that

**MODE: So what happened to her?**

M1: She was born with external intestines as I told you, then the doctor used a bandage and covered it to prevent it from getting infections. We left when all the organs were outside. When we reached at CHUK, they held them, there is a bag where they place them, and it helps them to return the organs in the belly. The doctor arrived and tried to return the stomach in the belly and put the intestines in the bags and raised them.

**MODE: She told me that she was pregnant, she used to discharge green water.**

M1: That occurred twice. The first time we went to the hospital and they told us… well, we first went to the health center and they treated her, they transferred us to Ruhengeri hospital where she passed through the echography and said that the child has no problem, the child is playful, you may go home. We went home. She kept experiencing pain, then we went back to the health center.

**MODE: The mother was having pain?**

M1: Yes, we went back to the health center and they gave her pills, they discharged us and we went home. The second time when it happened, we went to the health center and they transferred us, when we reached at the hospital, they said that she has not yet gone in labor, but the day of delivery had come. We spent the night there and in the morning around 7:30, that’s when she gave birth. So, the child was born with external intestines and an external stomach, but they told us that they are going to transfer us to CHUK, and they explained to us about that disease, how it cures, and we went to CHUK.

**MODE: Okay, thank you so much. Comparing this to pregnant people, they say, “when I give birth to a child, I will treat her like this,” all those things that mothers/ anyone else thinks while thinking about the baby in the womb. What was the most challenging in the difference between what you expected and the reality? Where was it challenging?**

M1: Where we found it more challenging, it was challenging everywhere, buying clothes and planning that you will put her in that cloth after giving birth to her, make her wear these clothes, and go home, buy something to celebrate, right?

**MODE: Hmm**

M1: That’s not how it went, because our expectations did not meet the reality, instead it all ended from there. There are times when you say, I have my 30,000Rwf and it will take us to the hospital and bring us back. I will pay 5,000Rwf or 10,000Rwf on insurance, when I reach at home I’ll cook porridge for her, cook matooke for her, and buy a drink. In that time it changed, they tell you that here you are required to have this, the materials we have used are these and those, and the bills are these. For you to get a transfer, it will require this. In brief, it confuses you. If it was someone who owed you 5,000Rwf, you pour all your angriness on them when it wasn’t necessary, and if you think that someone can give you 5,000Rwf, you can call them and ask, “can you give me 5,000Rwf?” You even call others to let them know that you gave birth but that it went like this. You understand that your calculations and expectations differ in that moment.

**MODE: On the other side, did it affect your social life? For the people who heard that she was born with external intestines, what could they say? In that whole scenario, what happened?**

M1: In that whole scenario, the public was on another level, let me say it like that. Someone tells you, “You are foolish, they poisoned you”, that’s the first one. The second says, “aah! There might be enemies of their family.” Everyone gives a different comment, sometimes when you get involved in it, it ends up destroying your relationships and your home. All those things affect you. The rumors affect you, your relationship with them changes, all those things can’t miss to happen.

**MODE: Did it cause a dispute with anyone?**

M1: Yes, you have a dispute with some of them because for example when we left CHUK , they said that none is allowed to visit/carry the child. At the hospital, they will do that. You take care of the child and stay in charge of that. You also know their neighbors; everyone wants to know what you gave birth to. They are like, “how is it? We heard that you gave birth to a child with external intestines, how is it? Their curiosity and all those things, the person who will come to visit you, you will tell her to look at a child from a distance and not carry him. That is also a misunderstanding. It caused a dispute between me and many people, but it ended when we lived the way they wanted us to live. Her health obliged us to do that and there was nothing to do about it. A person who will come to your house and find you wearing a mask while carrying her, since we wore the mask before Covid came. The instructions at CHUK were that we were to carry our child wearing a mask to prevent ourselves from contracting any diseases to her. A person who comes to your house and finds you wearing masks will think that you are arrogant. If you tell him/her that you won’t carry the child, s/he will call it pride. Everyone came with his/her comments basing on his/her will. But on the other side, we had a child covered with plaster and there was nothing else to do. It became necessary that we dispute with our neighbors and sometimes we could shift to new places because of all those things.

**MODE: Okay, thank you. For the relationship between both of you, did it alter your relationship? Whether in a positive or negative way, you are allowed to share it. How was your relationship after all those experiences with Etoile?**

M1: Well, there were good and bad things, right?

**MODE: Yeah.**

M1: Because if the expectations do not meet the reality, then quarrels arise, but the challenge should be, how do you live after the disputes? Because sometimes she will quarrel at you, and if you are capable of the same thing, you will insult her, and realize that it is turning to a fight when it wasn’t necessary.

**MODE: I don’t want to enter in your private life, but which problem did Etoile face that caused a misunderstanding between both of you?**

M1: What she encountered?

**MODE: Yeah, that raised your anger and caused misunderstandings.**

M1: Even though we quarreled…

**MODE: What was the root cause of that quarreling?**

M1: The quarreling occurred sometimes when she refused to breastfeed the baby, and we could quarrel.

**MODE: Mother of the child, why would you refuse to breastfeed the baby?**

M1: Sometimes she could say that she can’t change the plaster cover.

**MODE: But because she was scared of that?**

M1: Even on her side, it involved all those things because the challenge we faced when the child needed breastmilk, she did not get breastmilk, even 5 ml were not fetched. When we left CHUK, she got the breastmilk in a large quantity to an extent that she breastfeeds the baby a lot, and you find that the baby can’t exhaust it all, yet the other one is screaming that the chest is burning her, and all those things. You understand that challenges are ever there, but you live together through all those things and when she tells you bad words, you know that it wasn’t her intention, and you become patient as you cope with it.

**MODE: Okay, thank you for telling us about the life of your child. How is Etoile doing right now?**

M1: She is a beautiful girl who can feed herself, she makes us happy, even when we are sad, we see in her a purpose of our existence. We have a reason for being happy.

**MODE: Okay, for the meals, does she breastfeed only? She drinks soya milk? She drinks milk from the cow? What does she eat?**

M1: On the side of food, you also know that you already have the answer. On the side of eating, she even eats stones, she has no problem. She eats, breastfeeds and for the milk, expect that she dislikes it, when you give her, she tastes on it.

**MODE: She likes breastfeeding and eating?**

M1: She eats porridge and she loves it more than anything else. You understand that she has no problem.

**MODE:** **Does she feel pain?**

W1: No

**MODE: Or vomiting?**

W1: No

**MODE: Or suffering from diarrhea?**

W1: Not at all

**MODE: Or crying frequently?**

M1: No, when her mother leaves she cries. When she is about to leave her.

**MODE: That’s the only thing that makes her cry?**

M1: That’s what makes her cry.

**MODE: It is not related to the sickness?**

M1: Crying because of sickness or pain no longer happens.

**MODE: How is her growth? The weight? The height? Ever since you left the hospital, what were the changes?**

M1: The change is that we left the hospital and she was vaccinated just like all the other children. She delayed taking them but she got them. And the weight, we left when she measured 2.500kg and now she measures 9.700kg, so you understand that she is good.

**MODE: Ooh, so she has one year?**

M1: One year and 4 months.

**MODE: One year and 4 months.**

W1: Yeah

**MODE: Does she like playing? Is she not a lonely child?**

W1: No, she plays, when she is with other children, you find her happy.

M1: She’s a happy child when she is with other children.

**MODE: Thank you so much. We are nearing the end. Can you tell me something you wish to have known about the health of your child or how to take care of it that was needed before? Like saying that basing on what she went through, if I had known this, I would have done this. Basing on the experience that you had, which information do you wish to have known before it occurred?**

M1: In life, we live with regrets. Had I known, I wouldn’t have done that, right?

**MODE: Yes**

M1: She also says, if I had known, I would have taken those iron pills since they prescribed them and she never took them. When she went to see, she realized the consequences.

**MODE: Do not condemn yourself, there are those who take them and give birth to children with similar conditions.**

M1: And she saw that the child would not have been born with the complication. She said, “had I known, I would have taken them.” But on the other, she should condemn herself. Even the doctor says that if you knew it before, they would have treated her from the womb.

**MODE: How? Aren’t they the ones checking in the womb? Why didn’t they do it?**

W1: It is not visible in the womb.

M1: The challenge is that it is not seen in the echography.

**MODE: What does it take for it to be seen?**

W1: It is at birth.

**MODE: At birth, okay.**

M1: But the doctors from CHUK tell you when you pass through echography, they can realize that the child has a problem. So, we can’t blame ourselves because they also blame themselves.

**MODE: Okay, that is what you wish to have known before? The regret you have of something you didn’t know and wish to have known.**

M1: Maybe if she had taken those pills, it wouldn’t have happened.

**MODE: No, what happened had to happen because there are other mothers who take iron but still face the same issue.**

M1: So there is no need to blame oneself.

**MODE: Yeah**

M1: There is nothing special if it was meant to happen, no need to condemn ourselves.

**MODE: As people who went through this, what can you tell a parent who had a child with a complication similar to that of yours?**

M1: What I would tell the parent is not that much. What I would tell the parent is that it is a curable disease, that’s the first thing, and the doctors would also have told him/her that. The second thing, Rwandans say it, “the one who bears what is rotten licks it.” I don’t know if you will hate that child, leave her, and stop caring for her. Isn’t that child yours? You are the one who gave birth to the child, and you are the one who should take care of her. It means that s/he should love the child. If you say that you will go through the good and bad with your husband, why not your child? Even though it’s beyond your imagination, and you have no hope for the baby’s recovery, you have to remember that she is yours. That’s what I can tell that parent. She is yours, whether alive or dead, the child is still yours.

**MODE: Okay, and for you, what advice would you give to that parent?**

W1: For me to accept the situation, it took me a long time because there were times when I kept constantly checking to see if she is doing well.

**MODE: I didn’t hear that part**

W1: What happened to me is that I could be at the hospital, something could scare me and I could run. That’s something already part of me, but on the other side I found that when it’s something that you can’t change, it hurts until you accept it. When something is beyond your capacity, you just leave it, but it is different since the child is yours. Whether she leaves, or whether God takes her away, she will still be your child. The advice I would give her is to love her the way she is, take advantage of that moment, because I realized that when you love a child, there are certain things it adds to her life. She feels that she is not alone. If we were to condemn her because she was born with an issue of intestines, it would have an impact on her life because you have to love her the way she is and do not abandon her because of that problem. During that time, I felt that I would not carry the baby if one gave her to me.

**MODE: Why?**

W1: I don’t know how to explain it.

**MODE: You were still scared.**

W1: No, it’s not fear, I felt that it was beyond my imagination. I said that we were in the ambulance and requested that we park to buy juice. You understand that this thing was beyond my imagination. Yes, we can all have trauma, but we should remember that this child suffers more than we do. The child is enduring pain that you are not going through. You are worried about your child’s sickness but your child is suffering, and when you ignore her, you are not helping her. In that time, we need to behave like parents. We should be like mothers or fathers because for example there are times, maybe it’s not everyone who gets the chance of being with their husbands to help them because if he weren’t there, I would have thrown the baby at the hospital and left. That’s the reality because it was beyond my imaginations. He is also the father but I carried her for 9 months. Just imagine being pregnant, taking this food because it will be pleasant for the baby, and leaving the desires of your heart to ensure that your child is born healthy, and s/he is born like that. She can’t condemn herself like the way I would do it to myself because I am the one who is supposed to take the medication, you understand that thing. When the husband comes to quarrel with you, he will say that you are the one who destroyed his child. Or for example, your husband may tell you to mind your business and take care of your child, yet in reality, women are weak, and any occurrence may traumatize us. What I can request from those mothers or anyone else, is that everything comes for a reason. Frankly speaking, there is nothing that can happen to me this hour and I feel that it is complicated. My child made me realize that no matter the situation, your blessings follow you. She should not condemn herself and feel that it was because of consuming alcohol because I sat down and realized that I didn’t take medicine. Then I was like, if I take pills, the years will become a challenge, and the 3 years that followed, there was nothing to change about them. The first thing I request them is to accept themselves and forgive themselves because the hardest thing for me was to forgive myself, or to feel that I don’t have any mistake (that was the main challenging thing that traumatized me). I know that most mothers blame themselves and have failed to forgive themselves since there is nothing that they can do about it. They should not feel that they have mistaken, but they should correct what they call mistake.

**MODE: Hmm**

W1: Because if you feel that it was your mistake, maybe you drank beer or took anything else, that won’t change a thing. Instead, find ways to unite with your child, and leave the past, ignore it, and focus on your child. That’s what I can personally say, it was a challenge to me, finding that I can change nothing about it.

**MODE: Okay, thank you so much. This is what I had planned to discuss with you. Thank you for your time, it was not that easy. Before we wind up, I don’t know if you have a question or suggestion before we finish the discussion?**

M1: A suggestion. It would be better to train doctors from the countryside because you find that doctors from the countryside don’t know. These are new things to them. Doctors should share information such that when there is an incident, it should not be new to the doctor. There are many children who die because the doctor who helped you deliver did not know that condition, and their ignorance becomes a barrier. So they should share information, it will save many. Another thing, it would be better to have doctors that study on the disease and know it, such that if someone gives birth in Ruhengeri, they will have a doctor who knows about the disease, who will treat the disease and it heals.

**MODE: Okay**

W1: I also want to add something to what he said about training. For example, the reason to why a disease doesn’t surprise a person, if the baby suffers from cough or anything else, there are things that they tell us a s mothers before delivering, like when you are pregnant, you have to take with you this and that because I realized that a child may be born having cancer. That’s something shocking and scary to a parent because s/he knows that it exists. Or if a child had a heart issue, it is a serious disease, but I kept wondering why the issue of intestines scares most people because it’s something that people don’t know. As you request us to talk to those mothers, it would be better if they knew this thing exists before waiting for our advice after giving birth. Instead, they should tell it to them before they give birth.

**MODE: Yeah, the possibilities that may arise**

W1: They should feel that when it happens, it is not something unusual. I can give you an example of one who said that it’s witchcraft. There is one who may believe that it’s poisoning and go straight to someone they disagree with in normal life.

M1: It happened at CHUK.

**MODE: How did it go?**

M1: There was a mother that came with a child with external intestines. Her mother and her mother-in-law were telling her that she was poisoned, and they went back to avenge, as each one said, “wait, this person will see me.” They were betting on other people saying, “this person will see me, I know she is the one.” Why? Because there are hatred cases in neighborhoods and one may think that it’s those people who took away our child. And you say, “wait, I will show them,” yet it is a curable disease. They even told that lady, “you are foolish, what will the hospital do about it? We should have consulted witchdoctors who know what it is.” And they added, “do they treat witchcraft at the hospital?” She came to the hospital knowing that they will rescue her, since the area where she had given birth, they had transferred her to the hospital. When she reached there, they started saying, “you are stupid,” which means that if she knew this before, it wouldn’t have become a problem. Her husband had gone somewhere else, and she called her but it didn’t work because he had cut off the communication. It means that if these were known things, it wouldn’t be that hard. It can change people’s perception in the society to an extent that if something like that happened, they would not think that it’s witchcraft. I think that they should consider this and put much more effort.

**MODE:** **Is there anything else?**

W1: That’s what I was saying, something becomes good when you prepare for it. My husband said what I was about to say. They should try to convince the mothers by teaching them that this disease exists. Just like the way they sensitize us to go and get vaccinated, they should also teach people that this disease exists such that when you deliver the baby, that thing does not become a surprise to you. You should feel that it’s something existent, and that’s where I think they should put more effort. Experiencing things after giving birth is not easy like getting advice when it’s still early.

**MODE: Hmm, yes. Thank you so much.**

W1: Thank you as well.

**MODE: Have a nice day.**

W1: Hmm
