# Supplementary material for: Exploring the lived experiences of parents caring for infants with gastroschisis in Rwanda: The untold story
Source: PLOS Glob Public Health. 2022 Jun 15;2(6):e0000439. doi: 10.1371/journal.pgph.0000439 (PMC10021215; doi:10.1371/journal.pgph.0000439)
Supplement: S1 Data — (ZIP) [file pgph.0000439.s002.zip › S1_Data/S3_Text.docx]

**BB 11 English Transcript**

F: How are you?

P: I am good , how are you too?

F: Yes, we are alright. My name is Gentille and I am calling you from Kigali

P: Yes

F: Hmmm, I am working with and for Dr. Samuel Kidane and Dr. Semay Desta Shamebo , Master of Science in Global Health Delivery students, University of Global Health Equity.

P: ooh yes,

F: This research project is exploring the lived experiences of parents of infants who received hospital-based gastroschisis care) because you meet the requirements for inclusion into the study and can offer unique information regarding the question at hand. Before accepting to join this project, you must understand and take into consideration the contents of this form, since it contains important information to assist you in deciding whether to

participate or not.

So before you agree to participate in this research, it is important that you understand the contents of this article, which contains important information that will help you decide whether to accept or refuse to participate in this research.

Therefore, This project is being conducted as a core requirement for the Master of Science in Global Health Delivery at the University of Global Health Equity. The project has received required ethical approval from UGHE and complies with international ethical standards for research to be carried out in Rwanda. Additional permissions have also been obtained from the CHUK and the IRB of

the University of Global Health Equity.

F: This article contains important information that may help you choose whether or not to participate in this study. Take enough time to understand the content of this post. Seek advise from family and friends if necessary. If you have any questions about this project, or this article do not hesitate to ask. If you agree to participate, you will sign this form and be given a copy for your records. Since we are using the phone, you do not immediately sign it, but we will send a copy of it and sign it, but now you will let me know if I can record our discussion. So if you agree to participate in this study you will come and tell me, I will come and ask you.

F: That is to say, the goal of this project that I have been explaining to you, that is, we are looking for information so that CHUK can increase it, so that it can improve the service delivery of parents with children born with this disability. Any information you share with us is important and, we will use all the information you share to us, we will send it to CHUK doctors, but we will not tell them your name and profile.

*P: hmmmmm,*

*Q: Maybe you could say what’s the point in this? Yes, we do not promise immediate benefits but by participating in this study, the information you will provide will be used to improve the services provided in the Department of Pediatric Surgery at Kigali University Hospital (CHUK). Also, this information will help other parents, and caregivers who face this disease in their children.*

*P: hmmmm*

*F: So the other thing I would say is, would you be wondering what would happen if you wanted to stop this participation? surely, we may have started discussing, but feel free to stop it if you want. Yes!*

*P: UHH HMMM, No no problem*

*F: Hmmmm, You can even ask me if you ever have a problem*

*who should you call when you have a concern?*

*P: hmmm*

*Q: If you want, I will give you the numbers of Dr Samuel Kidane and Dr Semay, and their emails also and I will give them to you to solve the problem so that you will know who to ask.*

*P: Hmmmm*

*F; Ahhhh, so, this document to participate in this study means that you allow me to record this discussion so that the information you give us can be used.*

*P: Hmmmm, no problem*

*F: Did you agree? Did your husband agree?*

*P: hmmm*

*F: Yes, that means you understand the content of this post*

*P; hmmmm, yes*

*F: You have had the opportunity to ask questions and have been satisfied with the answers you have received*

*P: hmmm, yes*

*Q: When necessary, you the time to talk to your husband or someone else to help you decide whether to not participate or to participate.*

*P: Hmmmm, yes*

*F: You also agreed to record this interview*

*P: Hmmmm, yes*

*F: Thank you very much, thank you, now we can start*

*As I kept telling you that the purpose of this questionnaire is to find out how your child was being treated at the hospital and how you and your husband have taken care of your child. We would like to use this information to help CHUK Hospital to make a difference in the service they provide, but I will continue to say that we will not disclose your name or profile to anyone.*

*P; hmmmm*

*Q: Did you have a problem before we started?*

*P: No problem*

*F: Yes thank you very much*

*F: EEh, so the first question, let me start with what was your experience at CHUK whether you or your husband , tell me when you entered, how many days, you and your child stayed in the hospital.*

*P: That is to say, we reach CHUK*

*F: Yes*

*P: When we get to CHUK they welcomed us, they welcome us, we had no problem, they gave us the service we needed, they are just good people.*

*F: really?*

*P: They treated us well, however, there were doctors who were there, you see, they didn't do things well.*

*F: Yes*

*P: It was as if some of the nurses were not really able to help children from night to the morning*

*F; ooooo, hmmmm*

*P: Also there are others who did it very well.*

*Q: So when did you get to the hospital?*

*P: We went to the hospital on the fith*

*F; of which month?*

*P: May*

*Q: which year please?*

*P: The year is twenty-twenty-one*

*F: Eehh, Is this the year?*

*P: hmmm, it's this year.*

*Q: Fith , May?*

*Q: Yes*

*F: Hmmm, so does your baby have one month?*

*Q: Come on*

*F: eeehh, right!*

*P: Yes, And now we have an appointment on the 28th*

*Q: What? And now you are in Nyamasheke!*

*P: hmmm yes we are Nyamasheke*

*F: Oh Jesus, how wonderful!*

*F: Now tell me in detail the service they gave you, you told me they gave you the best service, but you really were coming from far, see?*

*P: Hmmmm,*

*Q: Tell me how it happened, you gave birth, you see you gave birth to a baby, who had intestine outside, how did it happen to get out of the initial hospital where was it? How did you find yourself in CHUK?*

*P: So Normally, how it started, they went to a near health center first*

*F: Hmmmmm,*

*P: When we got there, the mother was transferred to Kibogora District Hospital.*

*F: Why?*

*P: They saw the baby was short of breath, from the womb*

*F: Yes*

*P: Then, they sent us to the district hospital, there, nurses followed it up with us, when they also worked hard, they helped us, and my wife ended up giving natural birth, she gave birth*

*F: Eeeh, she gave birth well!*

*P: It' was about 8 o'clock in the morning*

*F: hmmmm*

*P (Husband): So, nurses were also also surprised, they were also very surprised that this was also the first time this has happened, they were so shocked that they immediately took a picture of the child and sent it to CHUK,*

*F: hmmmmmm*

*P: CHUK said that they could help the child, they added that such cases happen. Shortly, they sent us to CHUK, and we went up,*

*F: Hmmm*

*P: It was just hard, it was hard because you see we got to CHUK really and the doctors who looked at him were scared because you see we spent like seven hours on the way from Nyamasheke to CHUK*

*F: Did you come in an ambulance or in a regular car?*

*Q: Yes, we came in an ambulance, we arrived there, a tired mother and she had to take maternity care.*

*F: hmmmmmm*

*P:They surely cared about us but they were scared because they had little hope because they were telling us that even the intestines were already infected.*

*F: hmmmm of course, did you come and covering the intestine of the baby in the car ? how did you do?*

*P: Ok that is to say that they took things if they were like ice if it was something else, I am not sure, they first covered it up and then, they put his clothes.*

*F: Yes*

*P: we brought him in, really covered but, he also had so many intestines that we were afraid that ,,, we would come and see if he was dead because we saw it as something new.*

*F: Hmmm*

*P:whn we arrived there, they really took good care of us, honestly speaking, there are only good doctors, there are doctors who I really liked and myself, who really care about me and I feel like I liked them so much.*

*F: hmmmm*

*Q: It is because they really helped us because by the grace of God they helped us until we saw the child recovering slowly....,*

*F: Hmmmm*

*Q: So time went on until they came and told us that it was time to leave the hospital and go home, come back for an appointment.*

*F: hmmmmm*

*Q: That's right*

*F: Hmmmm, so husanda, you were you with your wif eat the hospital!*

*P: Yeah, I was with her, and she was really terribly not okay. I had to care both, the baby and the mother*

*F: hmmmm*

*P: We spent three weeks in there, we spent three weeks, three weeks I was the one who cared for the sick baby.*

*F: Yes, she was also in a bad condition*

*P: hmmmm, she was also not okay because you see ?*

*she gave birth and she had to walk for seven hours right after the birth! , it was not easy.*

*F: In those seven hours, was she able to get what to eat please? like a new mother, she had to eat what was it like, when you guys got to CHUK what was it like ??*

*P: Okay, that is, when she couldn't eat, it was difficult for her because she was depressed she couldn't eat at all.*

*F: Yes*

*P: That's when we started to accept ourselves and otherwise we would go down and say maybe it was a sin we committed against God maybe no one would know, until we got there we found kids with case like this and we said no one knew there was still hope*

*F: hmmm, thank you very much, so let me talk to the lady but all of you I'm going to ask you this question, you mentioned that, you said that you guys thought it was a sin you committed against God, the lady is in the doctor's office giving birth, she's going to see that she has a baby with an intestine outside what did she think? How did she feel about it? How did she react? How did she react?*

*P (Husband): That is to say, for me*

*F; hmmm, for you yes yes for you*

*P: That is to say, for me, the rest of the family, the told me this happened to my wife and babay,and I immediately fell to the ground, that is, I went into a coma for like an hour long*

*F; oooh*

*P: I came back to normal after a while and I tried to be strong like any other man, but I guess, I could not. it was hard for me to accept this at that time*

*F: Hmmm, of course*

*P: Hmmmm,*

*F: So you joined your wife and child at the hospital*

*P: Yeah, when I got there I looked at the baby I thought that it was over I didn’t understand he would be alive,*

*P: I started by telling myself God, if you know that he will not live, then you can please take him earlier instead of letting him in pain*

*P: Now when we got to CHUK, there were places where I could see nurses injecting him, and there were doctors who couldn't find veins from where to inject him for about three hours without finding the veins.*

*F: oooo*

*P: there would come and find veins in a very short time. some doctors were incompetent, someone came in two minutes and they had already found where to inject babies .*

*F: Hmmmmm*

*P: Hmmm*

*F: Yes*

*Q: Only God made this*

*F: And how did your wife react when she saw him with intestine outside?*

*P: Well, it was hard to accept it, I just gave birth and now and looked at it and they told me that he really can live or die because so many children are born and die, they say there was a 50/50 chance to live.*

*F: Hmmmm*

*P: At the same time, they took the bay into the machine, and it surely hard to accept this*

*F: Hmmm*

*Q: Now that we have reached CHUK, we find that other children are just like that and we realized that a lot of children would go and die right after having their intestine fixed. that was really hard to accept this , but God has done wonders for us so.*

*F: oooo Hmmmm*

*So what did the doctors tell you about the disease right away?*

*P: They never told me more than just telling me that it just happened, that it was something that happened that way.*

*P: They told us we had to take good care of him and keep him very clean*

*F: Hygiene?*

*P: Yes, cleanliness, because these children need to be cleaned, because even when they are healed, they are more likely to be killed by infections and not to be exposed to dirtiness.*

*F: eeh,*

*Q: So if you don't breathe in the spirits of different people, you will be very clean to them.*

*Q: So what does it mean to keep a baby clean?*

*P: To keep her clean is to put on nice clothes, wash her and you are also clean, hmmmm*

*F; Hmm yes, thank you*

*P: Hmmm*

*Q: So when did they say goodbye?*

*P: When is God !!! They said goodbye to us,*

*F:your husband can remind you(Her husband was the one I was saying to remind his wife)*

*P: I don't remember either (Ruth's husband)*

*Q: Now that you have been admitted to the hospital, you were gave birth on the fifth ?*

*Q: Yes, on the fifth May,*

*F: You've been in the hospital for three weeks*

*P: So we spent a month in the hospital !!*

*F: You were discharged in June?*

*P: yes we left the hospital in June*

*P: yes we have been in the hospital for a month.*

*Q: So for the services they gave you at the doctor, or for the health services they gave you, what did you appreciate and what did you not appreciate? How did they react ??*

*P: God, the doctors there are good, they care for the children, even if they might die, but they do all thier best.*

*F: Yes?*

*P: They really try their very best.*

*Q: So what was your favorite service? /*

*P: God I really liked all the service because the doctors care for babies. Before touching them, they wash their hands as well.*

*F: Hmmmmm*

*P: Hmm*

*F:: And what was the service your husband appreciated less? Or what was one good thing they did that he still remembers?*

*P: The good thing they did for me was when the baby was crying and the baby was crying.[ laughing in the background while her husband speaks]. there was a nice nurse He reached out to all the child and calmed him down, I wish he was the one to work in that place alone*

*F: Yes*

*P: Yes, that is something I will never forget in life.*

*F: Yes hhhhehe, tell me something is done for you or a service that was given to you and you did not like it.*

*P: The only thing I really see as a problem is that they should put the effort about finidn qualified nurses to inject those babies. They have to appoint people in charge to monitor them so that at times you see them coming as a doctor with issues in finding veins for injecting babies that they don’t really understand.*

*F: Hmmm*

*P: because it seems like you come and find out that four hours ago he was still tying to inject the baby because he didn't understand it and you found out that maybe another doctor came in at least thirty minutes and he saw the vein and inject the baby*

*F: Yes, that's right, vein stuff.*

*P: hmmmm*

*Q: Has there ever been a time when you felt that your child needed urgent help?*

*P: Hmmm, it happened ...*

*F: There, explain it to us in depth*

*P: There was a time when he was short of breath, so it was necessary to take care of him whether he was at level one. he was put at level two or level three. They really took care of him, they have done everything, I saw.*

*F: Hmmm*

*P: And once he was vomiting, and they took good care of him, and in case he got fever, they gave us pills to pass from anus, in short , they just helped a lot*

*F: so tell me when you got home and when you gott home, when you were discharged you and; baby and you see he's still small, tell me what it's like to be home with Rene.*

*P: It Feels good, really, hmmmmmm, he's fine, he still has an injury on the jaundice, I'm just cover him a lot*

*F: Hmmmm*

*P he has no problem really; you see he has no other problem*

*F: Hmmmm, do you feel safe?*

*P: So I feel safe because I used to look at the intestinesoutside and wondering how he was going to live, whichwas really hard for me to think and hope that things will work out for the baby I feel better now*

*P: He is our first born*

*F; Yes? is he the first? so how do you see your husband reacting?*

*Q: Did he accept this challenge as he saw it?*

*P: yes no problem*

*F; So tell me about what challenged you, you see the baby was in born in Rusizi, both you and your husband*

*had to live togather in the hospital, you were in the hospital for three weeks, tell me the hardn times yo experienced, maybe about your finances aand so on what was it like to be able to keep up with your financial situation?*

*P: Poverty really hit us,*

*F; How was poverty?*

*P: Because you see when babies are in there, they have to wear just Pampers*

*F; Children?*

*P: yes, children*

*F: yes!*

*P; So you see we were all gone and no one was home to make money so you see it was hard really*

*F: So where did you get the money that you were all in the hospital?*

*P: There was really time, like a brother/friend sometimes sent me a thousand because no one was left at home to make money, which is really a problem*

*F: Hmmm, hmmm*

*Q: And we were not prepared since we never imagined going to CHUK when we were waiting for the birth of our baby*

*F: and, and what has been your daily routine before going to CHUK?*

*P: So we didn't have a job, we just had a bad time while I was pregnant I was just eating and sleeping.*

*Q: And the man?*

*P: The man used to go to look for something to do to earn some money on a daily basis, He would find nothing sometimes and come back empty*

*F: When he really took care of you, everything else was interrupted.*

*P: Hmm, when he cared for me, when he cared for us everything stopped because we used to eat once he finds want to eat for us*

*F; hmm, did not you starve in CHUK?*

*P: Well, we used to get food volunteers who bring food at the hospital... we were able to get that food*

*F: Yes*

*P: Because some philanthropists come and give food, yes indeed*

*F: Tell me about your mental state, have you ever felt mentally or physically disturbed?*

*Q: As soon as we saw him, we were shocked, because we were wondering if he would really be alive and he was the first child!*

*F: the first!*

*P: We prayed God has help us have the boy alive even if he would live aleast with a disability?*

*F; Hmmmmmm*

*P: That's right*

*F; Hmmm, can you ask your husband if he faced any form of trauma as well?*

*Q: There was no shortage of trauma, there was trauma, there was depression, and a week later we were starting to accept it because we have already realized that it happenes.*

*F: Yes*

*P: because some people came to see us and said after being discharged and they gave us testimonies telling us that also their children had GS but they were healed by then*

*F: Hmmm*

*P: That's where we started to see that everything is possible and we start to take over and life came back. in short, we experienced the trauma*

*F; Hmmmm, hmmm now by comparison, by comparison, you see you have a baby of a month. How do you compare the child to the way you were when you were pregnant? What do you think they have in common?*

*P: Raising him is difficult, because we thought it didn't come this way!*

*F: Hmmm*

*P: you see before she gave birth, was my wife had a miscarriage with the first pregnancy*

*F; Your wife!*

*P: Yes, my wife; Now that she was pregnant with this baby, they told her that the uterus was not in the normal place( was close to the outside), that she should sit down and lie down often. So that is how she had finished all the seven months of her pragnancy,*

*F: Yes*

*Q: So when I realized that the due day was close, I thought things were going to be well and maybe she would help me with the tasks at home and make the home a better place.*

*F: Yes, I understand*

*F: So the way I thought it would go did not work. Things happened differently,. instead I even sold what we really had to take care of the child the mother and that is how life really changed.*

*F: Hmmmm I understand it wasn't an easy time. so,*

*P: [seems adding something]*

*F: There was something else you were going to add,*

*P: nothing more, no problem*

*F: So you see*

*P: Hmmm*

*F: Do you think that the fact of you have given birth to a child with a disability and that it takes a lot of effort to get them to CHUK has caused any consequences or do you see any changes in your relationship with your wife?*

*P: oh nothing at all, nothing in our relationship has changed at all we have accepted this. We accepted it as something that can happen to everyone, it was like an epidemic because we are not the only ones.*

*P: No one has wronged anyone by saying that what happened was your fault.*

*P: No one really!*

*F: How did your husband take this? [asking the woman to comment on this question as well)*

*P: Hmmm, no problem*

*F; so tell me how your parents, siblings, neighbors how society is with you after those things?*

*P: there are people who have accepted they really wished us well. However, they are afraid since it is the first time they have seen this.*

*F: Hmmm*

*P: but a big number took time to comfort and pray for us*

*F: Didn't they tell you that maybe it was witchcraft?*

*P: Just as soon as I gave birth to him, I myself thought it was a poison and thought people might have poisoned me, but when I got to CHUK, I found other people with the same problem.*

*F: Hmmmmmm, you knew it was not witchcraft then.*

*P: When we asked the doctor, he told us that this was happening in the past and that they just didn't have the ability to help them, so they let them go home and died.*

*F: ooo, so thank you for sharing with me your life experience, yeah, there was another question I had, so now allow me to take you back to the baby's life, what does the baby look like now?*

*F: You see the baby is a month old*

*F: hhhhh?*

*F: Do you know how much weight a baby weighs at month? and compared to other children how is his weight? Tell me about his life*

*P: He breastfeeds, I see he has no problem, and even looking at it I see it increasing slowly.*

*F: How much does he weigh?*

*P: He was born with 36kg.*

*F: Now you don't know the weight he has?*

*P: No, I do not know how much he weighs. He lost weights when he was born*

*F: hmmm*

*P: Because he was fed by serums*

*F: Now you don't know the weight he has?*

*P: Hmmm! No!*

*F: And your husband?*

*P: I don't know either.*

*P: I just went for a test a few days ago and they told me the baby had three but he had a few past weeks been not breastfeeding so well, so now I don’t know how much weight se has now.*

*F: So can you tell me something you wanted to know about your child's health or the care that was needed? Has anything like that ever grown in your mind?*

*P: I asked because I went through a six-month-old pregnancy ecology and they never told me that the baby had a problem, they told me that the baby was healthy and healthy and had no problems.*

*F: Hmmmm so what would you say to a parent, this is a problem for both of you, anyway, what would you tell a parent if she had a child with a problem like yours?*

*P: We can tell him to be strong, and he prays to God himself, and God really helps him for the good fortune of healing because it is a cured disease. Because there are others who have really came back to CHUK in control, so they also shared their testimonies with us and there was no problem with it. They tell us, "Look at that!" The boy who was born was like that and weighed one kg and they said to us, do you see a problem with him? Let's see if he has any problems. No problem at all*

*Q: So is there any like five-year-old you saw was born with GS problem?*

*P: I saw her myself! After all, there grew like any other kids and healed!*

*F: Heeeee! So what can you say to those who claim that GS is all about which craft or something evil?*

*P: No, don't think about it because that's what happened because God creates as He really wants and God creates as his wills as he allows people with other disabilities.*

*F: So what helped you take care of yourself and your husband, what do you think you had or what did you use to be able to take care of your yourself or something that kept you moving forward in the journey to take care of your baby boy? See? you told me that up today he is without any other problems.*

*P: Oh I see it’s all about praying*

*F: Hmm, Prayer?*

*P: Yes, it is. Praying God helped us*

*F: Is there anything else?*

*P: It is to worship God and keep you believing and have hope that he will really heal*

*F: Does your husband have anything else to say?*

*P: One more thing I would really add is that even the doctors really kept us going and boosted our confidence.*

*F: Hmmm*

*P: Doctors also reassured us and took care of us and told us that the babies born with this case were cured. Includes a lot of people testified to us that most of the children they received had the same case as ours and healed, hmmm that was what kept us going plus praying.*

*F: Yes*

*Q: Hmm*

*Q: When is your appointment for Rene now?*

*Q: We'll be back to Kigali soon. on the 28th*

*F: You mean on the 28th of this month??*

*P: yes, you are right!*

*F; What are you going to do there?*

*P: It's a control, they're going to look at the situation of the baby now, any progress or anything changed, they want to check that*

*F; yes, yes*

*F: eeeh so is there a problem you want to say or something you want to add to this conversation?*

*P: Well, the problem is that the baby's missing vaccination. We came to know that there was a needles of vaccines that were supposed to be given at birth so that's the hardest thing for me, the hardest thing for me. I also asked a nurse that I found at the health center and he didn't tell me anything that could help us with this issue. So I don’t know what advice you would give us maybe.*

*F: That is to say, when he was born, there was no vaccine, and when he was born, there was no drop of vaccine in his mouth and there was another vaccine that is put it in his hand, and he didn't get anything?*

*P: That is true, He didn't get anything because everyone saw him and they all fell into a trance and some of them ran away, yes some of them even started running away.*

*F; yes, that is to say that to date your child has not yet been vaccinated?*

*P: Yes, no any vaccine. I asked a doctor if all the needles they were injecting were including vaccine for him, and the doctor told me no! because if they had vaccinated him, they would have given us a vaccination form.*

*F: That means you have no form!*

*P: We have no form*

*F: That's the problem, I'm sorry!*

*P: Please ask this question on our behalf on how we can do and on how we can start with the vaccines of our son. When we go to the health center they don't give us any advice and we ask this often but they don't help us.*

*F: Yes, does the woman have any other problems?*

*P: No nothing, nothing*

*F: surely, I will follow up on your questions by asking indeed these questions are valid since every child should be given vaccines. I will follow up to find out why, and how to do.*

*P: We have another question: My wife thinks it is now time to start carrying our baby on the back as all women in Rwanda do. However, I always refuse her to do so. would you advice us to carry the baby on the back?*

*F: Okay, I saved this number for you, I'll ask your doctor, Edmond, do you remember him?*

*P: Yes, I remember him very much*

*F: So let's find out what to do, what to do if the child is questioned about being carried on the back and*

*the question about vaccination,*

*P: Thank you very much*

*F: Keep checking if you have any other questions please*

*P: No other problem really, the other problem is the eye problem. He had an eye disease*

*F: when he was born?*

*P: yes, at birth, the child, had an eye disease and was helped to recover but was not fully recovered when we got discharged from the CHUK hospital*

*F: you got the first medicine from CHUK, right?*

*P: Yes*

*P: As he did not cure, we found him another medicine one from a health center over here, but when I look at it I find that it is not the same as the one they had given us.*

*F: Where did you get that first one?*

*P: CHUK, so we refused to give it to him the second medicine because we said it was barbaric, no one would believe it because their medicine is not official and we don't know, so you can ask for us if the medicine they give us would be given to him.*

*F: Do you remember the medicine they gave you?*

*P: No*

*F: Do you see it close enough to tell me the name of the medicine*

*P: No we are not at home I called my wife and we met at a place.*

*F: then you once you are back home, call me back or write me a message and you give me a message or I call you again. Then you will come soon to Kigali, please all this again*

*P: Hmmmm,*

*F: Then you will know what to do.*

*Q: So there is another problem with the coughing and we took him to the health center and they gave him medicine but we refuse to give it to him as we wonder if we can give him the medicine from the health center*

*F: Hmm, we're going to ask, but did you take him to the health center and told doctors that he was born with this problem?*

*P: yes, we did*

*F: eeh no problem then*

*So, if those who work at the health centers are nurses, and they are aware of your child;s history, they reallyh know enough about the medication they prescripted for you. Feel free to give it to the bay. Just follow the instruction so very carefull, hmm?*

*P: That is true, and thank you so very much*

*F: Thank you both for all the time you gave to this discussion, please say hi to Rene for me, and may God keep him safe as he is. Have a beautifl day!*

*P: We equally thank you for the time you gave us, and we wish you a very nice day . thank you so very much!*

*F: Sure, you are all welcome*

*Alright good bye*

Recorder stopped: 00:40:26
