# Supplementary material for: Exploring the lived experiences of parents caring for infants with gastroschisis in Rwanda: The untold story
Source: PLOS Glob Public Health. 2022 Jun 15;2(6):e0000439. doi: 10.1371/journal.pgph.0000439 (PMC10021215; doi:10.1371/journal.pgph.0000439)
Supplement: S1 Data — (ZIP) [file pgph.0000439.s002.zip › S1_Data/S4_Text.docx]

**BB 10 English Transcript**

F: My name is Gentille Dusenge, and I am helping the researchers, Dr Samuel Kidane and Dr Semay Desta Shamebo

P: hmmmm

F: They are both master’s students at the University of Global Health Equity

P: hmmm

F: They are doing this research to explore the lived experiences of parents of infants who received hospital-based gastroschisis care) because you meet the requirements for inclusion into the study and can offer unique information regarding the question at hand. Before accepting to join this project, you must understand and take into consideration the contents of this form, since it contains important information to assist you in deciding whether to participate or not to take part in this research.

P: Hmmmm

F: Hmmmmm, so to add on,

P: hmmmm,

F: and, this project is being conducted as a core requirement for the Master of Science in Global Health Delivery at the University of Global Health Equity.

P: hmmm

F: The project has received required ethical approval from UGHE and complies with international ethical standards for research to be carried out in Rwanda. Additional permissions have also been obtained from the CHUK and the IRB of the University of Global Health Equity.

P: Hmmmmm

So what else can I add, Participation is voluntary, whether you want to participate or not but it is voluntary not coercive

P: hmmmm

F; You could say that I really feel like I don’t want to or I agree.

So we are discussing the purpose of this study, when, or during the interview scheduling process (which will take place initially via telephone before agreeing upon a set time), the research team will discuss / or we are discussing the purpose of the study which is to explore the lived experiences of parents or guardians of infants who received hospital-based GS care in Rwanda. The project will help to describe the parents’ experience on hospital-based GS care for their infants, their perception of the health status of the infants, challenges faced and the health care utilization experience after GS care. The information collected will be used to understand the biosocial aspects of neonatal surgical care and additionally inform the improvement of surgical neonatal care at CHUK. You are being asked to participate in this study because of the unique experience concerning your child’s GS care, both hospital and home based. I hope you spent time at CHUK when your baby was sick

P: Hmmm, we went and stayed at CHUK

F: Yes, yes, so I am asking to voluntarily take part in this study because of the knowledge you have gained through the process of treating your child and taking care of him or her at home. So maybe you would wonder what are the consequences you would have or what would be the consequences of participating in this research?

P: hmmmm

F: You may come to feel uncomfortable, maybe remembering the emotions you had when these things happened to you. Or you may even feel uncomfortable aahh, whenever you feel bad tell me or whenever you feel you have a problem you can tell me to give some time , all the time you might want

P: No, I really have no problem

F; yes you might also be wondering about what is the benefit of participating in this study Although there are no guaranteed direct benefits for your participation in the study, the information collected will assist in the improvement of surgical neonatal care at CHUK and possibly help other parents who share similar experiences as yours..

But you would be given five thousand Rwandan Francs to help you travel to the place where you will meet the researchers.

You may also be wondering if I can stop this conversation whenever you want. yes! If you want to stop in the middle of it you just tell me,

You might tell me like hey, I don't feel comfortable anymore let's just leave it . That's possible!

Any information you may have will be used for research, but your name and profile will be kept confidential and we will not share it with anyone.

P; Hmmm yes

F: Yes. eeh you may have a problem and say like who will I ask among these people? to ask a question or give us a suggestion . Ooh, I will send you phone numbers and / or emails. so that in case you have a problem, you will find a place to ask.

F: eeh thank you so much for giving me the right to talk to you, eeeh you see there were questions you were going to ask me, asking me if my names would be announced on television,

P: Hmmm

F: No, it's not on television. Eeh your names will never be published but the information you provide will be really helpful to find out what they do

P: Hmmm

F: Also You can stop, you can stop participating in this study whenever you feel tired or whenever you choose to

P: Hmmm

F: Also, you would be given 5 thousands Rwandan francs of appreciation that would help you to participate in case it was needed to meet at the field trips where you would meet the researchers . If you allow me to record this interview you would even receive a copy of this document.

P: Will they do the research on the child?

F: No! You're the only one who's talking to us

P: Hmmm?

F: You're the only one talking to us [the baby is crying on the phone]

F: Oh baby, we will never have a baby! we need you!

P: Ehhhhh oy No problem then!

F: hmmm, no problem, is there another problem you have to get started?

Any questions you have ,you can ask me so that we can start with the questions I want to ask you.

P: Does this mean that whenever they need me in research, will they contact me?

P: eeeh no I hope that once we discuss today, I will be able to ask you all the questions that we need to ask, and we will not ask you other questions later. Instead, you can contact us when you have a concern whenever you want even after this discussion. in that case, I will then give you the number to call.

F:: Hmmmm, So if you agree that I record this and giving me information, it means that, you

\ understand the content of this study,

P: Hmmm

F: Also you had an opportunity to ask questions and you are satisfied with the answers.

F: Also, when you need to take the time to talk to others, in this context, the person you spoke to was your husband as you took time to consult him before participating in this research.

You will also receive a copy of this document, signed and dated .

P: Hmmm

F: Do you agree to participate in this study?

P: yes, I agree

F: Do you agree that this conversation of being recorded?

P: Yes, I agree, no problem

F: Thank you very much

F: Let's start now

P: Hmmmm

F: hmmm yes, you did great as you did agree to talk to me. So as I kept telling you, the purpose of; this study is to find out how your child has been cared for by the doctors and how you took care of him. We want to use this information to help CHUK hospital make a difference in the service they provide but we will not provide your name or profile to anyone!

P: Is there any problem you may have before we start?

P: Nothing, I have no problem

F: The first question

F: Ehh, let's start with the first question. I was about to start, tell me about your time at CHUK. When did you and your baby go to CHUK?

F: Yes, how was it? When did you get to the hospital? Do you remember?

P: Sure, the hospital we entered on the twenty-first of January.

Q: In the year of twenty what?

P: Year of twenty- twenty-one!

F: Can you tell me more about what happened when you were in the hospital ?, Also how did you react when you gave birth to this baby who had GS, they explained it to you! How did you react?

P: As soon as the baby is born,

F: Yes.

Q: As soon as I saw the intestines coming out of the womb, when I gave birth to him at the health center they immediately transferred me to the main hospital, the main hospital also immediately transferred me to CHUK

F: Yes

P: When we get to CHUK, the baby , they fixed his intestine well, they put it in a special bag that looks like a plastic bag. They ended up putting him in a machine. and the intestines started going back slowly in the baby's womb as much as he was breathing.

F: Hmmm

P: They were them injecting him with serums to feed him.

F: hmmmm

P: At that time we were not allowed to breastfeed before having all his intestines back in his womb!

F: Yes

P: So when the baby, the intestines go back into the womb slowly, the intestines go back into the womb and they covered him,

F: Yes

P: They helped us find muscles to inject and increase serum levels

F: Hmmm

P: After returning to the womb, the baby's body swells . all the body.

F: Yes

P: I was scared but because there were others like him, I was patient with that. some time later, he started coming back to normal again until his body was no longer swelling. And he was wearing a probe in his nose through which the milk had to pass.

F: Hmmm yes

Q: So the milk, they gave us needle in which to put the milk and then milk into syringes

F: Yes

P: Then I started from 5 while giving him milk.

F: Yes

P: They were slowly increasing the quantity of the milk until we reached 50

Q: 50 milliliters?

P: No, look at the numbers graded on the syringe

F: hhhhaa! It's measurable!

P: They give you a measure and say maybe you're putting up to five ounces of milk, that could be like yesterday and say you're up to ten today,: until they tell you to stop to a certain level, Now that the milk has reached fifty, they say, look for a baby battle and see if is he able to suck it .

F: Hmmm

*P: I then bought the baby bottle I put the milk in and I respected the graduations of the baby bottle, and I reached the quantity of the milk they ordered me. I was told to reach 50. When they realized he could suck the bottle, they ordered me to breastfeed him.*

*F: alright*

*P: The baby has been breastfed for about two days, he started breathing unhealthily again. So, we stopped breastfeeding and returned to the gavage tube.*

*F: Hmmmmm*

*P: When he breathed a sigh of relief I was back in the gavage tube*

*F: Hmmm*

*P: We slowly fed him through gavage tube*

*until he regained normal breathing .*

*F: Yes*

*P: So when he breathed well, the milk, I resumed to breastfeed, so they also helped him with medicine.*

*I lived in CHUK, but I had come from very far away.*

*F: far?*

*P: I came from far away, and I could not find someone to bring us food from home. So in the morning , people from CHUK, they would give us food. In the morning they fed us with porridge and bread. In the afternoon they gave us a plate of food. In the night , we had to find food on our own.*

*F: Hmmm*

*P: And then at 3 o'clock , that is when they gave us more food. And then at night when we eat what we can on our own.*

*F: hmmm*

*P: you can really see that they were trying their best*

*F: yes yes*

*P: Also, there were drugs that we had looked for , from outside of CHUK. We could find this medications at King Faisal hospital or at Kanombe hospital*

*F: Hmmm*

*P: In that case, they used to provide an ambulance for transportation . Or sometimes, we had to find our own means of transport.*

*F: Ahh yes, yes, so, as soon as you saw a baby with*

*the intestine outside, how did you react, how did you feel? What do you think? How did you feel?*

*P: As soon as I saw the birth of such a child, no one in our area had ever given birth to such a child,*

*P: Honestly speaking, I thought it was something linked to witchcraft. However when we got to the hospital they told me it happens, they told me it happens to others. That is when I started understanding.*

*F: yes yes*

*Q: But I was depressed because even at the health center the doctor had told me that this was also the first time he had seen it.*

*P: From our side( Family's side) we thought we just needed to go home and wait for whatever had to happen to happen. Then the doctor asked for a transfer and they took us to CHUK and now the child is alive.*

*F: So what did the doctors tell you about the disease right away when you got to the hospital?*

*P: Doctors at CHUK?*

*F: No local doctors*

*Q: As soon as local nurses saw the baby, they said that they also saw it for the very first time, and that was the first time, but they immediately transferred us. So they took me to the main hospital. And the main hospital said they couldn't help me as well.*

*F: Yes*

*P: When our main hospital also said that they couldn't, that is when they immediately transferred me to CHUK.*

*F; yes*

*P: So, after you and your child were discharged from the CHUK, what plan did the doctors give you on how you would take care of that child and how did you follow that plan.*

*P: The plan of how to take care of the child after CHUK?*

*F: Yes, the plan of how you cared for the child, what was it like? Did you follow it? Where were you? How long did it take you?*

*P: when I left CHUK, the child ‘s area with bandage seemed to be healed.*

*F: okay*

*P: Then, they gave me medications to be taken from home. They also gave me syringes to help me get the right quantity of the medication. So, I had to look at the numbers on the syringes while pouring the medication for the baby*

*F: yes*

*P: So they ordered me to sterilize those syringes by boiling them because I had just few of them, not a lot.*

*F: Hmmm*

*P: So I used to boil the syringes . I had to give him the medicine they gave me, only once per day.*

*F: hmmm*

*P: I also still have that medicine*

*F: Yes*

*P: So they told me that the baby should be breastfed after you wash your hands. I have to make sure that I have enough cleanliness, and I cover him well.*

*F: hmmm, yes, do you really think that you respected what the doctor told you to do?*

*P: Yes, I acted according to the doctor told me, I was even given a return appointment, and I returned back to the hospital for the appointment*

*F: eeeh, you went back!*

*P: hmmmm*

*F: You did well. So for the help they give you, what do you think about the medical help or the health care that have been given or given to your child!*

*P: So, About the health services they gave to the baby, I appreciated it because they healed him, and I was able to take him with me alive.*

*F: yes yes*

*P: And I'm still breastfeeding him, and I called other parents who had children in the hospital, and they told me that their children are dead. but mine is still breathing.*

*F: Yes*

*P: about vaccines, when I got home I vaccinated him, I vaccinated him. The most recent vaccine he got was that of three months and half. The next time, he will be vaccinated at nine months.*

*F: Yes*

*P: About weight, the baby is growing, any time we go for a checkup, they realize he has no problem with weight.*

*P: Yes, thank God*

*Q: So another question I might ask you, have you ever noticed that your child needs urgent help ? Has any urgent care been even needed to get your child's life taken care of? Explain in depth*

*P: Was there an emergency? you mean what happened at that time ??*

*P: Yes emergency care, have you ever noticed that your child needs emergency care that has led to your child's health being taken care of?*

*F: Did you get to that point ?? [Be patient let buy another airtime , this one, I can hear that it is almost over [calling the participant again],*

*P: yes!*

*F: I would also like to ask you, have you ever felt that your child needs immediate help, have you ever complained that your child is in a very bad condition and felt that he needed more care? Have you ever experienced a scary situation with your child?*

*P: Hmm, at that point, as soon as I gave birth to him, I found it scary,*

*F: Hmmm*

*P: Then they immediately called an ambulance and they rushed me to the rescue. The ambulance then took me to the main local hospital*

*F: Yes*

*P: Even there, they immediately gave us another ambulance to take us to CHUK, you feel that they also immediately gave us an ambulance for a quick rescue*

*F: yes yes*

*F: Hmmm so let’s continue on that question I asked you, can you tell me how it was at home with Jonathan?*

*P:Before he was born?*

*F: eeh as soon as you were discharged, eeh right after coming out of the hospital*

*P: ehh I left the hospital........ actually he was born*

*with [inaudible voice].*

*F: How about weight?*

*P: He weighed three hundred kg and eighty grams at birth , and he weighed two hundred kg and fifty g. when we were discharged.*

*F: yooo, the weights has gone down*

*P: Sure, he had to lose weight through the healing process, he went through hard times.*

*F: hmmmm*

*P: Due to the complexity of his case, the weight had decreased, I came home and within a month, it was even hard for me to hold him in baby clothing because he was too small.*

*F: oooh of course, I can understand!*

*P: Hmmm. So, I was scared as I had been at home for a month and I was afraid that one day I would wake up and he would not be there.*

*F: yes,*

*P: However, he is slowly growing, and now he is starting to laugh.*

*F: hmmm [laughing]. How much weight does he now have?*

*P: When I vaccinated him recently at 3.6 months, he was 6kg.*

*F: eeehh? Thank God. Can you continue to share with us things that may have been difficult for you? The problems with your finances or others as ....because you see he is still young. stress or means and more that might have been difficult for you.*

*P: I was young , he is my third child! So in terms of payments at CHUK I ended up without any other money left. I was unlucky enough that I was accidently also categorized in the third category of UBUDEHE, which I do not deserve.*

*F: Sorry......[ there were some noises in the backgrounds and I lost her].: hmmmmmm [audible noises] be patient I don't know what happened,*

*P: Instead I thought you were listening to me*

*F: Yes, now I can hear you better.*

*Q: I mean, in terms of means, I was in CHUK for two months*

*F: hmmm*

*P: when I was categorized in the third grade with UBUDEHE , I didn’t deserve this.*

*F: HMmm*

*P: When I was in CHUK I paid a lot of money as somebody who is classified in the third category*

*F: Yes*

*P: You know whenever they took him for exams, I had to pay, mediation and hospital discharge, I had to pay many thousands of money*

*F: It's thousands*

*P: Now that I was there in the corona outbreak, there was a stay at home and now the car to take home was not easy to find . I tried to wait for the ambulance too, but it was not available. and then I rent a car to take me home*

*F: hmmmmm*

*P: It's 50,000 Rwandan Francs that got me the car.*

*F: Yes*

*P: My husband is working alone and I am at home just sitting there, you know this kid I wouldn't take him to work because I cannot take him on my back . Whenever I try this he shows signs of discomfort. I cannot take him to work with me on my back. I am just home eating, doing nothing.*

*F: Yes of course*

*P: So now you understand that wealth at home was really disturbed. Things have gotten worse here*

*F: Yes, and maybe mentally, mentally, did you feel happy? depressed? How did you feel?*

*P: In my mind when I was in CHUK I felt like a loser*

*F: Yes*

*P: I used to think that this kid was going to die and I was not going to find a way to take him home*

*F: Yes*

*Q: I was discharged with so much of stress in my head I guess the stress is there still*

*F: I understand. It was a hard time*

*P: When I got home I found that we had a lot of debts because every time that the husband sent me money, that was the money which he borrowed .*

*F: Yes*

*P: So you see, even today, it is good heartened people visit me sometimes and provide something to help me.,*

*F: Hmmm*

*P: That is how I am able to find myself food to keep me healthy for the baby.*

*F: I really understand.*

*P: but you see that kid I cannot even put him on my back because he directly breaths bad. Because the way you know he's in the [inaudible voice], when he breathes I feel like you're in pain ( when she puts him on her back)*

*F: ooh yes, I see*

*P:That is why I do not go to work with my husband, I sit at home and I don't work.*

*F: yeah, I understand, so do you see anything that has changed in social life? , your relationship with your spouse, your relationship with your neighbors, your relationship with your friends, do you see anything that has changed since you gave birth to a child with this problem, do you see anything that has changed?*

*P: In my relationship with my husband, the man welcomed it because we all.... this situation is for us both! We are both his parents. The child was born from us,*

*F: Hmmm*

*P: He also tried his best to help me*

*F: Hmmmm*

*P: And neighbors, who come to visited me at home and my husband has not changed regarding our relationship*

*F: Hmmm thank God so much, thank you for sharing your life with me, so now baby, tell me what the baby is like? What is life like? do you compare him to other kids of his age?, do you see his peers, how does he do now?*

*P: Normally, I left CHUK when he had started to breastfeed*

*F: Hmmmm*

*P: About the nutrition, he is just breastfeeding because I didn't start giving any other food. He is breastfeeding. He is still breastfeeding.*

*F: Hmmmm, thank you very much*

*P: So growing up you see, it is likely he can't grow up well because you see he suffered a lot from the illness now that those who weren't sick are growing up well since they do not even have issues related to means compared to us with this case.*

*F: Right*

*P: And you see I have paid like three hundred thousands Rwandan Francs (300,000 ) which I got after selling my possession. SO, understand that my kid cannot compare to kids born in normal situations.*

*F: hmmmmm.*

*F: hmm, yeah, I see!*

*F: Hmmm so can you tell me something you wished you had known about your child ahead of time?*

*P: Care was needed, at birth, because if it weren't for it, my baby wouldn't exist,*

*F: Well, there are times when you want to, like something you wish you had known before, before giving birth, when he was still in the womb, do you feel there was some information you should have known about a child like yours? Was there any information you should have known before you knew he had the GS problem?*

*P: How would I like to know the information, while I did not know if he would be born like that.*

*F: Hmmmm, did you know that a doctor can diagnose a child with a stillborn problem?*

*P: At the doctor's place, I feel that a pregnant mother should go through the machine and see the baby's health.*

*F: Does that mean the next time you are pregnant you may be able to go through the machine to check if there is a problem with the baby and follow up?*

*P: Unless you go to the big hospital, the big ones are the ones who have the equipment, so when it comes to that , isn't it financial?, it is expensive!*

*F: Yes it is a resource, but if you have insurance it is possible, you can try*

*P: Mutual de santé insurance is available, so I would like to try the next time I get pregnant.*

*F: hmmmm*

*P: would they have seen that if I had gone there?*

*F: Yes, there are certain months, there are certain months when doctors can look at the baby and see that a baby has a problem ..*

*P: So knowing that would also give me a hard time, and it would be hard for me to deliver.*

*F: Is that so?*

*P: I would be discouraged if I knew it before, and was probably not going to be able to give birth. I would be scared and become weak.*

*F: Yes, that's also possible. Thank God all is well now.*

*Q: Hmm*

*F: so,*

*P: HMmm*

*P: So what would you say to a parent if they had a child with a problem similar to yours?*

*Q: If a mother has a child with a problem like mine, what can I say to her? just to go to the hospital. In case she gave birth from home, she just would rush to the hospital to seek help. In case the initial hospital cannot help, they would transfer her to somewhere else.*

*F: Hmmmmm, what about taking care of the baby and more?*

*P: As for taking care of him, I would tell her to breastfeed and be clean, which is to breastfeed and keep her clean.*

*F: yes, thank you very much so I don't know if there is anything else you would like to add, is there a problem or whatever you feel you can say*

*P: I have a problem, you see I have limited resources, you have to help me if there is a way.*

*F: yes thank you for your request we have heard and we are bringing it to the attention of the authorities. Thank you for the time you gave me and keep taking care of the child. God bless you thank you very much*

*P: yeah, thank you too*

*F: Yes*

*P: The medicine they gave me to give him I am still giving it to him without knowing how long It will take to stop the medication.*

*F: Ehh, you mean they gave this drug, and they did not tell you how long to use it or when to leave it.*

*P: Hmmm, they didn't explain it to me when I should stop it and I didn't ask*

*F: Yes, I will ask for you tomorrow, tomorrow I will ask for you, and then I will come back to you.*

*P: Hmmm, they gave me a medicine called [I don't really understand the name of the medicine he was given]*

*F: Yes, Dr. Edmond, I would like to ask him this question .*

*P: Ehh if I can kept giving it to him*

*Q: What is the drug called?*

*P: Buresiforo, How to read this! [Laughing in the phone]*

*F: What is the name?*

*P: We are not educated!*

*Q: What's the name? Repeat for me once and for all, do not worry , please!*

*P: buresefore*

*F: Buresefore?*

*P: Hmmm,*

*F: Yes*

*P: it is written there" vitamin C"*

*F: Ahh, okay I'll ask for you*

*P: Hmmm*

*F: Yes, thank you very much and say hello to Jonathan*

*P: Yeah, you too!*

Recorder stopped: :07
