# Supplementary material for: Exploring the lived experiences of parents caring for infants with gastroschisis in Rwanda: The untold story
Source: PLOS Glob Public Health. 2022 Jun 15;2(6):e0000439. doi: 10.1371/journal.pgph.0000439 (PMC10021215; doi:10.1371/journal.pgph.0000439)
Supplement: S1 Data — (ZIP) [file pgph.0000439.s002.zip › S1_Data/S5_Text.docx]

**BB 9 English Transcript**

F1: Hello, you have been invited to participate in this research project exploring the lived experiences of parents and educators of infants who received hospital-based gastroschisis care) because you meet the requirements for inclusion into the study and can offer unique information regarding the questions we are going to pose in case you accept to take part of this study. Before accepting to join this research, it is essential for you to understand and take into consideration the contents of this form, since it contains important information to assist you in deciding whether to participate or not.

This project is being conducted as a core requirement for the Master of Science in Global Health. Delivery at the University of Global Health Equity. The project has received required ethical approval from UGHE and complies with international ethical standards for research to be carried out in Rwanda. Additional permissions have also been obtained from the CHUK and the IRB of the University of Global Health Equity.

About this consent form

Before agreeing to participate in this research, you have to understand and take into consideration the contents of this form, since it contains important information to assist you in deciding whether to participate or not to participate in this research. Please take the time to review the document, seek advice from your family and friends in case it’s necessary. If you have questions about this research or this form, do not hesitate to ask. If you agree to participate, you will sign this form and be given a copy for your records. Since we are using the phone, you will not sign it immediately, but we will send a copy of it and sign it, but now you will let me know if I can record this discussion.

*Participation is voluntary, you can either participate or refuse, but it is your will to decide and it is important because it will help us. It will help CHUK to provide better services than the service they offered to you.*

*P: Maybe you see that this research is all about improving health services, you see what happened to me, can it happen to anyone else?*

*F: Yes*

*P: Right?*

*F: Yes*

*P: It is important to carry out in-depth research to find answers to these questions. When you record, it means that you will help me and I will help you since we want to build a healthy life in us.*

*F: Yes, it is true.*

*P: So I think there is no problem you can record, so that in case it happens to someone else when you give the record, because you understand, this is sensitization on how the treatment went and the benefits of treating the children who have the GS problem.  Parents with such children would feel hopeful and after basing on the information they hear from us, and I have hope that their children will be treated and healed.*

*F: Yes, thank you very much*

*P: you can record, there is no problem.*

*F: Thank you very much, maybe you may be wondering, “What are my benefits?” Of these, I am so impressed that you immediately got this. Anyway, it just required me to explain so that I could shed light, perhaps, there are no immediate benefits we would promise you but the information you will provide, as you said, will help in the development of services to be delivered in the Department of Surgery for newborn babies at Kigali University Hospital (CHUK). Also this information you are about to provide, it will help other parents and caregivers whose children face this disease. So you see, you realized it right away. So the other thing I would add is that the information you give will be used but your names will be disclosed. Isn't that right? We will not reveal your name or your identity.*

*P: Even if my names and identity would be revealed, there is no problem*

*F: Yes, Thank you so much;*

*P: Because*

*F: Yes*

*P: If there are any good things I found out, it's not good for me to keep it a secret. If I can tell you that I gave birth to a child with GS and he healed, it is not shameful, children with disabilities are not to be excluded. They hear from the person who faced the same issue and say, "Is that true what the person is saying?" So whether my name is mentioned or not, it doesn't matter to me. Because the most important thing is the information that builds the life we want.*

*F: Taking part in this project is entirely voluntary and you have the right to change your mind and leave the study at any time. We may reach a certain point and you feel like stopping the discussion, we can really do it. Choosing to not participate in the study or withdrawing at any point will not involve any penalty or loss of benefits to which you are otherwise entitled.*

*F: You can also say, “If I have a problem and I want to ask, who will I ask? From where will I ask?” I will give you the phone number or the email address of the research team lead. Another thing I would say, maybe I would ask you. That is to say, if you allow me to record the discussion for this project, it is because you have understood all the content of this article, and you have had the opportunity to ask questions and have been satisfied with the answers?*

*P: Yes*

*F: If necessary, take a moment to think about it or talk to other people to help you make a decision if you feel that you can’t decide for yourself, or if you agree that you will be given a copy of this signed document with this date, you have agreed to participate in this study.*

*P: There is no problem indeed.*

*F; Yes, Do you allow me to record this discussion?*

*P: Yes*

*F: Thank you very much, thank you very much for agreeing to talk to me. The purpose of this interview is to find out how your child was taken care of at the hospital and how you have taken care of him. We would like to use this information to help CHUK Hospital to make changes in service delivery but we will not disclose your name or profile to anyone even if you have given us permission.*

*F: Do you have any question before we start?*

*P: I have no question.*

*F: Thank you very much*

*F: For the first question, I would like to start with the moments that you had at CHUK*

*Q: What?*

*Q: The moments that you had at CHUK. You were admitted on which date, which year? What was your child's name or what was his name? Can you tell me in details what happened when you were in the hospital? Hmmm*

*Q: Yes*

*P: Actually, I am called XX,*

F: What?

*P: My name is XX, I live in Gatsibo district,*

*F: Yes*

*P: The sector is called Gitoki*

*F: Yes*

*The cell is called Nyamirama*

*F: Yes*

*P: I gave birth to a baby named XX*

*F: XX*

*P: I gave birth to him at Gatsibo District Hospital Named Kiziguro.  I gave birth to him when he’d been in the womb for seven months.*

*F: EEHH Yes*

*P: He was born with a problem of intestines growing outside*

*F: Yes, oh God!*

*P: After giving birth, they told me that there was nothing else they would do other than rushing us to the referral hospital, where I got the bed was there at CHUK. Because we were in the COVID period, admitting many people was prohibited. They hospitalized patients according to the beds available.*

*F: Ooh! Yes*

*P: They transferred me to CHUK. I gave birth to him on the fourteenth of February.*

*P: I gave birth to him around ten o'clock.  At eleven , that is when we got up and came to Kigali so that CHUK could help us.*

*F: Yes, in which year please?*

*P: This year, I gave birth to him in 2021.*

*When we got there, they immediately started helping the baby. The good thing I loved, they started helping the baby while I was still looking for the baby's documents.*

*F: They immediately gave me emergency medical assistance*

*F: Yes*

*P: They told me to go find this and that, and while looking for documents, they immediately started operating my child. After helping him, after fixing the intestines, they immediately took me to the general ward where the children with the same problem were living and being taken care of.*

*F: Hmmm*

*P: I think that would enough, if there is anything else I would add, you can tell me.*

*Q: Yes, one more thing I would like to add, I would like you to tell me how you reacted after giving birth, giving birth to a baby who’s been in the womb for seven months, moreover, he was born with this GS disease, when you saw his external intestines, how did you receive the news? How did you feel? How did you react?*

*P: So for me, given the fact that I gave birth to him at seven months, I didn’t expect it. But I felt that it was a problem giving birth to a seven-month fetus, especially that I heard that children born at seven months live.  But you understand that it was a challenge for me to give birth to a premature baby.*

*P: It turned worse when the doctor told me that he had an intestinal disability which was something else. I felt dizzy and said,” will a child born with this disability and born prematurely be able to survive?” But the doctor told me not to worry. They told me that doctors are treating him and even though the baby is born with this problem, he will recover. He kept telling me how they do it, and I felt comforted. But I said I'll consider it when I see it.*

*P: Yeah,*

*F: Yes. The doctor did a great job.*

*P: Yeah*

*F: When the doctor saw this baby, you were telling me a little about it. After seeing that the baby was born with this problem, he said that it was which disease? What did he tell you about the disease right away?*

*P: He told me that I had given birth to a baby boy but that he had a disability of external intestines. But he said that doctors will help the baby to recover. Yes, I don't think he gave further explanations.*

*F; Yes, there is something else I can add. After you and your child were discharged from the hospital, which plan they give you for taking care of your child, and how did you follow that plan? Where did you go after getting discharged? For how long did you stay before getting discharged? Yes*

*P: Maybe before talking about how I got home, let me just go back to the days I spent there at CHUK because I had stopped on the part where they were done operating the baby.*

*F: Yes*

*P: During the days when I was at CHUK, he was put in an incubator where there were other children who had the problem of GS and in a critical condition. What they encouraged us to do was to maintain cleanliness, we went in after washing our hands and followed the cleanliness guidelines to prevent children from getting infections that we had brought from outside that may worsen their condition.*

*F; Yes*

*P: Another thing I liked, the doctors came in about twice in a day. The senior doctors, who came to see the children, came in at eight o'clock in the morning, to see how the children were doing, to check and come to see what had changed and what had happened. And in the afternoon around 2 p.m. they came back and looked at what the kids' day went.*

*They checked on their health and followed up with them every time. This means that doctors were the ones who came, but we could spend the whole day with the nurses.*

*F; Yes, yes*

*P: Another thing I would suggest, the place where the children were hospitalized is small. Because you could find that the room where we stayed had 8 incubators, right?*

*F: Yes*

*P: You see the room was so small. And another thing, we could spend the day with two to three nurses. You find that if the children are eight and the nurses are three, they have to take care of all the children, and you find that the lives of those children are very fragile since people  need to keep an eye on them, and you find one who is looking for a vein, the nurse is looking for the vein from where they are going to make injections , and you find that for another one, the probe got out of his nose, and  there might be another one that was on oxygen support and you find that the device got disconnected or that the water had run out and you find that reaching all those children at the same time is impossible.*

*F: Yes*

*P: In the meantime, based on what I saw, they tried to take care of me since we were eight people, the doctors were working with commitment but you found that they had responsibilities beyond their capacity. So you find that it would be nice to add more nurses who can take care of the babies in the incubator.*

*F: Yes, tell me what happened after getting discharged. You were discharged from the hospital*

*P: What I am requesting you to do is that you add more space for taking care of these children, and add more nurses to care for these children, because you understand that if there are three nurses looking for a vein of a child, they will call for another child and say that s/he having shortness of breath, you understand that these are movements of coming and going. You find them getting tired and it becomes challenging for them. They should increase the number of nurses on that post to find ways of taking care of children in the incubators.*

*P: So after being discharged from the hospital, they said goodbye to me and gave me instructions. They gave us medicine to go and give to our baby boy.*

*F: Yes*

*P: They told me that the medicine kills infections*

*F: Yes*

*P: I think it was griconozole that they gave me.*

*F: Yes*

*P: [she responds to someone calling her in the background. (It seems they got a visitor.)]*

*P: [Be a little patient, I am talking to some people for a while but you are welcome (She tells the visitors)]*

*P: Hello*

*F; Hello, I'm there,*

*P: That is to say, my baby was in the hospital and I have been there for three weeks.*

*P: The doctors told me that my child had recovered, the navel was the only thing left to heal because they were showing it to me and so they told me that when it came to it, just like the other babies who were born normally, his navel is similar to the one of normal babies, and he will therefore recover. They told me to cover him, and protect his navel from water*

*F: Absolutely yes*

*P: The baby’s warmth and navel’s recovery didn’t delay, and the child recovered fully.*

*I went back home on the 17th of March 2021, and returned for checkup on 8th of April in Kigali, and they told me that the baby had fully recovered.*

*F: Praise God. (child crying in the background). So what do you think about the service or treatment given to your child? What did they do for you and your child? What did you like? What did you give? Tell me how they took care of him, the service they gave to him, tell me something about that.*

*P: The first thing I liked was how they taught us hygiene so that children could be free from infections. Because you see, if you take care of the child in dirtiness, he won’t heal.*

*F; Yes, you are right*

*P: They tried to take care of us, they treated the children well, and we came home fully recovered.*

*F: Yes. Is there any time you sought emergency healthcare?*

*P:  They really did their best*

*Q: So, was there an urgent situation that made you seek immediate medical assistance for your child? Was there a time that reached and you realized that your child needed immediate medical assistance?*

*P: I was lucky, they followed up with the child frequently, I never had an urgent issue but whoever faced it, the nurses who were there had the capability of calling doctors who checked on the child and helped him/her. Whether it’s someone in need of oxygen support, or someone who had an urgent issue and they helped him/ her, in brief, they could consolidate their power and keep him alive.*

*F: Yes, thank you very much ... Let me continue with this question I asked you.*

*P; Yes*

*F: Can you tell me what it was like to be at home with the baby… I didn't even ask the child's name. Can you please remind me?*

*P: He's called Ephron Impano*

*F: Impano Ndungutse Ephron, how do you call him at home?*

*P: He is called Ephron*

*F: Ooh, you call Ephron at home.*

*P: Yes*

*F; So, tell me what it was like, being at home with Ephron after getting discharged.*

*P: At home I had to cover him a lot*

*F: Yes*

*P: And before I go to him, I had to wash my hands and keep the hygiene to protect him from infections. I tried to eat and drink because I was allowed to breastfeed him. So, I maintained cleanliness at home and when he defecated, I cleaned him whenever he dirtied himself. I also made myself clean because I had to breastfeed so many times but after having bathed.*

*F: Yes*

*P: Another thing is that I covered him and protected water from entering his navel.*

*F; Hmm, yes, thank you very much. Share with us the challenges that you faced. For example the funds, the job that you did, how was it going? The means to take care of a child like that, do you think your mental health was stable? Physically, what were the challenges that were caused by giving birth to a child with this condition?*

*P: In fact, being born like that, I think there was no condition he lived through that caused him to be born that way because first of all, I can’t say that I was missing food, or that it was my spouse who had insulted me or a misunderstanding, no!*

*F: Yes*

*P: I think that what was just meant to happen since I wasn’t the only one with that problem. There are those who say that maybe they poisoned me... no! ... Even though people poison, they can’t alter God’s plan.*

*F: Maybe here, what I asked you, I asked you something that was hard for you because you see that now you are a teacher. What happened at your work? How did it go with the funds? Did it have an impact on you? That’s what I wanted to ask.*

*P: In fact, my work gave me a maternity leave as a mother. At home, I would say that people are meant to be supported by others. No one is self-sufficient. I left CHUK after paying a lot of money.*

*F: Hmmm, a lot.*

*P: Three weeks were a lot, the economy gets shaken, plus finding the meals. You also know about it as someone that lived in Kigali. In case someone knew it and had the heart to help me, since my salary was not sufficient. So, I got friends and relatives, and one who had one thousand Rwandan Francs could help me and life would go on. For CHUK's debt, I was lucky enough to leave the place after clearing it. For the lifestyle, you have to become patient. Even if I quit eating but return home with  a healthy baby, that’s what matters to me.*

*Q: You understand that here at home the economy got down, but we have had friends and one who visited me could bring a kilogram of sugar and life would go on. But you understand that I was helped by others who were close to me and supported me.*

*F: God bless those people.*

*So, for your mental health, it never got backslid? You never had mental depression? How was it like?*

*P: The depression is inevitable. You see, I kept asking myself, I gave birth to my first child, I gave birth to him prematurely, he is born with so many problems ... I wondered a lot and could ask myself, “what will his life be like and how will it go? But maybe my luck is that I have a husband who may have some level of understanding and he could say that if God gave him to us, the baby will live. Just like he may be born a few months earlier or be born at 9 months and face the condition in his growth. He strengthens me and I keep getting stronger, and what I encounter, I get more hope that my child will get better and live.*

*F: Yes, hmm, so did giving birth to a child with a GS problem affect your relationship with your husband? For your neighbors, or your workmates? Did anything changed?*

*P: In the society, that is inevitable, and people who rejoice over one’s sorrow are also there*

*F: Hmmm, yes*

*P: There are those who say that I gave birth to a baby with a problem of intestines and add other things, but I get stronger and say, “Living in this world requires vigilance, everyone has his/her own life trajectory.*

*F: Yes*

*P: I calmed down and got stronger since we were both working on our home. I calmed down and saw that my husband had no problem with it. I calmed down since I had no control over external factors.*

*Q: Thank God.*

*P: It is not that easy*

*Q: So, thank you so much for sharing your life experiences, how is the baby's health now??*

*P: The baby is turning four months tomorrow,*

*F: He is a man!!*

*P: But now I have a question to ask, since you follow up with their lives and I am asking for help. Since then he has never been sick but now he is having pimples on his face and now it is aggravating to an extent that the hair is getting off. I went to the hospital on Sunday and showed it to the doctors and they prescribed crenazole. When I applied it, I used it on Sunday and Monday morning, and when it reached the evening, it had become a lot, and they were bursting while discharging water.*

*I wondered the root cause of that and felt confused, so in this study, if you are with dermatologists, maybe you can share this question with them and if they have medicine, they may let me know and I would go to find it and buy it, in hopes that he recovers and his life continues.*

*F: Yes: yes I will definitely ask for you, I will ask that question for you and I will call you. For the issues concerning his diet, breastfeeding, because you see he has four months and has not yet reached the time to eat, but does she breastfeed well? Do you see him growing up like others? How is it going?*

*P: The baby is breastfeeding well because I left the hospital when he was already breastfeeding. And for the matters concerning his growth, when I lastly vaccinated him, we found that he has five Kg.*

*I am returning there in May, then we will check his weight, at that time he was five kg and fifty-two inches tall. He can see and hear, right? I see he has it all.;*

*F: Does he laugh?*

*P: Sure, he laughs, he talks*

*P: Did they tell you that his weight was normal?*

*P: Yes*

*F: Thank God*

*P: So can you tell me something you wish to have known about your child's life or the care that was needed? Maybe something you wish to have known when you were pregnant or when you gave birth to him ... is there any information you wish to have known about your child before knowing that he had that problem?*

*P: One thing I asked myself after giving birth was this question: if you go for a pregnancy test like I did three months before delivery at the main hospital ... passed through the echography, and then went there and they told me that the baby had no problem. Even when the pregnancy was seven months, I went there to see how the baby was doing because I was having pain from the womb and they told me to go and see how the baby was doing and they told me the baby was alive and had no problems. I asked about the sex of the child, but they told me to be contended with any sex since he/she will be a gift from God. But they never told me that the baby had a particular problem.*

*F: I'm not a doctor but there is a time you can go to the hospital and they can see if the child has a problem. I will ask for you and they will give you an answer since I am also a parent.*

*P: Maybe another question, someone asked me if I hadn't done a lot of work in my pregnancy.*

*F: No, it's not a matter of the work you did and we pose these questions to experts.*

*F: Another question, what would you say to a parent if they had a child with a problem similar to the one of yours?*

*P: First of all, they should give birth at the hospital, because when you give birth to a child with a problem, the doctors check him, and when they find the problem complicated, they transfer him to a competent hospital.*

*F: Clementi, thank you for having this discussion with me, do you have any other questions?*

*P: Yes, the suggestion I have is that you would talk to us more often and find out how the kids are doing, and ask us how they are growing as you follow up with them.*

*F; Thank you for the suggestion! it is a really nice point! I will ask this for you*

*Alright, greet Ephron on my behalf*

*Greet him for me and may have a great time, thank you*

*P: Yes, that's right*

*F: Bye*

*P: Bye, does your number use whatsapp?*

*F: Yes, yes, I use whatsapp, contact me anytime*

*F: Thank you, bye!*

Recorder stopped: 00:35:24
