# Supplementary material for: Exploring the lived experiences of parents caring for infants with gastroschisis in Rwanda: The untold story
Source: PLOS Glob Public Health. 2022 Jun 15;2(6):e0000439. doi: 10.1371/journal.pgph.0000439 (PMC10021215; doi:10.1371/journal.pgph.0000439)
Supplement: S1 Data — (ZIP) [file pgph.0000439.s002.zip › S1_Data/S6_Text.docx]

**BB 8 ENGLISH TRANSCRIPT.**

**MODE: Hello**

W1: Hello

**MODE: Good morning**

W1: Yes

**MODE: You are doing fine?**

W1: Yes, no problem

**MODE: Be audible enough, I am not hearing you**

W1: Alright, talk to me.

**MODE: Yes. Thank you for accepting to have this conversation with me.**

W1: Yes

**MODE: As I told you, before we start this conversation, the purpose is to find out how you child was taken care of at the hospital and how you took care of your child**

W1: Yeah

**MODE: Do you have any question before we start?**

W1: No question

**MODE: Okay, when was your child admitted to the hospital?**

W1: On 28^th^ March

**MODE: Which year?**

W1: This year, 2021

**MODE: This year?**

W1: Yes

**MODE: So, what was the span between the time of birth and the time of taking him to CHUK?**

W1: After giving birth on that very day, I gave birth before noon and reached at CHUK at around 10 p.m.

**MODE: Hmm**

W1: It was on that date that I had given birth.

**MODE: Okay, for how long were you in CHUK?**

W1: For a month, I arrived on 28^th^ March and left for home on 27^th^ April.

**MODE: So, how old is your child?**

W1: 2 months and a half, don’t you think that it is the right calculation?

**MODE: Yes, the baby is a boy or a girl?**

W1: A boy

**MODE: Okay, I would like us to start from the moments that you spent at CHUK, right? On 28^th^ March 2021**

W1: Yes

**MODE: You and your child had gone to CHUK, right?**

W1: Yes

**MODE: Can you tell me in details what happened when you were at the hospital?**

W1: We reached there, and I found the doctors there, and they immediately received the baby. They started treating him, and they kept doing the follow up. It was a child in a critical condition, tiny and born with little weight.

**MODE: Like how many kilograms?**

W1: He was born with 1 kg and 990 grams.

**MODE: Hmm**

W1: Yes, they kept treating him, and did whatever they could, he also had issues of breathing.

**MODE: Speak louder, I am not hearing you. You said that he had which problem?**

W1: A problem of not breathing well, during that time he was put on oxygen support for 3 weeks, and they later put him in the device that tests the heart, they found that the heart had no problem, but there was a tiny hole that other babies also usually have. They did blood transfusion to increase the quantity of blood. We later saw that the problem of not breathing well was solved. Time came and we were discharged from the hospital, they could check him and tell me that his blood is fine and has no infection, so the time came, and we went back home. When we were discharged, they had fixed the intestines, but they had covered the area when it had not yet fully healed. So, I reached at home and kept taking him for covering that area at our local hospital. It reached a time and the wound closed, and the nearby hospital also discharged us, and now the wound is whole. Now, there is no problem, and the area can lie with no problem.

**MODE: Okay, that is nice.**

W1: Yes, he usually has a cough, but he is getting better. He has a problem of catching a cough, and it leaves him in a critical condition.

**MODE: Ooh**

W1: But he is getting better slowly by slowly, now he has 3kgs, so there is no problem.

**MODE: Hmm**

W1: Yes

**MODE: On that day when you gave birth to that child, how did you react when you saw that you had given birth to a child with external intestines?**

W1: We were startled, and the doctors were startled as well because it was their first time seeing a child born like that. So, I felt that my struggle was in vain because I never knew that the condition would be treated so that a child may live.

**MODE: Hmm**

W1: I gave birth to him at Nemba Health Center, and it is near the hospital, so they immediately took me to the hospital like 2 or 3 minutes after giving birth, when I reached there, I found there doctors and we conversed, and they told me that they are about to transfer me to CHUK.

**MODE: Hmm**

W1: I asked them, “why are you stressing me?” I told them, “Why are stressing me? Can they treat this child and he survives?”

**MODE: Hmm**

W1: They told me that at CHUK there are skilled doctors and added, “find the money soon and go, the more you delay, the more you decrease the chances of your child’s survival.”

**MODE: Hmm**

W1: At that time, we were not ready and we didn't enough money. We had to search for the money till it was late in the evening. However, the time reached and we went. But I went with no hope, feeling that it was a waste of time. When I got there, I found other kids who were like mine. I found those whose intestines had been fixed backed in their bellies, their parents comforted me, and told me that their children were also in a similar condition. So, when I was in the hospital, I saw that they were fixing intestines slowly by slowly and returning them in the holes that they had left. The time came and they returned in the belly, and they covered up the place. After returning them in the belly, I started fetching breastmilk for him, on the scale I was told. A time came and they told me to start breastfeeding him, when I breastfed him, he started growing. And he got better slowly by slowly. So, when the time came, they discharged me, and I went home. I got an appointment to return after a month. I returned him on 24th May (the appointment day), the doctor examined him. He asked me if there was a problem, and I told him that he had a cough. So, he prescribed another medicine, and I left. That is how it went; I am doing well with my child.

**MODE: Hmm, that is awesome.** **So, what did the doctors tell you about the disease? After giving birth to him and realizing the situation, what did the doctors tell you?**

W1: The doctors who helped me to deliver?

**MODE: Hmm**

W1: The doctors who helped me to deliver? Hello?

**MODE: I can hear you**

W1: Well, those who helped to deliver were also astonished because it was their first time giving birth to a child like that. I sought explanations and asked them, "do you think that this condition can heal?" Those who had helped me deliver at the health center immediately told me that they do not know those things. They said, "it is our first time seeing this, do not ask us, only God knows."

**MODE: Hmm**

W1: The doctor I found at the hospital is the one who comforted me.

**MODE: Which hospital?**

W1: The central hospital found at Nemba, I had given birth at Nemba Health Center

**MODE: Yes**

W1: Yes, so I found him there and he comforted me as he told me that it is curable, and they do not have the means to treat it but said that at CHUK they treat the condition and it heals. He said to me, "Have faith." He saw that we were delaying, I was saying that I had no money, and wondering if the condition could be treated. I felt like I did not care. He saw that we were delaying and told us, “Did you know that you are bad parents who do not want to treat the child. He added saying, "this child can live and be a blessing to you, even though you are refusing to take him for treatment." Deep down we were telling ourselves, “It is just that doctors know how to comfort people, this cannot recover." So, we left the place with no hope. I regained composure when I got there and found parents that comforted me, telling me that their children were also like that. I found those who had recovered, and in a few days, they returned to their homes, then I began to have hope.

**MODE: Hmm**

W1: I was also surprised to see that they fixed his intestines. They fixed them in a period of one week and they pushed them and I also saw that they had put them back. At first, they were various complicated things, after fixing them, I saw that he had turned into a baby. When we got to the point where I started breastfeeding him, I had hope that the child would survive. That is it, now he is doing well.

**MODE: Okay, that is nice.**

W1: Hmm

**MODE: After getting discharged from the hospital with your child, which plan did they give you for taking care of your child? And how did you follow that plan?**

W1: They gave me a plan of maintaining his cleanliness, and they gave me a plan of taking care of myself so that I may get the breastmilk and feed him.

**MODE: Hmm**

W1: Those are the instructions they gave me, and told me that when I find him sick, I should immediately take him for treatment.

**MODE: Hmm**

W1: That is the plan I received when I was discharged from the hospital.

**MODE: So, they told you to take him to which place (hospital) when he gets an emergency case?**

W1: At the nearest Health center

**MODE: Okay**

W1: Yeah

**MODE: What do you think about the medical assistance or the treatment that your child received?**

W1: My thought is that doctors work with God to recreate since I used to think that it was an incurable disease. Now I accept that nothing is impossible, I was amazed by the skills of those doctors, I can even testify that CHUK really has good doctors.

**MODE: Hmm**

W1: I think that maybe God takes their hands, maybe He is the one who does the whole thing, because I was seeing how they were pushing the child’s intestines.

**MODE: Hmm**

W1: I was still confused wondering if they will return to their rightful place like the original intestines created by God. I still ask myself that question to this day. I ask myself, “won’t he have a complication in his belly?” They keep pushing intestines while other intestines were perfectly fixed in the belly by God?

**MODE: Hmm**

W1: I ask myself that question, I wonder if it will not have an impact on him.

**MODE: Do not worry, they fixed them, and he did not die, didn’t you tell me that he is doing well right now?**

W1: He is doing well, it is just that he was born tiny, and he is still tiny. But you look at him and find that he is a sharp baby, playful like others, and now he can look at someone and smile, there is no problem.

**MODE: So, do not worry or feel depressed, right?**

W1: Yes

**MODE: They will grow since they cannot put them in a spot which is inappropriate. Since they fixed them and he managed to survive, it means that he has a purpose, and God will continue to protect him.**

W1: Yes, He will be good unto him, even when he suffers from another disease, I say that he cannot suffer from something tougher than the condition he was born with. I have hope that no disease can beat him.

**MODE: Hmm, since what he recovered from was the most complicated**

W1: Yes, it was complicated. He had many intestines. God, I could wonder, “will these things recover in his belly?” You could see that he had a bent belly, and the external intestines were huge, oh God, even the doctors were astonished and said, “how comes this child has huge intestines?” But they fixed them, oh God, and they returned inside the belly, and they covered the belly. So, we were discharged after covering the place three times.

**MODE: Hmm**

W1: At Nemba, they kept covering him, and as they removed the cover, I could see that the place is merging, and the hole was closing. If the area had become whole, at Nemba they would have told me that they will no longer cover him. So, I keep doing that, and I take him back home.

**MODE: But let me hope that you returned to the health center where there are doctors who thought that recovery was impossible so that they can see how he recovered.**

W1: Hhh, do you know what happened when I brought him to get covered as I came from CHUK, and all the workers of the health center gathered to see him, as they took pictures since they had photographed him at birth?

**MODE: Hmm**

W1: They were amazed, oh God, they were amazed and joyful since they had transferred me elsewhere because they had no hope. But they were happy when they saw me bringing him back alive, and everyone was exclaiming, “here is that child.”

**MODE: Hmm**

W1: They were also amazed. Even now when every doctor meets me, they raise the cloth to check and see how the baby is doing.

**MODE: Hmm**

W1: Yes

**MODE: These are extraordinary things, God is omnipotent**

W1: These are miracles of God, and every doctor was picking a name for him, one was calling him Igitangaza (Miracle), and others Mvuyekure (I come from far) … so they picked names for him.

**MODE: They were extremely happy because he had recovered.**

W1: Yes, extremely happy

**MODE: It was joyful indeed seeing someone you thought to die soon alive.**

W1: Yes, by then when I was at CHUK, the 2 doctors who had helped in delivery could see people from my neighborhood and ask if the child is still alive. And they could respond that they talk to me and hear that the child is still alive.

**MODE: Hmm**

W1: And the neighbors could ask one another if the child is still alive.

**MODE: Hmm**

W1: When they saw that he recovered, they felt amazed, the neighbors came to see how the child was doing, many people were amazed indeed.

**MODE: Hmm**

W1: Yes

**MODE: Was there need of immediate assistance that made you want to see your child getting treatment? Or did your child face a serious problem that made you rush to the hospital for treatment/checkup?**

W1: After getting discharged?

**MODE: Yes, when you had reached at home**

W1: I took him home, and I used to take him for covering. Another illness that made me to take him to the hospital was when he got a cough. I gave him medication and he did not recover, when I went to exchange the medication, they admitted us and told me that the baby had a stomach problem. Since we had given him some cough medicine and he had not recovered, they said that they should admit us so that he may meet the doctor to check if there is any other problem. He was admitted to the hospital. Since it was the second time, the doctor examined him, they tested his blood, placed some measurements, and said that he had no problem other than cough. He was not breathing well because of a cough. So we were admitted and stayed, they examined him two days later, and found that he had no other problem. We were discharged and went back home.

**MODE: Okay, proceeding on this point we are discussing, can you tell me about your experience with the child when you were at home?**

W1: Sorry, come again?

**MODE: Can you tell me the experience of living with the child after getting discharged? How it went**

W1: I just live with the child, would there be something exceptional that would happen?

**MODE: Since you were with the child during his sickness, you can share with me the journey of how it was when you were at home, and how you took care of him every day. You may state it in that way. You understand that living with a sick child is not the same as living with a healthy child.**

W1: Yes, living with a sick child cannot stop one from feeling worried. By then, the baby could cough and scream, and I could feel scared, but was there any other choice? I felt scared and saw that he was hurting since he would breathe and scream, and you could also see that he was hurting. I could try my best and deal with him. He coughed and I took him for treatment, and 5 days later, the medicine was not effective. The first one had exhausted and they had given me another one. So I immediately decided to take him back so that they exchange the medicine.

**MODE: Hmm**

W1: You understand that I also was not secure because he could cough and cry because of feeling pain. As he cried, the cough increased, but a time came and the cough eased, and he became normal.

**MODE: Okay, did it have an impact on your finances or cause mental/physical depression?**

W1: On the finances, it really changed because I used a lot of money, but the depression was inevitable, so what would I have done? For the funds you understand, the money one spends in the hospital is too much, and the financial situation had to change as well. I had livestock; I sold the animals till I was left with none. I did not have a cow, now I rear someone else’s cow because I sold my cow to pay tuition fees of a student. I sold the goats and it was not sufficient. It became necessary that we sell a piece of land to keep treating the baby. It was because we were going to pay for the exams, photocopying, and it was a lot. The hospital was charging ninety something ..., I do not remember exactly because we didn't pay for it in one instalment, we first paid the first 63,000, and then we paid again, but I don't remember the amount.

**MODE: Hmm**

W1: You understand that we backslid financially, but since I am seeing the child, there is no problem with that.

**MODE: Comparing his case to other children, do you have other children?**

W1: Yes, I have other children, this one is the sixth.

**MODE: Ooh, that one is the 6^th^?**

W1: Yes

**MODE: Most the people I talk to experience this in their first birth, I was also thinking that you have one child.**

W1: Hhhh no, he is the sixth, my first born is 21 years old.

**MODE: Ooh, you have mature kids**

W1: Yes

**MODE: Comparing this to other children, you have experience in giving birth. Referring to other children you have given birth to, what difference did you observe in raising them, in giving birth to them since they all have different lives?**

W1: Some of my children did not have a good life. Like the eldest I gave birth to, she had worms that devastated her stomach. It was a hard experience because I could breastfeed her, and she could immediately vomit. When I started feeding her food, it could be the same case. Since she was used to vomiting, it could detard her growth, and she did not grow well. But now he is a young lady, you cannot even know she was born with those worms.

**MODE: Hmm**

W1: The second born is also a girl aged 19, she lived and did not experience the worms that cause vomiting, but she grew up with a slim body, but the experience with her was not hard. The child that made me cry the most was the third born, he….

**MODE: Wait a bit, we are having network issues. Hello? Hello?**

W1: I can hear you.

**MODE: Okay, tell me, I can also hear you. I was having network issues.**

W1: Yeah

**MODE: Hmm**

W1: I was talking about the third child, he was not a burden, and it was the same case with the fourth and the fifth. The one who made me cry was the fourth and the first born, plus this one who is related to the topic. Since I could take other children to the nearest hospitals and they get better, I would say that they were not that problematic to me. This one was the most tiresome.

**MODE: Hmm**

W1: To an extent that the neighbors were like, “Ahaa! Why would a grown-up person want to bear another child? Let her face the consequences.” So, when they see me taking the child for treatment, they say, “let her feel it since she is the root cause.”

**MODE: HHHH**

W1: And I say that though he might be a burden for me, when God comforts me and I send him to bring water, I will feel at ease.

**MODE: You are not the root cause, aren’t there people who give birth to 10 children, and they all come out healthy? That was just meant to be.**

W1: Yes

**MODE: It has no relation with saying that you were old and had given birth to many children.**

W1: HHHH, they just make jokes about me.

**MODE: No, just leave them**

W1: Yeah, I also left them.

**MODE: You see that when someone is pregnant…**

W1: Hmm?

**MODE: When someone is pregnant, there is a way they think about the child that they will bear, and you ask yourself a lot about him. How will he be? You know such parental concerns. When you had that child in your womb, what would you be thinking, or what would you be planning since you did not know that she would be born with this situation? How had you thought about upbringing him? What had you planned in mind that changed after realizing the problem that he had?**

W1: How I would upbring him, the upbringing in the rural areas is the usual one, you just bear him and find clothes, you start breastfeeding him, are there any other special things?

**MODE: Speak louder**

W1: Apart from preparing his clothes, are there any other preparations that you can make while pregnant?

**MODE: No, how can I explain it? When you are pregnant, you say that you will take care of your child when he is born, you will feed him, and he will grow well. Every parent has a plan for a born child.**

W1: In the village we do not prepare a lot of things because you find it normal. Another thing is that we are not people with enough money to do extraordinary things.

**MODE: No problem. Can you share with me how it changed your relationship with people from the society, in your neighborhood, or your community? In your village where you stay, how did they receive the news? How did it go?**

W1: How they received me when I came from the hospital?

**MODE: No, how they received the news that you gave birth to a child with external intestines**

W1: Ooh, they said that our home had encountered a tragedy. But now they all heard that I brough him alive from the hospital and they were like, “Ooh, God is powerful, is it really possible?”

**MODE: Hmm**

W1: The neighbors were startled. Now there is no problem. They kept coming to see how the baby is doing. Yes, now there is no problem, and we are on good terms with our neighbors.

**MODE: Okay, how about your relationship with your husband? How did it go?**

W1: Nothing, it is just normal. It did not change him to result in disturbance or depression. He also accepted the situation, and he has no problem.

**MODE: Are you still together?**

W1: Yes

**MODE: In all those moments when you gave birth or when they told him that his wife gave birth to a child having a complication with external intestines, how did he receive the news? What did he say?**

W1: HHH, he is right here.

M1: Hello

**MODE: Yes**

M1: Let me be the one to tell it to you.

**MODE: Hmm**

M1: Hello

**MODE: I am hearing you, tell me**

M1: I was around when he was born. I saw that we gave birth to a child with a problem. But in collaboration since I am married to this woman legally and religiously, the relationship of a couple should be sticking together through the thick and thin.

**MODE: Hmm**

M1: That is why I had to search for money starting from my livestock, and I even sold a plot of land that was beneficial for us, so you understand that we are together till God’s will shows us His plans.

**MODE: Yes**

M1: Yeah, thank you.

**MODE:** **Yeah, thank you so much, you may put her back on the phone**

M1: Yes

**MODE: Hmm**

W1: Hello

**MODE: Thank you for sharing with us your life experience, let us talk about how the child is doing right now. How is the child doing? How is his health?**

W1: How he is doing right now?

**MODE: Hmm**

W1: He is doing well, and breastfeeding. He does not defecate well ever since I started giving him the prescribed medicine during the appointment. He defecates and then spends 3 days without defecating. I am just wondering what may be changing in his life.

**MODE: Hmm**

W1: Otherwise, he is doing well.

**MODE: He has no other problem**

W1: No problem

**MODE: On the side of diet and breastfeeding, does he breastfeed well? Does he only breastfeed, or you breastfeed him and give him milk? How does it go? How do you feed him?**

W1: I only breastfeed him; breastmilk is sufficient for him.

**MODE: According to your observation, does he breastfeed well with no problem?**

W1: Hmm?

**MODE: According to your observation, does he breastfeed well with no problem?**

W1: Yes, he breastfeeds with no problem.

**MODE: Okay, how about his growth?**

W1: His growth?

**MODE: Hmm**

W1: When you say his growth, you mean?

**MODE: In height, does his weight increase? His life in general**

W1: They have not yet measured his height, they measured on the weighing scale only, the weight does not increase that much, but since he used to have little weight, as I relate to moments at CHUK, they measured when 1.900kg he was born with had dropped to 1.800kg

**MODE: Hmm**

W1: Yes, but when he was discharged, he was already 2kg and some more grams, about 80. So, I went back home, and a community health worker came to measure his weight. 2 weeks later, he measured 2kg and 250g, she came back 2 weeks later and he had 2kg and 500g. In those 2 weeks since he suffered from cough, he could cough and refuse to breastfeed, and the cough backslid his progress for 2 weeks, and he stood there like a ….

**MODE: Speak louder**

W1: 2 weeks after catching cough, it dropped and he no longer gained weight, but you understand that in the first two weeks he had gained 500g when he came out of the hospital. When he caught a cough, it backslid his progress, and they measured him later when he recovered from cough, and he had 2kg and 800g. Now he has 3kg. Since it is exceeding, you find that the cough has backslid his growth, because if he had not suffered from a cough, he would be having 3.500kg, but he seems to be healthy.

**MODE: Hmm**

W1: Now he breastfeeds, he has no problem. When you look at his appearance, he is a bouncing baby boy.

**MODE: So, you told me that he cries frequently, do you see any other pain that may be affiliated with that?**

W1: He usually has stomach pain, but it does not occur frequently.

**MODE: How does he contract that pain?**

W1: Like when he is breastfeeding and he stops, then he cries. He stretches his leg, and you see that he is feeling a stomach pain. A child experiencing pain is not hard to notice, he could try to stretch his legs, and turn a bit, it could last for like 10 minutes, but he could later calm down.

**MODE: Didn’t you take him to the hospital to explain the problem that he has?**

W1: That one of experiencing pain?

**MODE: Yes**

W1: I did not tell it to the doctors because all the babies usually experience that, I observed and found that it was something normal. Since it does not last for long, I observed and realized that it will just recover by itself like the way it goes with others.

**MODE: Since you do not know what it is, I would advise you to find out its root cause, right?**

W1: Yeah

**MODE: It should not result into something else. I am not saying that he has a certain problem, but it would be better if you realized that it happens frequently to go and find the root cause.**

W1: So, if it happens again, I should take him to the hospital?

**MODE: If you see that he is not crying that much, I am not telling you this as a doctor, I am telling this in reference to what I would do if it were my child.**

W1: Yes

**MODE: If I saw that he was crying a lot, I would take him.**

W1: It is not that much, it takes him a little time, if I took him, I would not reach at the hospital when he is still crying. I would reach there when he has recovered. However, I would reach there and tell them what happened.

**MODE: Yes, did they give you any appointment to return to CHUK?**

W1: They gave me an appointment, I returned on 24^th^ May.

**MODE: What did you find out?**

W1: They tested him and found that he had no other problem, they prescribed medicine and I went home.

**MODE: They prescribed a medicine of bringing what effect?**

W1: What?

**MODE: They prescribed a medicine of bringing what effect?**

W1: They told me that it is meant to clean the stomach.

**MODE: Yeah**

W1: The doctor once again told me, the one who had received him. He told me that he will set an appointment in November, that I should go back there on that date when I realize that a child has a problem, and when I find that he has no problem, he gave me a number to contact and tell him that the child has no problem, so there will be no returning.

**MODE: It is in November, on which date?**

W1: Unless I go to check on the papers, I did not memorize the date.

**MODE: Okay, no problem. Does he experience diarrhea or vomiting?**

W1: He does not usually experience diarrhea and vomiting also happens rarely. Sometimes he pinches fingers on the throat and vomits, but he does not vomit that much.

**MODE: His vomiting is not related to the disease he had at birth?**

W1: It is not related; he does not vomit that much.

**MODE: Okay, that is nice. Can you tell me something you wish to have been told before giving birth to your child, right?**

W1: Yeah

**MODE: You see when you were pregnant before giving birth, what do you wish to have known concerning the sickness of your child, maybe because of the sickness he endured at CHUK, the time you spent there, and you say, “if I had known this, it would have been better for me.”**

W1: If I had known that the child is like that?

**MODE: No, maybe that he would be born with that disease, you see, there is a time when something happens, right?**

W1: Yeah

**MODE: And when it happens, you say, “God, if I knew it, I would have done this or that.” Like when a person makes a flashback and regrets not knowing the information. Which information do you wish to have known about the condition of your child before his birth? Or before this happens?**

W1: Like the information I would wish to know before I give birth?

**MODE: No, you see that you later went to CHUK, right?**

W1: Yes

**MODE: After the CHUK experience, you saw the child’s birth, and according to your experience and the lessons that you learned, and all the moments that you went through, right?**

W1: Yes

**MODE: What did you find out now that that you wish to have known a long time ago? Whether the information about the sickness of your child so that for the next time if you are to get pregnant, you would be aware of that disease?**

W1: What pleased me the most is that I saw this disease at birth and it was unusual, but God also prepared skilled specialists, so after all those things, what would I wish for?

**MODE: I do not know, maybe you can tell me**

W1: I would wish that there is a school that keeps training doctors and that we may have more specialized doctors in treating this disease because I thought it was impossible, and I would wish that among the students who are learning, there may be specialists who can do that.

**MODE: That is your wish. That is the information you would have wished to know?**

W1: Hmm, I would wish that we may keep having people to teach so that we may keep having more doctors because had it not been the doctors, this child would not have been alive. He would not have reached this very day.

**MODE: When you were at CHUK with the sick child, what challenges did you face?**

W1: I had no challenges when I was at the hospital.

**MODE: You did not face any challenge?**

W1: Challenges? I guess I did not face any. I could see the doctors taking care of the child, and I could encounter benefactors who stepped in to assist people. There was no concern at the hospital, basing on how the doctors take care of the patients. I did not face any challenge at the hospital since they follow up on you minute by minute, and even when a child faces a challenge, you tell it to the doctor. The doctors there are very attentive since they do not all leave the room at once, one leaves and the other stays, and in case there is any change, you can easily alert the doctor. They are also looking at them every time since they are children in critical conditions, they have a spirit of sacrifice. Oh God, they do whatever it takes and take care of you.

**MODE: What were your best moments at CHUK?**

W1: Best?

**MODE: The best moments you had. You see that in life we face good and bad times. During that period of one month, what good things did you see to an extent you would say, “I thank CHUK for this and that, I achieved this, and these were my best moments?”**

W1: What I really appreciate is that I have seen doctors who care for the patients. I saw those benefactors coming to help, especially very poor patients who cannot afford to buy food. There were also benefactors who also come to help and bring porridge in the morning, food at noon, in the evening they bring food again. I saw some good things in CHUK, I had no worries. The only concern was the way we were searching for money, calling home every now and then, money, money. However, the baby received care at the hospital, and I was also given food. I was also buying other things to be able to get breast milk, but it did not happen that much. It is good to get treatment from CHUK, they take care of the patients.

**MODE: As you had your sick baby there, there were also other mothers. What would you say that you learned from them? Which moments did you share? How was your life like in that hall where you stayed?**

W1: We could discuss and everyone could talk about her life, the problem most of us were worried about was getting payment for the hospital bills. We could socialize with other parents, some children would die, and life would continue. We could see a dead baby and worry, but since God has his own timing, there were rescuers and we had them, but the dead were also many.

**MODE: What was the cause of their death?**

W1: Their sickness

**MODE: Since you were there, maybe you know what caused their death, what would be the cause? Was it failure in breathing? Or it had not been possible to fix the intestines? What causes the death of a child born in such a condition?**

W1: Some people could reach there and they could tell me that the intestines of their children have been infected.

**MODE: Infected?**

W1: Yes, there were some people who were taken back to their local hospitals via ambulances because their children had infected intestines and there was nothing that they could do about them. After returning them to their homes, they could tell the parent that the child cannot manage to live, and they could send them home to wait for the following events. They could tell him/her, "transporting the corpse from here can be difficult, but let us help you, go and wait at your local hospital", and explain to him/her that the child has no chance of surviving, and add, "it is to help you wait for his day."

**MODE: Yes**

W1: Yes, some could even die from that hospital, some even died after getting their intestines fixed. Some had their intestines fixed, and the child could…

**MODE: And the child could do what?**

W1: Have a swollen body, the belly and the legs, and the child could die the following day.

**MODE: Ooh!**

W1: But most of the children could have swollen bodies after getting their intestines fixed, my child also had a swollen body, the thighs swole, and the belly swole, especially the part above the covered area. I was scared, but it lasted for 2 days and he recovered, and he became better.

**MODE: What reasons did the doctors give as to why children swell after having their intestines fixed?**

W1: They told me before fixing them inside the belly. So they told me that if you see a baby swelling, don't be afraid. They told me that every child whose intestines have been fixed usually has a swollen belly. So I saw the belly of my child swelling and I did not feel scared, luckily it had not swollen that much. So 2 days after swelling, it started reducing, the baby became better. But there was someone whose intestines were fixed like today, the body was swelling, and in the evening he died. I guess everyone has his own time.

**MODE: So, what message would you give to a parent who gave birth to a child with a similar condition like yours?**

W1: When I went to the appointment, I arrived and found parents with children who had a similar condition. When they saw me, I went straight to the room where we received treatment because I did not know where I was going to go to ask the doctors where I would be received. The parents who saw me asked me, "what are you doing here?" I told them that I had come for an appointment. They asked me, "have you brought a patient here before?" I said, "yes, I had a patient here, I have been at home for a month, and now I am coming for an appointment." So they said to me, "What was your son like?" He had external intestines. The parents present there were also depressed. They said, " since they treated your child and he recovered, now you are back after a month and the child is still alive?" I responded, "yes, he has healed", so they asked, "how does his belly look like?" I showed them and said, "don't you see that it is just a scar?" They responded in amazement and said, "these are just kids, and they can be treated in a little while." So, I comforted them. They asked, "will our children also recover?" I told her that I also came depressed but now you see that there is no problem. I refuse to say anyone you look at and see no problem. So when parents give birth to a child like mine, I have to comfort them because I have seen that nothing fails God.

**MODE: Hmm, nothing at all.**

W1: Hmm

**MODE: Our conversation is coming close to an end. Is there anything we have not discussed that you would like to share with me?**

W1: Something we did not discuss?

**MODE: Yes**

W1: Nothing, I just keep wondering if the child will have a navel. I see that the navel is not appearing so that one may see that he has a navel just like other people.

**MODE: How does it look like?**

W1: It is just a scar, and something tiny that would like to close. I keep wondering and I do not know if he will have a navel like other people. There is a hole in the place where the navel is supposed to be. The place where the hole is found is where there used to be the external intestines, so it is just a scar since they covered till the hole was no longer visible.

**MODE: I can ask that question for you, isn’t this your number?**

W1: Hmm?

**MODE: I can ask that question for you, since I am not a doctor. I would be lying if I were to say that after a while the navel will be like this, but I can ask for you, and give you a response, and you would remind me in case you see that I am delaying, right?**

W1: Yes

**MODE: I can ask this question to the doctor based in Kigali**

W1: Yes

**MODE: Do you have any suggestion or question before we wind up?**

W1: I do not know your name.

**MODE: Ooh, you may have forgotten because I told it to you. My name is Gisele Tumukunde**

W1: Ooh, maybe you told it to the husband, you had not told me.

**MODE: Ooh, the other time I told it to you, I felt that you were together and thought that you were listening**

W1: Ooh, I had not heard it. Thanks

**MODE: Yeah**

W1: I was curious to know your name.

**MODE: Yes, thank you so much. May God bless you.**

W1: Likewise

**MODE: Thank the husband on my behalf, thank you for your time.**

W1: Yes

**MODE: Alright, thanks and have a nice day.**

W1: Yes
