# Supplementary material for: Exploring the lived experiences of parents caring for infants with gastroschisis in Rwanda: The untold story
Source: PLOS Glob Public Health. 2022 Jun 15;2(6):e0000439. doi: 10.1371/journal.pgph.0000439 (PMC10021215; doi:10.1371/journal.pgph.0000439)
Supplement: S1 Data — (ZIP) [file pgph.0000439.s002.zip › S1_Data/S7_Text.docx]

**BB 7- English Transcript**

**MODE: Hello**

W1: Yes

**MODE: You are still on the call?**

W1: Yes

**MODE: Thank you for accepting to talk to us, the objective of this interview is to find out how your child was taken care of at the hospital, and how you also took care of him, right? We would to utilize this information in helping CHUK hospital to make changes in service delivery, but we won’t reveal your names or your identity to anyone.**

W1: Yeah

**MODE: When was your child admitted to the hospital?**

W1: When they treated this disease or other diseases?

**MODE: No, when they treated this disease after giving birth to him**

W1: On 26^th^ March

**MODE: Which year?**

W1: This year, 2021

**MODE: Okay, what was the span between giving birth and taking him to the hospital for treatment?**

W1: I gave birth to him at 5:00 a.m. and we reached at the hospital at 6:00

**MODE: In the evening?**

W1: Yes, I gave birth to him at the health center at 5 a.m. in the morning, they immediately transferred to the hospital and we reached there a few minutes past six. We reached at CHUK around 5 p.m. in the evening as the night was coming.

**MODE: Okay, you gave birth to him at Nyagatare Health Center?**

W1: No, the one of Nyakigando

**MODE: Nyakigando is located where? Is it Nyagatare?**

W1: It’s in Katabagemwe

**MODE: Katabagemwe, then they took you to which hospital?**

W1: Nyagatare Hospital

**MODE: How did you get to CHUK?**

W1: Nyagatare Hospital transferred me to CHUK.

**MODE: On that very day?**

W1: Yes, on that day he was born

**MODE: So, you told me that he was born on 26^th^ March 2021?**

W1: Hmm

**MODE: Okay, how long did you stay at CHUK?**

W1: I stayed there for three weeks and a half.

**MODE: How old is the child?**

W1: He is 2 months and a half, and he’s turning 3 months on the 26^th^ of this month.

**MODE: Okay, he’s a boy or girl?**

W1: He’s a boy.

**MODE: Okay, do you have any question before we start?**

W1: I have a question.

**MODE: Yes, ask me.**

W1: My first question is related to the research that you do or one’s participation in it, does it require charging people money since I cannot get it?

**MODE: No, it doesn’t require you anything. It’s only for this time, and later basing on the information you will give, there won’t be anything else after this. We are just calling you to know your experience at the hospital. It doesn’t require you to pay for anything.**

W1: Another question I want to ask, hello?

**MODE: Yes, I can hear you.**

W1: Another question I would like to ask, for these children, do they face any consequences since I have been recently in the hospital?

**MODE: What happened to him?**

W1: He was coughing, then it aggravated to pneumonia

**MODE: It aggravated in which way?**

W1: Suffering from pneumonia and breathing while panting.

**MODE: Ooh, I am not a doctor, but since I spend time with them, when we finish the conversation, I’ll ask them your question, and remind me in the evening, right?**

W1: Yes

**MODE: Remind me and I’ll give you a response. I’ll ask them if it causes any side effects, and I’ll let you know if they have responded to me. No, call me tomorrow, remind me tomorrow evening, maybe that’s when they have their free time, right?**

W1: Yeah

**MODE: Now we can start?**

W1: Yes, we can start

**MODE: I would like to start from the moments you experienced at CHUK on 26^th^ March, you and your child. You were at CHUK, is it true?**

W1: Yes, we were there, on 26^th^

**MODE: Yes, can you tell me in details what happened that time when you were at the hospital?**

W1: What they did for him?

**MODE: How did it go when you reached there? Share with me the journey of what happened you got to the hospital, how the situation was, and how it went**

W1: The way I was received?

**MODE: Yes**

W1: I reached there, when I reached at CHUK, they took us to the emergency room. In the emergency room, they checked to find out the care that he had received from the health center, then they tried to put him in an incubator (the device with a light that adds warmth). They searched for it and the lamps were not found, then they took him in pediatrics’ room. When we reached there, in that very night the doctor came, in the incubator where they had placed him, they got a bag and placed in the lights, and prepared them well. After preparing the lights, they put them at the top of the incubator. As he breathed, they lowered, and when morning came, they found that the lights had displaced, and they tightened them. The next day on Sunday, they brought in all the lights till they finished, and it took about 3 days. They used the lights till they went off, and it was on Monday when they got over. They later covered him. After covering his intestines during those days of sickness, they also stated that he had a blood infection. I spent there some days taking care of him because of that, they covered and uncovered the wound, bathed it. In the fourth week of the month, that was when I was discharged and they told me that I would be going to the health center for wound coverage.

**MODE: Hmm**

W1: Yes

**MODE: Apart from these things that happened where they fixed the intestines and told you that he had an infection, in those 3 weeks, did you get any service or obtain further information? How was it during that whole time which was almost a month?**

W1: Almost a month?

**MODE: Yeah, how did it go? What kind of support did they give you? Which information did they give you about the status of your child when you were at the hospital?**

W1: Early in the morning, the doctors could come to see us since we spent the whole day with nurses and we could also spend the night with them, but in the morning, the doctors could come to us, ask us how the children are doing, if they got any other diseases or complications. You could tell them how your child is doing, and they could explain to you that the problem he has, or let you know that he is okay, and that the treatment he received was effective and he has no other problem. That’s what they could tell us, and in case they have changed medication, they could tell it to you basing on the sickness that he has acquired.

**MODE: Okay, let me come back. My airtime is about to get below minimum balance, let me recharge and call you.**

W1: Yes

**MODE: Yes, keep on telling me.**

W1: Yes, in the morning when they came, I could tell them how the child is doing, and if he has got other complications, and if he has no complications, they could know it, and if he had any problem, they could conduct a blood test, but he also had a problem that his blood was not clotting, and he had liquid blood. They had to transfuse, I don’t remember how they explained it to me, but they did it to ensure that his blood clots.

**MODE: Hmm**

W1: Yeah, so he had no problem other than the blood infection that they treated. The infections caused my delay, but the condition of being born with external intestines had already been treated and he had healed. I had to leave a week later.

**MODE: Hmm**

W1: Yeah

**MODE: Okay, so what was your action when you saw that your child had external intestines as you were at the hospital, when you gave birth to a child and found out that he had external intestines?**

W1: After giving birth to him, they hid it from me, but they told me that I had given birth to a boy. Since I was already a mother, they also called my husband urgently and when he arrived, they first set some preparations for me, and when the baby was out, that’s when they told me that I gave birth to a child with a problem. He is a boy but he has a problem of external intestines. I felt depressed and thought that my child is meant to die. That was when they transferred us to Nyagatare and when we reached there, at Nyagatare they didn’t tell us many things, we just had to wait for the ambulance that transported us. They didn’t do anything for him at Nyagatare, they just adjusted to what had been done at the health center, prepared them and put there cotton to protect them from damage. We left Nyagatare for CHUK, they are the ones who removed those things, and placed them in the appropriate bags. We reached there when I was thinking that it’s all in vain since they were requiring a lot of funds. I started spending money at Nyagatare and seeing that it was all in vain, and that my child won’t make it. After raising them, the doctors came the next morning to discuss the issue with me and told me that for the children born with that condition, they use their knowledge and treat them, when they treat them and they recover, then there is no other problem. Later, another doctor came to talk to me and after talking to me, he told me that those children do not have a disability or I shouldn’t think that it’s witchcraft, and that these are cases that they are used to seeing. I asked them if they helped deliver children with a similar condition whom they treated and recovered, and who have grown up to become mature. They told me that in the past five years most of them died, but they told me there were others who had come for an appointment for checkup to see the status of the child, and they told me that it’s been a year and half that this child left the hospital. Then I started believing that my child will also live. In those first three days, there were parents I had found who had also come with a similar issue and I found they had already fixed it for them. Those parents told me, “See how my child is doing, you can see that mine has already recovered. I also came here with a similar problem, and there are some who were discharged, and those who died.” So I started accepting it and pleaded God to assist, where the doctors have done their part, God also has to play his part.

**MODE: Hmm**

W1: I started feeling that my child will live when they were done fixing them inside.

**MODE: Hmm**

W1: Yes

**MODE: Was it the doctors from CHUK who told you that? Or it was those from Nyagatare?**

W1: No, it was those from CHUK. The ones from Nyagatare didn’t tell us anything other than reaching there and waiting for the transfer of the vehicle that transported us. At Nyagatare, they didn’t say a thing.

**MODE: Hmm, did you ask them the root cause of this disease? What does it take for a child to suffer from it? What did they tell you?**

W1: I asked the doctor for the cause of the disease and he said that one can’t say that it was witchcraft or say that neighbors poisoned you or say that it is a disabled child. They told me that he is not a disabled child and none poisoned me. He also didn’t a find a way to explain that it was a problem of the mother, I don’t know. He didn’t give me sufficient explanations.

**MODE: What were the challenges that you faced in that time?**

W1: The challenges I faced in that time when I took him for treatment at CHUK?

**MODE: Hmm**

W1: The challenges I might have faced is that every test required money, and sometimes I didn’t have it. Since the husband had stayed at home, he could do his best and send whenever he got it. I feel that I didn’t face any other challenge when I was at CHUK other than the challenge of money that they were charging me.

**MODE: You were with many people such as mothers with children that had this challenge.**

W1: Yes, there were also other mothers that left since after my arrival, there came other 6 children, some died and others survived. I think that three of them died. I saw three dying, and I left the other hospitalized.

**MODE: Did you face any mental challenges apart from the financial constraints? How were you feeling during that time basing on the conversations that you had with those mothers since you were all together? Money is a common problem that may burden everyone, but other challenges like your thoughts or the way you felt in your heart, how was it?**

W1: When I reached there, I found that the ones they treated were about to get discharged and their children were healthy. So, they comforted me and I also accepted the situation. I felt that they were going to treat my child and he recovers. That’s when the doctors came to me and told me, “Unless the child faces another problem, you can see that we treated his sickness and he has recovered. Keep checking on the wounded place such that germs do not enter that may result in sickness. So I accepted my situation after seeing that there are people who came the same way and recovered. Since then, I had no concerns about that disease.

**MODE: When you treat them, do you use CBHI (Community-based Health Insurance) or you have a private insurance?**

W1: We use CBHI and even though it’s CBHI, one is still charged a lot of money, but they told me that I leave the place while having cleared the bills of 100,000Rwf. The one who is not under CBHI pays for 1,000,000Rwf.

**MODE: Ooh! Okay**

W1: Yeah, but you can’t go there and cover bills less than 50,000Rwf. When you’re there for 3 days, the bill is already 50,000Rwf.

**MODE: Apart from the challenges that you encountered, during that time at CHUK, was there any good thing that you learned from there? Putting aside the fact that you were sad with a sick child, was there something good that you saw/gained? How was it?**

W1: The good thing we gained from there?

**MODE: Yeah, or anything that you experienced at CHUK**

W1: How would I state that these were the good and the bad? The good thing is that I left with a healed child, and others also left with healthy children. There were also others who left without children. The good thing is that I left with a healthy child.

**MODE: Yeah, for the services that they gave to you, you didn’t like them? Or they were just fair and tolerable?**

W1: No, I liked the services because every hour there is a doctor to take care of him, every hour a child has to get medication. The services I got there, I never found them anywhere else. I was about to end my 2 weeks stay at Nyagatare, but the services I obtained from CHUK, I didn’t obtain them from Nyagatare.

**MODE:** **Okay, after getting discharged from the hospital with your child, which plan did they give you for taking care of the child?**

W1: The plan for taking care of the child?

**MODE: Yes**

W1: They gave a plan for taking care of him and told me that when we reach at home, I shouldn’t bathe him by placing him in a basin, and that I have to wipe him, protect him from wind, and protect him from being carried that much since his health has not yet permitted him for that, and I had also given birth to him when he was only 8 months. They said that I have to protect him against all those things and also avoid carrying him on my back.

**MODE: And you will carry him after how long?**

W1: Hmm?

**MODE: You will carry him after how long?**

W1: They told me that I have to carry him after seeing the progression of his wound. After seeing that it has merged and that he was sick in those days, I started carrying when they were putting there a tiny cover.

**MODE: Didn’t they tell you to bring him back for another appointment?**

W1: Yes, they gave me an appointment after a month. I came and spent a month, and I returned. After one month, I went back there and met a doctor. He observed the child and said, “I can see that now you have grown up and increased in weight. The weight you had when you were here has doubled in a period of one month, there is nothing else that we can do for you.” The cover that was there, the doctor removed it and said, “go back home, there were no other changes”, and we thanked so much.

**MODE: You brought him back to CHUK? That’s where your appointment was meant to take place?**

W1: Yes, the appointment was at CHUK. They gave me an appointment and I went there. After seeing the doctor, he said, “I can notice the child’s growth, he has increased(doubled) in weight. I can see that you are doing well, I cannot give you any other appointment, but in case there is a change, you can reach out to your hospital and they can call us. But with regards to this sickness, I can see that he has no problem. Go home and keep breastfeeding him well and eat well.”

**MODE: Hmm**

W1: Yeah, that’s what they told me, and nothing else.

**MODE: What would you say about the services that they gave you when you came for the appointment?**

W1: The services I received when I came for the appointment?

**MODE: Yeah**

W1: They gave me good services. After reaching there on the appointment day, I first lost track and went in the wrong place. When I reached in that wrong place, I met with a doctor who checks for patients that come on appointments of diseases that are different from ours, when I told it to that doctor, he told me the direction I have to take, that I’m going in the surgery section, and that my appointment is taking me there. That’s when I went there and asked, they told me where the surgery section is located, and I went there. They told me where I have to go and pay the money to get a number. I paid and they gave me the number as I sat on the waiting line. After giving me the number, I went back to a place where they wrote my names and my identification. After writing that, they told me where I have to go and sit while I am waiting to meet the doctor. That’s when I got on the waiting line and later entered to meet the doctor. When I reached there, he looked at the child and didn’t do anything else. He just looked at him and stated that my child is in good health. I asked him, but I am still seeing the wound cover, when will they remove this cover? Or when will it recover? They uncovered it and he observed, after observing, he said that it was just a tiny thing. He removed it once for all and stated that it will recover as a result of bathing him, and after bathing him, you cover him with a blanket. And you also let some air access the area.

**MODE: Did you like the services that you received?**

W1: I liked it, it pleased, I didn’t disapprove with it.

**MODE: When you were at home, was there any emergency situation that made you seek immediate medical care for your child? Like an emergency that caused you to take him to the health center during that time when you were at the hospital?**

W1: There was no other sickness. After getting at home from CHUK, we reached there and the first thing I was told is to take him for wound coverage in 3 days. I should go there like today, miss the next day, and go back the day after tomorrow. I went and when I reached at home, I missed that day since it was the day he had been covered. I went there on Wednesday and they cleaned it. But the services I got from them, I didn’t like them because of the way they were cleaning him, it wasn’t pleasant like the way it was back in CHUK.

**MODE: How did they bathe (clean) him?**

W1: They could uncover him and take the absorbent cotton. After taking the absorbent cotton, they could put there the cleaning medicine and remove any disinfectants and things like pus. They could clean it and after wiping the area, they could cover him. But the doctors who covered him didn’t do it well except one doctor who did it like the way it was done at CHUK. And on the day he took an off, I would be displeased with services from others but I could accept it with no problem.

**MODE: Proceeding to what you told me about the experience of living at home with the child**

W1: I can tell you what?

**MODE: What was it to live at home with the child? When you are sick with an unusual disease, what was challenging?**

W1: When I got back home, I didn’t do any other job because I reached there and he could weep a lot. I was required to do the home chores because we are farmers, I never went in the field. I returned there after his recovery. They could get there and bring food, or they could go to the market, bring it and I prepare it. My young sister could cook the food. It took long for the child to recover. It reached a point and she was, I would say, tired. After seeing that she was tired of cooking for me, the husband started carrying the child to prevent him from crying since the baby never slept; he could cry a lot, but when you held him in your hands, he could calm down. The husband could come in the evening during the time of cooking and he could carry him. I could first cook and carry him later after cooking. But when I came from CHUK, I wouldn’t leave him and the breast was ever in his mouth. Whenever the breast left his mouth, it required carrying him with your hands.

**MODE: What financial challenges did you face when you were at home? Like in taking care of him?**

W1: The challenge I had is that he cried and I could think that his crying would result into something else. I started thinking that maybe they might have discharged me when he has another problem, but my heart could tell me that my child has no problem, and I would instead choose to protect him against any obstacle.

**MODE: Did it affect your mental health?**

W1: No, it didn’t affect me because he was breastfeeding and had no other problem, he cried, he defecated, he urinated, and it didn’t cause me any problem except trying not to make him cry to avoid any consequences, or I think that his crying is a result of being discharged when he hasn’t fully recovered.

**MODE: How many children have you got so far?**

W1: I have two and he is the second.

**MODE: Comparing him to the first born, what is the difference between raising them?**

W1: The first one was not challenging like this one.

**MODE: Like how?**

W1: This one was very challenging, I carried him in my womb while going to the hospital. That’s when I started getting treatment. When I gave birth to him, I took him for treatment and I’m still taking him, I haven’t stopped.

**MODE: You told me that when you were pregnant, you went for treatment. Were you sick by then?**

W1: Yes, I could feel uncomfortable and sick.

**MODE: How would it occur?**

W1: I used to experience dizziness and backpain, and headache. Even feeling that he is not active in the womb. That was the difference between what I had with the first born. I had no issue with the first born and I could go to the hospital for testing only.

**MODE: Comparing it to your expectations when you were pregnant, when one is pregnant, there are things that they expect. Comparing it to what you expected or what you felt when you carried him, can you tell me the difference between the feelings that you had when you carried him and what you had when you gave birth to him?**

W1: There is a big difference because I could take him to the echography, like when you get there and they determine the sex of the child, that did not happen.

**MODE: Why didn’t they tell you the sex of your child?**

W1: I went there for the first time when the pregnancy was 6 to 7 months, and they told me that he is seated and not upside down, and that they can’t see it. The second time I went there when I was 8 months pregnant, almost 9, they told me that he was playful and they also didn’t observe that problem.

**MODE: Hmm**

W1: They could tell me that he’s so playful in the womb but he could not open his thighs to show them.

**MODE: Okay, didn’t that cause you any problem? Or make you think deeply about certain things?**

W1: When I went there for the first time, they didn’t show him to me. I felt that since the child was not active in the womb, I never felt him when he was in the womb. I could think that maybe it’s not a child but an animal. That’s what I thought, but when I got there for the second time, that’s when they showed me the baby and I realized that I had a child in my womb. I was confused about the materials but the doctor told me that the highest probability would be a girl. He said to me, “go, you see that in this era, all children wear the same clothes. Go and search for boys’ clothes but the highest probability is a girl, and even girls can wear boys’ clothes. While you buy clothes, buy clothes for a girl.” I mean he said, “buy clothes for a boy.”

**MODE: Referring to all these that happened and you realized that you gave birth to a child with a complication, did it affect the relationship that you had with your husband?**

W1: It didn’t affect anything on that. Instead, it made him closer to me.

**MODE: Okay, what caused him to be closer to you? Were there specific reasons behind that?**

W1: The specific reasons behind that, he also thought that though the child was born, it would be a matter of time and he would die. As I told him how they took care of him at CHUK day and night, it motivated him to get the money that they were asking for. He could even borrow but obtain the money that they were charging. Even when we got back home, at first he thought that I wouldn’t bring him back, and when I got home, he saw that the baby was normal, he hadn’t seen him clearly. He saw that the baby had no problem and one wouldn’t know that the child was born in that condition.

**MODE: Hmm**

W1: As he saw, an additional factor was that he was a boy, it made him love him more. That’s what I saw as the reason that brought him closer to me.

**MODE: Yes, he loves boys?**

W1: Yes

**MODE: Okay, thank you for sharing your life experiences. How is the child doing?**

W1: The child has been sick and we have been in Nyagatare Hospital.

**MODE: How did he fall sick?**

W1: He started by coughing and I treated it. It even reached an extent when he wasn’t defecating, and I treated that. When defecating began, it was around 10 p.m., and the child started coughing, and he did not cry. He could want to cry but not manage, and breastfeeding stopped. I spent the whole night awake trying to breastfeed him, and he refused to breastfeed. As times went by, the situation aggravated, and he had a high fever. That’s when I spent the night awake and in the morning when I reached at the health center, they gave me syrup. I told them that I disapprove with syrup because it was the one I was using to treat him at home, and it has not cured him. Then they asked me, “for the problem of defecation that he had, didn’t he defecate?” I told them that he defecates once a day, when it’s not during the day, it’s at night. At the health center they were the ones telling me to take the syrup, he had even caught a flu and he wasn’t breathing well. They told me to take the medicine, I had gone there on Monday, no it was on Tuesday, he told me that if it gets on Thursday when he hasn’t recovered or started breastfeeding, I should take him back and get a transfer. I refused because as time went by, it aggravated. I kept pressuring them for a transfer or new medicine to buy at the pharmacy, and they refused to do it. But as time went by, he got worse, and crying even stopped. I went hurriedly at home and showed him the husband, we saw that the child’s temperatures had risen, he wasn’t breastfeeding. It was around 5 p.m. when I had left the hospital and reached at home around 3 p.m., we went back and reached there when it seemed like almost over.

**MODE: Hmm**

W1: So they pierced him looking for veins and didn’t find them, and another called for the ambulance as they put him on oxygen support and he regained life. When we reached at the hospital, they tested him and found that he had pneumonia and cough that he had before, but it was pneumonia that had worsened his condition. So, they were treating him and it only missed 3 days to complete 2 weeks.

**MODE: Now you went back home?**

W1: Yeah, I went there yesterday, you called me yesterday when I reached at home.

**MODE: Is the child doing well right now?**

W1: He’s still coughing but pneumonia healed. The infections that he had, they also told me that they discharged me when they had healed and I only remained with the problem of flu.

**MODE: Okay, praise be to God since he is recovering.**

W1: Yeah

**MODE: Apart from falling sick, does he breastfeed or you also give him milk?**

W1: No, he only breastfeeds.

**MODE: He breastfeeds well with no problem?**

W1: He breastfeeds well.

**MODE: He doesn’t face any other problems of suffering from diarrhea, vomiting, or having pain caused by…?**

W1: No, it was only the cough that caused him a problem. Now he has diarrhea, I don’t know if it was caused by the disease he had that I was treating nowadays, but he is having diarrhea nowadays.

**MODE: Does he still cry?**

W1: He no longer cries.

**MODE: Hmm**

W1: He cries just like any other child, when a child cries, it is a sign of life.

**MODE: His growth from birth till now in weight and height, how do you see it?**

W1: They haven’t measured all. They measured the weight when I took him to the hospital for wound covering. I left CHUK when he had 2.300kg, but now he has reached 5kg, I don’t know the grams because of the weighing scale we used whereby I went on it and carried him, and he could have 5 kgs. For the height, I can see that he is growing, he has no other problem.

**MODE: Does he play like other children? Is he joyful? How is he doing?**

W1: Yes, he is joyful especially after his recovery since he had started smiling, and when I covered him with bed sheets when he is not asleep, I could come and find that he has thrown the bed sheets, and when he slept , I could see that he slept while facing the opposite side. He has strength and he laughs, he is on phase of looking at a parent and conversing with him/her or looking at anyone else and conversing with that person. That is his phase, and when you breastfeed him in public, he stops and stares at them.

**MODE: Okay, that is really awesome.**

W1: Yeah

**MODE: After all these moments that you went through, can you tell me something that you wish to have known about the life of your child or the care that you needed before giving birth to him? Since at this hour you know a lot, you may say, “had I known this, I would have done this differently.”**

W1: Like if I knew the way he was when he was still in the womb?

**MODE: Yes**

W1: No, I don’t think about that, the way I saw it, I think that it was God who wanted to raise him. I felt that I can’t treat it in the womb and it recovers. So, I never pressured myself to think about that.

**MODE: What information do you wish to have known, that would have helped you more than the way it was when you realized that he would have that problem with regards to treatment?**

W1: I don’t think about it because if I had known that he was like that in the womb, it would have caused me depression. So, I never thought of what could have happened if I knew it before. But if they had told it to me when he was still in the womb, it would have caused me depression, and I would give birth feeling very depressed more than the way it was when I gave birth to him.

**MODE: Looking at the experience you went through, what would you tell a parent if she had a child with a problem like the one of yours? If you met with her, you know that when you see the child for the first time, you get shocked. If you met with her, what would you tell her?**

W1: If I met with a person having a similar problem like the one I faced with my child, I would tell her to feel that her child is normal like other children, that she should not worry since they can treat the condition of that child, and she would later not worry about the status of the child, like her child being like this (dull), and convince her that it’s a normal child. She should not think of him/her as someone who will be stupid, or who will die later. I can convince her that if the child had a sickness, it would be originating from elsewhere since I was convinced that it would not be caused by this sickness.

**MODE: Okay, thank you. Our discussion is nearing the end. Do you have any questions or suggestions to add on what we discussed before we close?**

W1: I have no question.

**MODE: Any suggestion or idea?**

W1: My idea is that after their growth as they reach 5 years, like in the next 5-10 years, the doctors from CHUK can send for us as people who had such complications. They can send for us and check them via the scanner to see if he won’t have any problem in the belly during his growth.

**MODE: Hmm, referring to what you went through, which service do you wish to receive after having known the birth of your child, wherever you are, what do you wish that doctors may do to change the way they receive you, or the way they care for your children born with that condition? Maybe something which didn’t go well.**

W1: A recommendation I may give to these doctors from the lower level who help in delivering the babies. There are those who die as a result of delay that causes infections and change in color.

**MODE: You mean the intestines?**

W1: Yes, when they change in color.

**MODE: They change to which color?**

W1: There are times when it changes to black, or it starts from green and ends in black, and it can be caused by their delay or putting on them absorbent cotton that lacks sufficient water. After delaying with them, the water in the absorbent cotton dries and he changes in color. I feel that after delivering a baby like that one, they should stop whatever they are doing and immediately transfer him/her to the specialized doctors because here they don’t have doctors capable of treating that condition.

**MODE: Hmm**

W1: Yeah, I feel like they should deliver such babies and immediately transfer them.

**MODE: Okay, thank you so much. We had an interesting conversation, thank you for your giving me your time and talking to us. The information you gave us will help parents who face issues like yours and also help CHUK in changing their service delivery.**

W1: Yeah

**MODE: Sure, may your child have a quick recovery, have a nice day, and thank you for your time.**

W1: Yes

**MODE: Do you have any unanswered question?**

W1: I have no other question, you told me that if I had a question, I would contact you on your mobile phone.

**MODE: Yes, save it and write Gisele. Remember to call me tomorrow, so that I may ask that or any other question that you may have, you may call me. And in case I don’t know the answer, I can contact for you those who are in charge.**

W1: Yes

**MODE: Alright, have a nice day and thank you so much. Thank for me your husband.**

W1: Yes, I’ll thank him.

**MODE: Yes**

W1: Yeah

**MODE: Have a nice day**

W1: Yeah, same to you
