# Supplementary material for: Exploring the lived experiences of parents caring for infants with gastroschisis in Rwanda: The untold story
Source: PLOS Glob Public Health. 2022 Jun 15;2(6):e0000439. doi: 10.1371/journal.pgph.0000439 (PMC10021215; doi:10.1371/journal.pgph.0000439)
Supplement: S1 Data — (ZIP) [file pgph.0000439.s002.zip › S1_Data/S8_Text.docx]

**BB 6 Transcript**

F: how are you?

P: We are fine

F; Yes

P: Hmmm

F: Hmmm, can you please remind me of your names?

P: My Names are…( The participant reminded her names )

F: Hmmm, yes [ The Name of the participant] …. Where are you from?

P: I am now located in Matimba sector in Nyagatare District

F: yes, thank you very much

P: Yes

F: F: My name is also Gentille Dusenge as I told you, and I am helping the researchers, Dr Samuel Kidane and Dr Semay Desta Shamebo

P: hmmmm

F: They are both master’s students at the University of Global Health Equity

P: hmmm

F: They are doing research on, on the lived experiences of parents of infants who were born with the disability of having their intestine outside and received hospital-based gastroschisis care) in Rwanda because you meet the requirements for inclusion into the study and can offer unique information regarding the question at hand in case you might decide to participate in this research. This is why we have consulted you. EEh , before accepting to join this project, you must understand and take into consideration the contents of this form, since it contains important information to assist you in deciding whether to participate or not to participate in this research.

P: Hmmmm

F: Hmmmmm, with that said,

P: hmmmm,

F: and, this project is being conducted as a core requirement for the Master of Science in Global Health Delivery at the University of Global Health that I told you about before, as said before it is called University of Global Health Equity or UGHE in short.

P: hmmm

F: Eeh,the project has received required ethical approval from UGHE and complies with international ethical standards for research to be carried out in Rwanda. Additional permissions have also been obtained from the CHUK and the IRB of the University of Global Health Equity.

P: Hmmmmm

So what else can I add, Participation is voluntary, whether you want to participate or not but it is voluntary not coercive

P: hmmmm

F; You could say that I really feel like I don’t want to or I agree.

So we are discussing the purpose of this study, when, or during the interview scheduling process (which will take place initially via telephone before agreeing upon a set time), the research team will discuss / or we are discussing the purpose of the study which is to explore the lived experiences of parents or guardians of infants who received hospital-based GS care in Rwanda. The project will help to describe the parents’ experience on hospital-based GS care for their infants, their perception of the health status of the infants, challenges faced and the health care utilization experience after GS care. The information collected will be used in understanding the biosocial aspects of neonatal surgical care and additionally inform the improvement of surgical neonatal care at CHUK. You are being asked to participate in this study because of the unique experience concerning your child’s GS care, both hospital and home based. I hope you spent time at CHUK when your baby was sick

P: Hmmm, we went and stayed at CHUK

F: Yes, yes, so I am asking to voluntarily take part in this study because of the knowledge you have gained through the process of treating your child and taking care of him or her at home. So maybe you would wonder what are the consequences you would have or what would be the consequences of participating in this research?

P: hmmmm

F: You may come to feel uncomfortable, maybe remembering the emotions you had when these things happened to you. Or you may even feel uncomfortable aahh, whenever you feel bad tell me [a child repeats me in the background) or whenever you feel you have a problem you can tell me to give you some time , all the time you might want

P: No, I really have no problem

F; yes you might also be wondering about what is the benefit of participating in this study. Although there are no guaranteed direct benefits for your participation in the study, as you mentioned before,the information collected will assist in the improvement of surgical neonatal care at CHUK and possibly help other parents who share similar experiences as yours..

But you would be given five thousand Rwandan Francs to help you travel to the place where you will meet the researchers.

You may also be wondering if I can stop this conversation whenever you want. yes! If you want to stop in the middle of it you just tell me.

You might tell me like hey, I don't feel comfortable anymore let's just leave it . That's possible!

Any information you may have will be used for research, but your name and profile will be kept confidential and we will not share it with anyone.

P; Hmmm yes

F: Yes. eeh you may have a problem and say like who will I ask among these people? to ask a question or give us any suggestion ooh I will send you phone numbers and / or emails. If you have a problem you will find a place to ask.

P: hmmm

F: Yes

F: So if you give me the right to interview you, then that is to say that you understand the content of this document

P: hmmm

F :you have had the opportunity to ask questions and you are satisfied with the information you have been given

P: hhmmm

P: hhmmm

F: When necessary, you took time to think about it or even talk to other people to help you make a decision. Are there any other people you would like to consult with before you take part in this study? I can give you time you know!

P: No other people I should consult.

F: hmmmm this means you give me the right to interview you?

P: No other people who would advise on this.

F; Yes, that means, you agree to participate in this study

P: Yes

F: : So did you agree that this interview between you and me can be recorded?

P: Absolutely yes, there is no problem

F: : Thank you very much. Thank you very much for agreeing to talk to me. We would like to use this information to help CHUK Hospital to make changes in the service they provide but as I have said earlier we will not disclose your name or profile to anyone even if you have given us permission.

Q: Did you have any problems before we start?

P: I have no problem!

F: That is alright!

F: so, let’s start with the first question that says maybe you would start by telling me about your time at CHUK. How many days have you been in the hospital? When did you enter? I mean you and your child, and when were you discharged from the hospital? ? Where were you from ?

P: Hmmm I live in the Matimba sector; I went to the Matimba health center when my water broke

F: yes

P: There they examined me and said that the baby was incorrectly lying in the womb, I was sent to Nyagatare General Hospital, and when I got there, the baby was lying in a bad position in the womb and from there they decided to do the C-section for me

F; eeeh It was a C-section then! Hmmmm

P: Hmmmm, after the C-section , they said to me, did you see that the baby you gave birth to has a problem ? They really explained it to me and they told me that I gave birth to a baby with the intestine outside. They added that for that reason, the baby needs to be transferred at CHUK General Hospital the very same day.

F: Yes

P: So I was still having the anesthesia due to the C-section. So, I could not go with the baby that day!

F: Yes

P: The child was immediately escorted by someone else to CHUK, and I was left in Nyagatare in the hospital. I was able to join the baby in two days. The baby was born on the thirty-first day of January

F: 31st of which year?

Q: The baby was born in 2020

F: eeh this is to mean it's been a year and five months wow!

P: that is true, it is one year and five months!

F: wow, thank God, yes

P: That's right

F: Hmmm

Q: At the time, I thought it was weird that I was asking God the reason why he could give a child like that while others have got normal children, I was like why , why did you do this to me o God." Fortunately I was feeling strong, and hopeful that he will live

F: Hmmm, Hmmm

P: When I got to the doctors , they really took care of him, they took good care of him except for one doctor

F: hmmm

P: Except for one doctor who works there at CHUK.n He is a man who works in the general ward, a man, He is really black, I don't remember his name.

F: Hmmm

P: You see they really wanted ..., you see the baby was fed by serums

F: Hmmm

P: They wanted veins through which serums pass. Then the doctor once tried to find veins and tried in the head but he could not find any vein. He put needles in the legs but could not find any vein for serum he tried everywhere and it refused.

F: hmmm

P: Now everywhere he injected needles was bleeding and bleeding and bleeding. Instead of the doctor looking for something to help the child to stop the bleeding from continuing to then he just keeps relaxing and stays there doing nothing. Then, I tried to cover the bleeding parts of the baby and there was too much blood so that the clothes I used were full of blood.

F: oohhh, hmmm

P: Then, that is the time when I saw the baby's lips become white, the tongue became white . Can you Imagine a situation where myself as a civilian got to the point where I saw that the child's blood was over?!

F: ooo!

P: That’s when I called the doctors around and they came and I saw that! They gave the baby basic help and I saw the baby doing better. Well, they treated him so well except only that day

F: You mean the baby?

*P: Sure, the baby*

*F: hmmm*

*P: When it came time for us to come home, we said goodbye, and he still had a bandage on the baby belly. so, when the day of our appointment came it was time for the Corona outbreak. That is when I called the doctor who used to treat him, and he advised me to take the baby to the district hospital so that they could remove the bandage.*

*F: hmmm*

*P: But there really is a problem that came to his belly. .Whenever he has to push with the belly, the belly button becomes bigger as if there is some air inside*

*F: hmmmm*

*F: Hmmmm*

*P: But the doctor told me that this belly button would not grow up and that there would be no problem for him to have an inflamed belly button. He added that I can take him for a surgery later if I want. But also, I can leave it like that if I want because there would be no problem with his inflaming belly button. .*

*F: Hmmm okay thank you so much! So , Hello!*

*P: yes I understand*

*F: You see as soon as you give birth to him, even though you got a C-section but you were watching.*

*Is this right?*

*P: Yeah that is true, They had given me anesthesia to the lower part of my body, but I could see and hear.*

*F: So after giving birth, the doctor showed you the baby you gave birth to, and while he was showing you the problem the baby was born with, how did you feel and what did you think and how did you react?*

*P: I was shocked and surprised , and I thought that never existed before our case because it was the first time I had seen it. I kept wondering about what happened to me. But as the hours went by, so did I, and the doctors came and approached me and told me that he was not the only one with that issue*

*F: Yes, yes, but what did the doctors tell you about the disease after you gave birth to the baby?*

*P: They just comforted me a lot and told me it wasn’t me alone who faced this.*

*F: but I'm CHUK,*

*F: Yes*

*P: The doctor explained to us that the fact that a child has an intestine outside, has nothing to do with witchcraft. He said that the baby was born like that because God had not yet finished fixing it. He just wanted to tell us that the GS is not poisonous. He wanted to tell us that they are things that just happen*

*F: Hmmmm yes so let me ask you about the time you and the baby were discharged ....how do you call him at home?*

*P: Imena Daruin*

*F:so, this means that you call him Daruin ?*

*P: sure, we call him Daruin*

*F: Let's talk about you and Daruin when you came from the hospital, when you came home, what plan did the doctors give you to take care of that child and how did you follow that plan.*

*P: Did you mean the plan to take care of the baby> right? It was all about going and avoiding disturbing and pressuring and avoiding telling him harsh words in every way possible .*

*F: Hmmmm*

*P: to just protect against all kinds of disturbing stuff in any every way possible*

*F: Even as soon as you were discharged when he was a really small baby . How did they tell you to take care of him?*

*Q: At a younger age?*

*F: What are the instructions that the doctor ordered you to follow while you were leaving the hospital? like he would say to treat the baby this way or that way! What did the doctor tell you to do in order to keep the baby safe?*

*P: They really told me to keep the belly button covered in bandages clean. They forbid me to wash the covered area and to avoid putting any water there. They told me how to clean it so that there would be no dirt. They told me that if there was dirt the situation would become worse... there would be... ... .. (She forgot what she wanted to mean) I forgot*

*F: Did you want to say infections?*

*P: Exactly! I wanted to mean that dirtiness on the covered area on the belly would get the baby infected, that's what they told me. The way I have to be really clean. It was all about the cleanliness they told me.*

*F: So do you think you followed those instructions?*

*P: Yes, I did*

*F: Hmmm,*

*F: The proof that I followed the instruction is because there was no problem from the time we were discharged until now, I followed it.*

*F: Yes yes. So what do you think of the health care or care provided to your child?*

*P: Help?*

*F: I mean What do you think of the medical care given to your child or how do you see it now?*

*P: I think they did me a favor. Well, they really did a lot of good for me! I could not imagine how a baby born with his intestine outside would survive! I never imagined how he would be able to live!' but they made this for us! They did well.*

*F: Hmmmm, Hmmmm*

*P: I do not see how to thank them better!*

*F: Yes Yes*

*P: I thought this was not going to be possible. The doctor told us at the doctor's office that the baby who was born with this condition had a 50 percent chance of survival and a 50 percent risk of death.*

*F: hmmmm*

*P: Because most of our neighbors at the hospital were dying.*

*F: Yes mom*

*P: The doctor added that you could even be discharged and even go home, and the baby dies a week later .*

*F: hmmm*

*P: I was always praying to God to keep him safe*

*F: hmmmm*

*P: Yes, yes*

*F: Hmmmm, thank God he protected him!*

*P: Yes he protected him*

*F: So another question I might ask you, have you ever noticed that your child needed urgent help ? Was there any time you realized your child's health needed a quicker attention to be taken care of? Explain for us please!*

*Q: hhhmmm?*

*F: There are times*

*P: hmmmm*

*F: Was there any time you saw that your child and he or she needed urgent care?*

*P: Hmmm, icyo gihe ava amaraso nkabona ururimi rwabaye umweru n’ iminwa yabaye umweru nabonags ko koko amaraso yamushizemo byararangiye, nkajya gutabaza*

*F: hmmm*

*P: Hmmm, at that time when he was bleeding and I saw the tongue turned white and my lips turned white and I saw that the blood was almost finished in his body . Then I went to seek the rescue*

*F: hmmm*

*P: That time, I went asking the doctors all over the place and they came in with blood transfusions to help my baby*

*F: hmmm*

*P: That's exactly what happened at the time*

*F: Hmmm so let me follow up on that question I asked you, can you tell me what it was like at home with Darwin, the time you were discharged from hospital and you started living home with a kid with the GS problem at home, can you tell me how you felt?*

*P: I was really strong! But people around the corners were just saying that this baby, that baby, will never make it to survive.*

*F: Hmmm*

*P: Also there were other people who used to predict that even if it happens and he survives, he would have mental disorders .*

*F: Hmmm*

*P: That was the case but really with the prayer I was able to be strong and move forward.*

*F: hmmm*

*F: Hmmm yes, thank God you! you really did great!*

*P: hhhmmm*

*F: Yes, something else I might ask you, can you share with us something that may have been difficult for you? Considering yourself at home or maybe the truth is in the hospital , you needed some kind of finances as soon as you gave birth to this baby, and even after, what happened to the job you were doing or are doing now? Have you ever had a mental or physical breakdown in your life with this situation?*

*P: Sure, I really struggled a lot. you see when you are discharged from the hospital, you just need a lot. Even at the hospital, there were medications, there were types of serums that we had to buy , and our mutuel de santé( a type of insurance used in Rwanda by many citizens)was not covering those medications. We had to buy these medications out of CHUK and the full cost was on us. So, when I was discharged, I went home with many debts. so I really went home in debt, and for that reason, there was land I had, and I sold it!*

*F: What? Land?*

*P: The share, the share I had, I had sold it.*

*F: HMmmm*

*YEs, I had a lot of debts: It was so very difficult for me, and the truth is I am really poor. It was very difficult for me to fight and pay those debts and everything else.*

*F: Hmmm, yeah ehh anyway you just talked about only means, did you have a job that had to be stopped or something else you were doing, or a job you had to lose have at the time? or you did not have a job at all?*

*Q: So the job I had was to sit on the street and sell tomatoes and onions. I had to place my table on the street and sell tomatoes and onions. Just that.*

*F: Hmmm*

*P: So when I got out of the hospital and came back, I could no longer even find five thousand Rwandan Francs to keep doing my small business. I could no longer sell those tomatoes and onions. , it was no longer possible, and I let it all go ! I did nothing else, I was simply right there.*

*F: Hmmm and whether it is mentally or physically do you think you were at some point disturbed by this situation? Did something like this happen?*

*P: No, nothing happened, I was strong!*

*F: Hmm, yes, you fought a man!*

*H: hmmm that's right! you did great!*

*F: So compared to other kids, Allo![ I thought I lost her on the phone]*

*P: Yes please !!*

*Q: Compared to other children, do you ... have other children?*

*P: No, I only have Daruin.*

*Q: How did you expect to raise a baby during pregnancy, and how do you feel about raising the baby after he was born? What is the difference? How would you describe it?*

*P: How did I do what?*

*Q: How did you prepare to raise a child while you were pregnant and how did you raise him when he was born?*

*P: Oh, the difference is, I feel no problem*

*F: hmm,*

*Q: I don't think I was too upset when I saw the baby for the first time because the doctors have explained so much to me about what was happening. I really was helped by them and was strong. and when we were about to go back home, the doctor assured me that my baby was like any other baby as long as he was healed. The doctor said my baby had no other problems. He was like all other kids.*

*F: yes yes*

*P: And he has no problem, he plays like all the other kids*

*F: hmmmm*

*P: Even those who used to say that he would live but he would be incomplete I think they are wrong because he is like all other children*

*F: Hmmmm*

*P: I raise him as normal, as I would raise him as I thought I would raise him when I was pregnant. That is how I still raise him today. Even if I still had to give away all of my possessions until I cannot take really good care of him......*

*F: hmmmm*

*P: But that's exactly what happens, I take care of him!*

*I take care of him as I thought I would when I was pregnant with him.*

*F: Hmmmm, yes! Thank you so much! So can you share with us how these things have changed your social life, for example if you have a husband, tell me, has he ever had a change in the relationship with you? In your relationships with your friends, in your relationships with your friends and relatives, and your neighbors, in society, did the fact that you have given birth to a child born with a GS disability have anything that it changed in your relationship with your spouse or in your relatives and friends? neighbors or society?*

*P: No, the relationship between the child's father has not changed. Except for those people, these neighbors all over the place who used to say even if t our son would live, he would live incompletely . For the child's father it hasn’t changed or it doesn’t matter because he feels that it is a natural thing. He doesn't even want to think about it! Because he is healed now we give thanks to God and the rest we put aside*

*F: Hmmm, Yes, hmmm, thank you very much. So, thank you for sharing with me your life, now can you tell me what the child is like? What is his life like? Is he eating well? Now, you see he is already a year and five months old.*

*P: Five*

*F: Does the child eat well? He grows up as well as any other child, how much he weighs, how much he weighs compared to others, please tell me how he is now*

*P: Age, weight*

*F: Hmmm*

*P: his lifestyle, and his behavior match his age*

*F: Hmmm*

*P: he eats with no problem,*

*F: Does he have an appetite?*

*P: He has a lot of appetite! hhh , he he eats a lot and I sometimes think I will miss enough food to feed him [the participant and facilitator laughing in the background]!*

*F: hmmm*

*P: Otherwise he has an appetite, no problem really.*

*F: hmmm Does he breastfeed with no problems?*

*P: he eats and breastfeeds without any problem, no problem, he always eats!*

*Q: As for his health, can you tell me if he is often sick or not?*

*Q: Hmmm? He is often sick!*

*F: hmmmm*

*P: For example, today he is vomiting, yesterday he had diarrhea, yesterday he had malaria . He is surely often sick!*

*F: hmmm but do you see him getting sick like other kids or do you see him getting sicker than others!*

*P: It's not too much but he's sick often.*

*F: Hmmm so can you tell me something you wanted*

*to know about your child or the care that you realized was needed for your child's sake?*

*P: Was attention needed?*

*F: hmmm maybe when you were pregnant or as soon as you gave birth to him and you realized he had the GS , have you ever said or thought there was some information you knew would have helped you?*

*P: Maybe if I was able to check with my pregnancy in the machine, I think I really would have nothing more to do with it, I would have to wait for the doctors to do their thing otherwise I would have nothing more to do.*

*F: hmmm, thank you. So what would you say to a parent with a child with the same problem as your child!*

*Q: What I would say is that he is patient and strong and ignores those who bully or discourage them and let things into God's and doctor's hands. [the voice is inaudible]*

*F; even?*

*P: The important thing is to be strong, that's normal in life, because I found out it's not an issue for only one person.*

*F: It is true that it is not for one person. Hmmm*

*P: That's what I told him*

*F: so do you have a question you would like to ask me or an idea you would like to give or anything else you have in mind*

*P: hmmmm, something I would have in my heart ?*

*F: Hmmm*

*P: I feel like nothing, everything, you have explained to me.*

*F: hhh?*

*F: Whatever I would have asked, you have already answered!*

*F: Yes thank you!*

*P: No problem*

*F: Yes, so let me thank you! I am hearing the baby crying too much! just a greeting from me and really keep up with the good work of raising him.*

*P: HHmm?*

*F: Greet him warmly, and thank you and have a nice evening*

*P: yes and goodbye [the baby crying in the background]*

*F: hmmm*

Recorder stopped: 3:56:07
