# Supplementary material for: Exploring the lived experiences of parents caring for infants with gastroschisis in Rwanda: The untold story
Source: PLOS Glob Public Health. 2022 Jun 15;2(6):e0000439. doi: 10.1371/journal.pgph.0000439 (PMC10021215; doi:10.1371/journal.pgph.0000439)
Supplement: S1 Data — (ZIP) [file pgph.0000439.s002.zip › S1_Data/S9_Text.docx]

**BB 5 ENGLISH TRANSCRIPT.**

**MODE: Thank you for allowing us to talk to you, Hello?**

W1: Yes

**MODE: I am saying thank you for allowing us to talk to you, Hello?**

W1: I can hear you.

**MODE: Can we start now?**

W1: Yes

**MODE: I was saying thank you for accepting to converse with us, the purpose of this interview is to find out how your child was taken care of and how you also took care of him when you were at the hospital, and when you left the hospital.**

W1: Hmm

**MODE: We would like to use this information to help CHUK make changes in service delivery but we will not reveal your names as I had confirmed it before or reveal your identity to anyone. Do you have any question before we start?**

W1: No question

**MODE: Okay, raise your voice please**

W1: Yes

**MODE: When was your child admitted in the hospital?**

W1: I do not recall the date but he spent there one month.

**MODE: Is it this year or the previous year?**

W1: It is this year in April, he went there in April.

**MODE: You went there in April?**

W1: Yes

**MODE: What was the span between giving birth and taking him to the hospital?**

W1: It was just after one day of birth, I went there and spent a month.

**MODE: It had been how many days?**

W1: One day

**MODE: Yes, for how long did you stay at CHUK?**

W1: One month

**MODE: Your child was a boy or a girl?**

W1: He was a boy.

**MODE: Okay, I would like to start from the moments you had at CHUK in April this year when you and your child had gone to CHUK.**

W1: It was on 5^th^ this year.

**MODE: Of this month?**

W1: Yes

**MODE: That is when you were at CHUK?**

W1: Yes

**MODE: Okay, can you tell me in details what happened when you were at the hospital, what happened in that month since you arrived till you left.**

W1: I did not face any problem, they assisted me in all possible ways, since I did not pay for the hospital bills.

**MODE: You did not pay?**

W1: Yeah, the challenging part was in photocopying and medicine that I bought out of the hospital.

**MODE: Why didn’t you pay?**

W1: I am in the first category?

**MODE: Ooh the first Ubudehe category?**

W1: Yes

**MODE: Okay, that is what happened in the first month? I would like you to share with me your monthly journey at the hospital, how it went day by day. Even though you may not tell daily details, you can give an overview of what happened in that month.**

W1: My greatest concern was seeing children die and I would tell myself that mine will die as well, but I saw that the doctors were doing their best.

**MODE: Hmm**

W1: They could come like today and check, and if I saw any change, I could alert them and they could come to check. The doctor could also come to check, and he could tell me that the child will recover. They later discharged me and told me that he has no problem, and I went home. When I arrived at home, they told me to go to the hospital, and I spent there one week.

**MODE: Why did they tell you to immediately go to the hospital?**

W1: Yeah, I spent there one week.

**MODE: Yeah, I am asking you the reason to why you left CHUK and they told you to go to the hospital when you reach at home?**

W1: The wound had scared the doctors, and he was very tiny.

**MODE: He was very tiny?**

W1: Yes

**MODE: Hmm**

W1: After that, I were to get to the health center twice a week.

**MODE: And you go where?**

W1: To the health center for wound covering twice a week.

**MODE: Hmm**

W1: In the middle of that process, he left us after 2 months and a half.

**MODE: Hmm, okay. After giving birth to that child, how did you react when you saw that he had external intestines?**

W1: I saw that it was unusual but the doctors told me that he would recover, and I believed it. I spent time there and they could comfort me as they told me that the baby will recover, and I left the place with him when he was alive.

**MODE: Hmm**

W1: So I knew that he would return since he was at home by then.

**MODE: What did they tell you that caused him to be born with external intestines?**

W1: They did not tell me the cause.

**MODE: After giving birth, didn’t they tell you anything at the hospital?**

W1: No

**MODE: How did it proceed?**

W1: I was the only one who had given birth to a child with external intestines, that is when they immediately took me to the hospital.

**MODE: When you reached at CHUK, what did the doctors tell you about the sickness of your child?**

W1: They didn’t tell me that it was a catastrophe because I wasn’t the only one there, I found there other people.

**MODE: And they told you what?**

W1: They told me that he will recover.

**MODE: You told me that you spent a month in the hospital, right?**

W1: Yeah

**MODE: What financial challenges did you face or any other challenges related to the process of treating your child in the month that you spent at CHUK?**

W1: There was no challenge, I was on good terms with the doctors, I could tell them any challenge that I had, and they could help me.

**MODE: What kind of help would they provide?**

W1: They could come to check the problem that he has, and if it is low oxygen, they could add it.

**MODE: What relationship challenges did you face with the people that you met at the hospital?**

W1: I didn’t have any challenge; I saw it normal to accept such circumstances.

**MODE: What good thing did you find at CHUK when you brought your child?**

W1: There wasn’t anything bad.

**MODE: What good thing did you find, like if someone asked you about the services you received, what would you say that you liked from CHUK? Or what good things did you experience?**

W1: It was that I met with a person who assisted me when I had no money, and he could help me by all means, I could call him and he could send money. I never experienced hunger even though I had no family to bring me food. I also want to thank the doctors who took care of me and did everything.

**MODE: Okay, after getting discharged from the hospital with your child, which plan did they give you for taking care of your child? And how did you follow it?**

W1: They told me to keep the child from poor sanitation and told me to protect him from getting cold. They told me that I have to take him to the hospital for wound covering twice in a week, and give him the prescribed medicine on time, and provide the right dose. They told me to clothe him with many clothes for warmth, and to breastfeed him sufficiently.

**MODE: And to do what?**

W1: Breastfeed him sufficiently.

**MODE: Yes, raise your voice**

W1: I was talking about breastfeeding him sufficiently.

**MODE: Yes, what do you think about the services you received when you took the child at the hospital, as they told you that you have to take him twice a week for wound covering. What would you say about the services you received?**

W1: I at first had issues with the doctors because they could lie to me because they could say that they won’t treat him, or that they will take him in the surgery room, yet he wasn’t yet allowed to get there. They could tell me that and I could come back, and wherever they sent me, they could refuse, and they later got used to me, and they started providing good services.

**MODE: At first they refused?**

W1: Yes, they said that he was too young for that.

**MODE: Too young for what?**

W1: That they can’t take him to the surgery room when he is still a baby.

**MODE: Did they later change and start helping you?**

W1: Yes, there was no problem and they assisted me.

**MODE: Was there an emergency situation that happened to your child when you were at home that made you seek immediate medical assistance/ take him to the hospital?**

W1: No

**MODE: Okay, going back to what we were discussing, can you tell me your experience of living at home with your child after getting discharged from the hospital?**

W1: I followed the instructions, and they told me to avoid stress since it causes the loss of breastmilk, and the husband could help me to provide for everything, and he…

**MODE: And he?**

W1: My task was to take care of the child.

**MODE: Hmm**

W1: I breastfed him whenever it was necessary, and I could find clothes for warming him, and following the given instructions.

**MODE: When you reached at home, can you share with us the financial challenges that you faced?**

W1: There weren’t any.

**MODE: How about in your work?**

W1: I wasn’t working, since the time I left the hospital, I hadn’t gone back to work.

**MODE: And it didn’t even affect your finances?**

W1: Yes

**MODE: So, what was challenging, did you have any mental depression?**

W1: No, the doctors had told me that I don’t have to pay, and they could comfort me. I never had a challenge.

**MODE: So, you have had how many children so far?**

W1: Hmm

**MODE: You have had how many children?**

W1: This one was the second, the first child it was a miscarriage.

**MODE: The first one it was a miscarriage?**

W1: Yes

**MODE: Considering how you thought of your birth moment and what happened after giving birth, when you realized that he had external intestines, what was your say?**

W1: I don’t have much to say on that because I saw that anything can happen, I may not be the only one, and I may not be the first one to hear it. I accepted my situation and showed it to God, and I ignored the rest to avoid getting problems.

**MODE: Did the occurrence change the relationship with your husband or the society where you lived?**

W1: No, there was no problem, aside from the words of women. Other instances were just normal and there was no problem.

**MODE: What were those women saying?**

W1: They said that it occurred as a result of worrying, and I could respond to them,” why is it that others worry but they don’t face a similar situation?”

**MODE: Hmm**

W1: I just ignored their words.

**MODE: Okay, during that time when you had returned home, how did you see the health of your child?**

W1: He had no problem, and I had hope for his recovery.

**MODE: Did he cry frequently? How was it?**

W1: No, he could sleep, even from his birth, he slept a lot.

**MODE: Hmm, did he suffer from diarrhea?**

W1: No, he didn’t suffer from any other disease.

**MODE: Even vomiting, he didn’t vomit?**

W1: Apart from the moment when the intestines were not yet fixed in his belly.

**MODE: When was that?**

W1: When I was at CHUK.

**MODE: That was when he used to vomit?**

W1: Yes, but I left the place when it had ceased.

**MODE: Did you only breastfeed him or you also gave him milk?**

W1: I only breastfed him.

**MODE: How was his growth compared to…?**

W1: His weight was increasing from 2.5 and nearing to 3.

**MODE: He was born with how many kilograms?**

W1: He was born with 2.200, and it later backslid to 1.400.

**MODE: When did it backslide?**

W1: When we were at the hospital, it backslid to 1.200, and when I went home, it increased to 2.20, and before his death, he weighed 3.200.

**MODE: Yes, did you find him to be normal like other children?**

W1: Yes, he had no problem.

**MODE: Was there any change in his height?**

W1: He was very tall, his height at the hospital was not the one that he had after reaching home.

**MODE: Okay, I am so sorry for you, you told me that your child is no more.**

W1: Yeah

**MODE: Can you tell me the testimony of what happened that resulted in the loss of his life?**

W1: Yes

**MODE: Yeah, what happened?**

W1: The way it happened?

**MODE: Yes**

W1: I woke up and when I was going to clothe him, I realized that he had passed away.

**MODE: Hmm?**

W1: I turned him when I was going to breastfeed him and I found that he had passed away.

**MODE: Hadn’t he fallen sick before that, hadn’t he faced any problem?**

W1: He had not suffered from any disease like the way it happens with other children.

**MODE: When did that happen, when did he pass away?**

W1: I gave birth to him in April and he died two months later, in June.

**MODE: It’s in this month?**

W1: Hmm?

**MODE: He died in this month?**

W1: Yes

**MODE: You don’t recall the date?**

W1: No

**MODE: Did you take your child to the hospital after finding out what happened to him?**

W1: After his death?

**MODE: Hmm**

W1: I took him and they told me that he had died, and I took him back for burial.

**MODE: What do you wish to have known about the life of your child or the care that he needed?**

W1: There is no problem.

**MODE: No, isn’t there anything you wish to have known before?**

W1: I didn’t know anything.

**MODE: No, you didn’t know, but isn’t there something you wish to have known before what happened, before the pregnancy, after giving birth, isn’t there any information you would wish, like when one reflects and regrets, and says, “I wish I had known this and that.” Didn’t you have such thoughts?**

W1: No

**MODE: What can you tell a fellow parent who has a child with a problem that is similar to what your child had?**

W1: It is to have perseverance and acceptance, and to pray because prayer strengthens your heart. The rumors pass you by and you don’t mind about them, in brief you have no worries.

**MODE: What would you tell or advise to the doctors of the place where you bore him, or those of CHUK? What advice would you give to them basing on the moments that you went through?**

W1: I didn’t have any problem with them, what I would tell them is to encourage them to keep up their good work because they became people that one would desire.

**MODE: Okay. Do you have any other question or additional point?**

W1: No

**MODE: Our conversation is nearing to the end, but before we wind up, if you have a question or suggestion, you may say it.**

W1: No, I have no question. Instead, I request you to greet for me all the doctors who were in that hall.

**MODE: Hmm**

W1: Greet for me the tall doctor and also the short one.

**MODE: Hmm**

W1: Especially those doctors who are mothers.

**MODE: Okay, thank you. May you have a good time.**

W1: Yes, likewise.

**MODE: Yes.**
